# The efficacy of parenting interventions for forced migrant families on child internalizing and externalizing symptoms, parental self-efficacy, and parental competence: A systematic review and meta-analysis

**DOI:** 10.1177/13634615251372854

**Published:** 2026-03-25

**Authors:** Maja Västhagen, Clover Jack Giles, Anna-Clara Hollander, Ata Ghaderi, Livia Van Leuven, Anna Edenius, Pia Enebrink

**Affiliations:** 1 27106 Karolinska Institutet, Sweden; 2 6233 Örebro University, Sweden; 3 Center for Epidemiology and Community Medicine, Stockholm County, Sweden

**Keywords:** forced migration, meta-analysis, parenting programme, prevention, intervention

## Abstract

Forced migration has reached unprecedented levels worldwide. Involuntarily migration creates stressors for families that require systematic action at a societal level. Our premise is that parenting programmes have an untapped potential to enhance psychosocial health among parents and children. The aim of this study, therefore, was to review existing studies to evaluate the efficacy of preventive parenting programmes for parents who were refugees, asylum seekers or internally displaced. We included 20 publications from 3 electronic databases (Ovid MEDLINE, Ovid PsycINFO and Web of Science Core Collection): 16 on parenting interventions (13 original trials, *N* = 1191) and 4 on combined interventions (parents and youth, *N* = 1284). We compared a range of outcomes including child internalizing and externalizing symptoms, as well as parental competence (positive/negative parenting), self-efficacy, well-being and mental health/psychological distress. Between-group analyses indicated less externalizing behaviour (Hedge's *g* = 0.43, *p* < 0.05, k = 2) at post measurement for parents participating in the interventions, compared with those in the control conditions, as well as enhanced positive parenting strategies (*g* = 0.89, *p* < .01, k = 2), self-efficacy (*g* = 1.94, *p* < .001, k = 2) and parental psychological distress (*g* = 0.67, *p* < .05, k = 4). Within-group analyses of pre and post measurements supported that parents participating in the interventions reported improvements over time in all primary outcomes: parent-rated child internalizing and externalizing behaviours, negative and positive parenting, self-efficacy and the secondary outcome psychological distress. Analyses of combined studies suggested a small increase in positive parenting strategies (*g* = 0.17, *p* < .05, k = 2). Although our sample of reviewed studies was relatively small, and the study outcomes varied considerably, the results indicate that parenting programmes might be an underutilized resource to promote health among forcibly displaced families.

Systematic review registration: PROSPERO (CRD42022330521).

## Introduction

The United Nations Sustainable Development Goals (SDG) have been signed by 193 Member States, and include a focus on reducing inequalities and injustice, ending extreme poverty, and addressing the problems of climate change. Improving services and welfare equity for populations that are vulnerable or hard to reach is among the United Nations SDGs. This goal targets, among others, the 117 million forcibly displaced people worldwide (United Nations High Commissioner for Refugees [UNHCR], 2024; August 26). Forcibly displaced people are a particularly vulnerable and underserved group. In addition to the universal social determinants of health (Lund et al., 2018) migration is associated with specific determinants, both related to the migratory experiences, and resettlement in a new culture and context (Bornstein, 2017). Moreover, forced migration is associated with distressing and sometimes traumatizing experiences such as violence (Jolof et al., 2022), lost or damaged trust (Essex et al., 2022) and dangerous unofficial migration routes (Fargues, 2017). However, although social determinants of health in general, and pre-migration experiences in particular, have been seen to affect refugees’ well-being, research also suggests an important correlation between poor mental health and post-migration stressors experienced in the resettlement context (Chen et al., 2017). Indeed, the post-migration context can be an equally powerful determinant of mental health, and it plays a crucial role in moderating an individual's ability to recover from pre-migration trauma (Hynie, 2018). For example, Hou and colleagues (2020), found that daily post-migration stressors, such as discrimination, isolation or accommodation difficulties, had a stronger association with poor mental health than did past traumas.

Despite the considerable challenges of forcible displacement, and the increased risk of poor mental health (Blackmore et al., 2020), most forcibly displaced people who have permanently resettled do not suffer from mental health disorders. Rather, they show remarkable resilience. Resilience is a human capacity that refers to the ability to respond to adversities with preserved or a swift return to function (Masten et al., 2021; Vella & Pai, 2019). Increasing empirical attention to resilience has accompanied a shift in clinical focus, away from a deficit-based model of mental illness to a strengths- and competence-based model with a preventative approach (Masten, 2014; Motti-Stefanidi & Masten, 2017; Southwick et al., 2014). Moreover, because resilience is understood to be malleable, it is a relevant target for preventative and mental health promotive psychosocial interventions (Siriwardhana et al., 2014). For example, positive relationships and social support are key to resilience (Southwick et al., 2016). Thus, the systems in which individuals are embedded (e.g. family, community) influence their resilience and psychological functioning (Southwick et al., 2016). By enhancing the supportive qualities of a social system, it is possible to increase the resilience and psychological health of the individuals within it.

In this study, we defined parenting programmes as psychosocial interventions designed to enhance resilience in parents and children by providing support in the parental role, often through the practice of positive parenting skills. These programmes ultimately aim to improve the psychological well-being of parents and their children. Outcomes include, for example, reductions in children's internalizing (Costantini et al., 2023) and externalizing behaviours (Mingebach et al., 2018), enhanced parental skills and psychosocial health (Barlow et al., 2002), and increased parental self-efficacy (Ulfsdotter et al., 2014). It has been suggested that scaling up preventative and promotional parenting programmes has an untapped potential to address many of the SDGs (Sanders et al., 2022).

As parenting programmes have gained traction and are spread between increasingly multicultural societies, interest in parenting programmes adapted or specifically developed for immigrants and ethnic minorities has evolved (see Hamari et al., 2022 for a review). Some of these focus on refugees and others on forcibly displaced families. However, little systematic knowledge exists about the efficacy of promotion and prevention interventions for refugees, asylum seekers and internally displaced people in general (Uphoff et al., 2020), including parenting programmes for this group (Wieling, 2018). Overviews of psychosocial interventions for refugees and asylum seekers (systematic review, Tribe et al., 2019; review and meta-analysis, Turrini et al., 2019), and of family interventions for traumatized immigrants and refugees (systematic review, Slobodin & de Jong, 2015) do exist. Further, one systematic review of parenting programmes for forcibly displaced populations (Gillespie et al., 2022) has been published, concluding that parenting programmes have a potential to mitigate the effects of the displacement within families. To our knowledge, no meta-analysis of parenting programmes for this population has yet been conducted. The aim of this study was therefore to systematically evaluate available empirical knowledge regarding the efficacy of health promoting and/or preventive (universal, selective) interventions targeting forced migrant parents.

The objectives of this study were to:

1. Evaluate the efficacy (between-group effects) of health promoting and/or preventive parenting programmes for forced migrant parents in reducing child externalizing and internalizing problems and promoting parental self-efficacy and competence (primary outcomes).
2. Evaluate the efficacy of the preventive programmes for improving parental well-being and reducing the risk for depression-, anxiety-, stress- and trauma-related symptoms (secondary outcomes) among parents.
3. Evaluate within-group changes of the same primary and secondary outcomes for the health promoting and preventive parenting programmes over the intervention period (pre–post) and at follow-ups.
4. Examine possible moderators and predictors (e.g. continent where the treatment is provided, treatment components, cultural tailoring) of outcomes.
5. Describe treatment components and cultural tailoring.

## Method

### Protocol and registration

This systematic review and meta-analysis was conducted and reported according to the PRISMA guidelines (Moher et al., 2015). The protocol for this meta-analysis was pre-registered at PROSPERO (CRD42022330521).

### Eligibility criteria

The PICO model was used to define inclusion criteria. First, the population was forced migrant parents, i.e. refugees, asylum seekers and internally displaced persons. Second, the interventions of interest were health promoting and preventative parenting programmes (universal or selective) delivered in any format (e.g. individually, in a group format, through the internet). The interventions should have a psychological or psychosocial theoretical foundation to be included. Interventions were included, regardless of whether they had been developed locally or adapted to the specific context in which they were delivered. In some prevention programmes both parents and their children receive an intervention. These combined interventions were also included, because we intended to capture all programmes addressing parenting skills. The inclusion of combined interventions was not described in the pre-registered protocol. To reduce the risk of confounding because of the child intervention sessions, we performed separate analyses of parent-only and combined interventions. Studies with clinical symptom levels as inclusion criteria were excluded, as were those implemented in clinical settings. Randomized controlled trials (RCTs; including cluster randomized), non-randomized controlled trials and (for the third study aim) uncontrolled trials with pre–post study design were included. Third, all types of comparators were included: no intervention (including waiting-list), psychological placebo, ‘treatment as usual’ and other interventions. For the third study aim, non-randomized controlled trials and pre–post study design without control group were also included. For non-randomized trials we included studies with and without a control group to evaluate changes over time (within-group changes). Fourth, the primary outcomes were child externalizing and internalizing symptoms as well as increased parental self-efficacy and competence. We included positive parenting (e.g. warmth, responsiveness, parental involvement) and negative parenting (e.g. harsh parenting, inconsistent discipline, poor supervision), which is an accepted way of conceptualizing parental competence (Reparaz et al., 2021). The secondary outcomes were parental well-being, reduced risk for depression-, anxiety-, stress- and trauma-related symptoms as well as psychological distress. Only variables measured with psychometrically validated scales were eligible. Studies published in Swedish, Norwegian, Danish, French, German or English were included. No restrictions were placed on date, type of publication, or geographical region, to cover all relevant studies.

#### Exclusion criteria

Studies were excluded if the intervention targeted parents of youth aged >18 or if the number of participants in any group was *n* ≤ 10. Interventions that were not of a psychosocial character (e.g. medical) were not eligible. Studies including psychosocial interventions identified as indicated prevention or treatment, defined as parents with clinical levels on any of the outcome measures, or implementation in a clinical setting, were excluded.

### Information sources and search strategy

The search strategy was developed together with two librarians (Appendix 1) and was used for two separate systematic reviews and meta-analysis (Giles et al., 2024). Four major concepts were included: (1) parental refugee populations, (2) mental health-related outcomes including parental self-efficacy and parental competence, (3) psychosocial interventions, and (4) RCTs and uncontrolled study designs. Each concept was elaborated and combined with free-text search, truncated and combined with proximity operators (Appendix 1). An electronic systematic literature search was performed on two occasions by librarians at the Karolinska Institute University Library in the following databases: Ovid MEDLINE, Ovid PsycINFO, Web of Science Core Collection. Databases were first searched on 10 May 2021 and this search was then updated on 8 April 2022. Additionally, two authors (A1 and A2) screened reference lists of the included studies, former reviews and meta-analyses, and the Web of Science Citation Index, to identify missed articles that may have fulfilled the inclusion criteria.

#### Study selection

**Figure 1.**
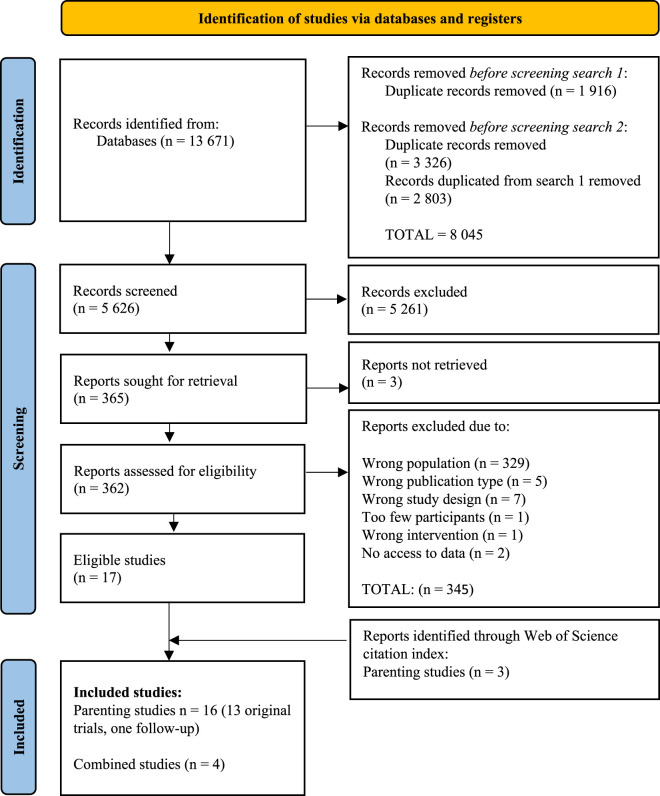
PRISMA 2020 flow diagram of inclusion process.

To ensure that the inclusion criteria were fulfilled the entire inclusion/exclusion process was completed by two pairs of independent reviewers (A1 and A2, A4 and A5). Rayyan was used for screening of abstracts. Abstracts approved by two blind reviewers were thereafter imported to Endnote for full-text review by the same pairs. Exclusion criteria identified during full-text review were reported for each study. After full-text review, studies fulfilling the inclusion criteria according to both reviewers were included. If mutual consensus regarding inclusion of a study could not be reached, it was resolved by discussion with a third part (A7). The process is reported and presented in the PRISMA template (Figure 1).

### Data extraction process

When inclusion of studies was complete, data were extracted to Excel. Data extraction was performed independently and then checked collaboratively by A1 and A2. From each selected study, the following information on the study type and population was extracted:

- study title, author, year and journal;
- study characteristics – design, recruitment procedure and study quality;
- participant characteristics – legal status, reason for migration, language, level of education, housing situation, time in host country, age, percentage men/woman, ethnicity and number of children;
- intervention and setting – type and level of intervention, country and context, duration of the intervention, types of comparators, intervention leader, intervention components, information about cultural adaptations made; and
- outcome data – type of outcome measure, statistical techniques used, the mean score and standard deviations at pre, post and follow-up measurements, length of follow-up, type of analysis (intention to treat or study completer analysis), number of participants enrolled and then included in analysis, attrition.

### Assessment of risk of bias

The risk of bias of the included studies was assessed independently by two reviewers (A1 and A2) using Cochrane Collaborations’ tool for RCTs (Sterne et al., 2019) and ROBINS-I for pre–post studies (Sterne et al., 2016). These tools assess a variety of possible biases in the trials, such as random sequence generation and allocation concealment (selection bias), blinding of participants and personnel (performance bias), blinding of outcome assessment (detection bias), attrition rate, reporting biases and other biases. The authors evaluated the presence of sufficient information regarding each criterion and addressed the likelihood of bias. A pilot assessment was conducted to ensure interrater reliability by three of the authors (A1, A2, A7). Two of the authors then continued the assessment and the senior author (A7) was consulted to resolve discrepancies in the assessment of bias if consensus could not be reached. The overall result of the risk of bias assessment is presented for each included study.

### Meta-analysis and synthesis of results

The principal study measures were between-group differences in means or effect sizes in RCT studies post intervention. Secondary study measures were differences in means between pre and post intervention reported in all study types. Intervention means and standard deviations for pre and post intervention, and/or effect sizes for all variables of interest were extracted. The outcome measure closest in time to post intervention was used as post data if not explicitly described as such. Outliers and residuals were investigated.

All statistical analyses were completed using Comprehensive Meta-Analysis version 4 (Borenstein, 2022). The overall programme effect was estimated for each eligible outcome measure (outcome present in two or more studies). Random effects meta-analyses were conducted to pool mean differences for our continuous outcomes from the validated rating scales and visualized in forest plots. *I*^2^ and *Q* statistics were used to assess statistical heterogeneity among the studies. For the *Q* statistics a *p*-value of < 0.10 represents heterogeneity. The *I*^2^ statistic, is based on *Q* statistics, and describes the percentage of variation across studies that is due to heterogeneity rather than chance. Up to 25% is considered low heterogeneity, 50% moderate heterogeneity and 75% high heterogeneity. Sensitivity analyses were planned to be conducted when outliers, or large residuals were identified, and when study weighting was very uneven. Between- and within-group effects were reported for outcomes present in at least two comparable studies. Based on Cochrane recommendations (Deeks et al., 2023), no subgroup analyses were performed, due to low number of studies. Publication bias was assessed using Egger's test and visualized in funnel plots. The trim-and-fill method was applied.

### Certainty of evidence

Certainty of evidence was assessed as high (⨁⨁⨁⨁), moderate (⨁⨁⨁◯), low (⨁⨁◯◯), or very low (⨁◯◯◯), using the GRADE system (Schünemann et al., 2008) for RCT studies.

## Results

### Identification and inclusion of studies

The inclusion process is reported and presented in PRISMA flowchart (Figure 1). The two searches together resulted in 13,671 reports. Duplicated reports were removed (*n* = 8,045) and 5,626 abstracts were screened. Thereafter, 365 studies were read in full-text and considered for inclusion (see Appendix 2 for studies considered for inclusion and reason of exclusion). Three additional studies were identified through screening of reference lists, the Web of Science Citation Index for the included studies and other relevant systematic reviews and meta-analysis. Finally, 16 publications of parenting interventions and 4 publications with combined interventions were included.

### Study characteristics

In total, there were 16 studies (including 2 additional publications based on 2 trials, and 1 follow-up study) of 13 parenting trials (5 RCTs, 8 pre–post studies). Further, there were 4 studies with combined interventions (3 RCTs, 1 pre–post study). The studies were published between 2001 and 2022.

#### Parenting interventions

Study characteristics are presented in Table 1. Bjorknes and Manger (2013) and Bjorknes and colleagues (2015) reported different outcomes from the same original study, as did Osman, Flacking et al. (2017) and Osman, Salari et al. (2017). Osman et al. (2021) report 3-year follow-up data for the same study. Of the 16 parent intervention studies, 7 were RCTs at the parent/family level (Bjorknes & Manger, 2013; Bjorknes et al., 2015; Dybdahl, 2001; Miller et al., 2020; Osman, Flacking et al., 2017; Osman, Salari et al., 2017; Shaw et al., 2021). One study had a within-subject experimental design (Eltanamly et al., 2022) and one study had a non-randomized controlled design (Morris et al., 2012). The remaining six studies were uncontrolled pre–post treatment studies (Ballard et al., 2018; Husby et al., 2020; Kaptan et al., 2022; Lakkis et al., 2020; Renzaho & Vignjevic 2011; Sim et al., 2020). Osman et al. (2021) was a pre–post 3-year follow-up of the intervention studied by Osman, Flacking et al. (2017) and Osman, Salari et al. (2017).

**Table 1.** Study characteristics of parent and combined interventions: outcomes, measures and risks of bias.

Author and year	Title	Study type	*N* included (total = 5741)	Outcomes within scope of review and meta-analysis (rater)	Instrument used to assess outcome	RoB
**Parenting study **						
RCT						
Bjorknes and Manger (2013^a^)	Can parent training alter parent practice and reduce conduct problems in ethnic minority children? A randomized controlled trial	RCT	96 (Int. = 50, WLC = 46)	Positive parenting (parent), Negative parenting (parent), Child externalizing (parent)	Parent Practices Interview, Eyberg Child Behaviour Inventory	Mod
Bjorknes et al. (2015^a^)	Exploring mental distress among immigrant mothers participating in parent training	RCT	96 (Int. = 50, WLC = 46)	Psychological distress (parent)	Hopkins Symptom Checklist	Mod
Dybdahl (2001)	Children and mothers in war: an outcome study of a psychosocial intervention program	RCT	75 (Int. = 35, TAU = 40)	Trauma (parent), Child internalizing (depression) (child), Child internalizing (anxiety and sadness) (parent)	Impact of Events Scales, Birleson’s Depression Inventory, Child problems	Mod
Miller et al. (2020)	Supporting Syrian families displaced by armed conflict: a pilot randomized controlled trial of the Caregiver Support Intervention	RCT	151 (Int. = 78, WLC = 73)	Psychological distress (parent), Parent and child well-being (parent)	Kessler Psychological Distress 10, Warwick Edinburgh Mental Wellbeing Scale, KIDDY/KID KINDL (child)	Mod
Osman, Flacking et al. (2017^b^)	A support program for Somali-born parents on children’s behavioural problems	RCT	109 (Int. = 57, WLC = 52)	Child internalizing and externalizing symptoms (parent)	Child Behaviour Checklist Symptoms	Mod
Osman, Salari et al. (2017^b^)	Effects of a culturally tailored parenting support programme in Somali-born parents’ mental health and sense of competence in parenting: a randomized controlled trial	RCT	109 (Int. = 57, WLC = 52)	Psychological distress (parent), Self-efficacy (parent)	General Health Questionnaire, Parenting Sense of Competence Scale	Mod
Osman et al. (2021^c^)	Impact of a culturally tailored parenting programme on the mental health of Somali parents and children living in Sweden: a longitudinal cohort study	Follow-up from Osman, Salari et al. (2017)	51	Child internalizing and externalizing behaviours (parent), Psychological distress (parent)	Child Behaviour Checklist, General Health Questionnaire	Mod
Shaw et al. (2021)	A randomized clinical trial testing a parenting intervention among Afghan and Rohingya refugees in Malaysia	RCT	137 (included in RCT) (Int. = 47, WLC = 32; study completers)	Psychological distress (parent), Self-efficacy (parent), Positive parenting (parent), Negative parenting (parent)	The Refugee Health Screener-15, Child Adjustment and Parent Efficacy Scale, Alabama Parenting Questionnaire-Short Form	Mod
Pre–post study						
Ballard et al. (2018)	Feasibility of implementation of parenting intervention with Karen refugees resettled from Burma	Pre–post study	11	Parent anxiety, trauma and depression (parent), Negative parenting (parent and child), Positive parenting (parent and child), Child externalizing behaviours (parent), Child internalizing (child and parent)	Karen Mental Health Screening Instrument, The Child Depression Inventory, Strengths and Difficulties Questionnaire, The Parent/Youth Issue Checklist	Ser
Eltanamly et al. (2022)	Strengthening parental self-efficacy and resilience: a within-subject experimental study with refugee parents of adolescents	Within-subject experiment design	53	Self-efficacy (parent)	Me as a Parent	Mod
Husby et al. (2020)	Prevention of trauma-related mental health problems among refugees: a mixed-methods evaluation of the MindSpring group programme in Denmark	Pre–post study (mixed methods)	32	Well-being (parent)	WHO-5 Index	Ser
Kaptan et al. (2022)	Online delivery gave me privacy and distance from others: feasibility trial and qualitative evaluation of an online intervention for refugee and asylum seekers; LTP + EMDS G-TEP	Pre–post study	14	Parent: trauma symptoms, anxiety, depression (parent)	International Trauma Questionnaire, Generalized Anxiety Disorder-7, The Patient Health Questionnaire-9	Ser
Lakkis et al. (2020)	A pilot intervention to promote positive parenting in refugees from Syria in Lebanon and Jordan	Pre–post study	50	Child internalizing (parent), Child externalizing (parent), Negative parenting (parent), Child well-being (parent)	Strengths and Difficulties Questionnaire, Disciplinary style questionnaire, WHO Wellbeing Index-5	Ser
Morris et al. (2012)	Does combining infant stimulation with emergency feeding improve psychosocial outcomes for displaced mothers and babies? A controlled evaluation from northern Uganda	Non-randomized controlled trial (TAU)	132	Positive parenting (parent)	Acholi Home Observation Measurement	Mod
Renzaho and Vignjevic (2011)	The impact of a parenting intervention in Australia among migrants and refugees from Liberia, Sierra Leone, Congo, and Burundi: Results from the African Migrant Parenting Program	Pre–post study	39	Negative parenting (parent)	Revised adult–adolescent parenting inventory	Mod
Sim et al. (2020)	Acceptability and preliminary outcomes of a parenting intervention for Syrian refugees	Pre–post study	292 parents, 88 children	Negative parenting (parent and child), Child internalizing symptoms (child), Trauma symptoms (parent), Psychological distress (parent)	Multiple Indicator Cluster Survey Discipline Module, Parental Acceptance Rejection Questionnaire, Short Mood and Feeling Questionnaire, Screen for Child-Anxiety-related Emotional Disorders, PTSD Checklist, Depression, Anxiety and Stress Scale	Mod
**Combined study**						
RCT						
Akhtar et al. (2021)	Feasibility trial of a brief scalable psychological intervention for Syrian refugee adolescents in Jordan	RCT	59 families [59 children (Int. = 33, Enhanced TAU = 26) 59 caregivers]	Externalizing symptoms (parent), Child depression (child), Well-being (child), Psychological distress (parent), Positive and negative parenting (parent)	Pediatric Symptom Checklist, Patient Health Questionnaire, Warwick Edinburgh Mental Wellbeing Scale, Kessler Distress Scale 6, Alabama Parenting Questionnaire	Mod
Betancourt et al. (2020)	Family-based mental health promotion for Somali Bantu and Bhutanese refugees: feasibility and acceptability trial	RCT	111 children and caregivers 49 children (Int. = 24, TAU = 25) 62 caregivers (Int. = 29, TAU = 33)	Depression (child and parent), Child externalizing (child and parent)	Center for Epidemiology Studies Depression Scale for Children, Achenbach Youth Self Report	Mod
Puffer et al. (2017)	The impact of a family skills training intervention among Burmese migrant families in Thailand: a randomized controlled trial	RCT	479 households, *N* = 1284 Int. = 240 households (240 children, 256 parents) WLC = 239 households (239 children, 257 parents)	Positive and negative parenting (child and parent)	Discipline Interview	Mod
Pre–post						
El-Khani et al. (2021)	Assessing the feasibility of providing a family skills intervention, ‘Strong Families’, for refugee families in reception centers in Serbia	Pre–post study	25 families, *N* = 50 25 parents, 25 children	Child externalizing (parent)	Strengths and Difficulties Questionnaire	Ser

*Note.* EMDS G-TEP = Eye Movement Desensitisation Reprocessing Group Traumatic Episode Protocol; fu = follow-up; Int. = intervention; LTP = learning through play; RCT = randomized controlled trial; TAU = treatment as usual; WLC = wait list control; RoB = risk of bias, Low = low risk of bias, Mod = moderate risk of bias, Ser = serious risk of bias. ^a^Same original study, reports different outcomes, ^b^Same original study, reports different outcomes, ^c^Follow-up study (Osman, Flacking et al., 2017; Osman, Salari et al., 2017).

#### Combined interventions

Three of the combined studies were RCTs, randomized at family level (Ahktar et al., 2021; Betancourt et al., 2020; Puffer et al., 2017) and one was a pre–post treatment study (El-Khani et al., 2021).

#### Included outcomes: parenting interventions

Of the primary outcomes, positive parenting (Bjorknes & Manger, 2013; Bjorknes et al., 2015; Shaw et al., 2021), negative parenting (Bjorknes & Manger, 2013; Shaw et al., 2021) and parental self-efficacy (Osman, Salari et al., 2017; Shaw et al., 2021) were reported in two RCT studies each. Parent-rated changes in internalizing and externalizing behaviours among children were reported in three randomized controlled studies, two on each domain (internalizing: Bjorknes & Manger, 2013; Dybdahl, 2001; Osman, Flacking et al., 2017; externalizing: Bjorknes & Manger, 2013; Osman, Flacking et al., 2017). Miller and colleagues (2020) also reported on positive and negative parenting outcomes but used a non-validated rating scale, thus these measures were not included. Meta-analyses of these studies were conducted to explore and illustrate tendencies. Additional within-group analyses exploring tendencies for variables included in both RCT and pre–post studies are presented for the following primary outcomes: positive parenting (*n* = 4), negative parenting (*n* = 6), parental self-efficacy (*n* = 3), parent-reported child internalizing (*n* = 3), parent-reported child externalizing behaviours (*n* = 4), self-rated child internalizing symptoms (*n* = 2) and child-rated negative parenting (*n* = 2). For the within-group analysis of negative parenting, two subscales of ‘The conflict tactics scale’ (psychological respectively physical aggression past month) reported by Ballard et al. (2018) were removed as *n* < 10. Regarding the secondary outcomes, the most common secondary outcome was parental distress (reported in four RCTs; Bjorknes et al., 2015; Miller et al., 2020; Osman, Salari et al., 2017; Shaw et al., 2021). No other secondary outcomes were reported in a sufficiently homogenous manner in a sufficient number of RCT studies to allow for between-group synthesis. Within-group tendencies are presented for parental: well-being, psychological distress, anxiety, depression, and trauma-related symptoms.

#### Included outcomes: combined interventions

There were two RCTs with measures on each primary outcome; however, in one study we could not interpret the outcome for between-group effects of parent-rated child externalizing behaviours at post measurement, why this outcome was excluded (Ahktar et al., 2021). Meta-analyses were conducted to explore tendencies in: child-rated depression (internalizing), child-rated externalizing behaviours, parent-rated child internalizing behaviours, parent-rated positive parenting and parent-rated negative parenting. El-Khani and colleagues (2021) was the only non-randomized, combined intervention study. An additional within-group analysis of parent-rated child externalizing behaviours was conducted with this study included. Within-group analyses of negative (*n* = 2) and positive parenting (*n* = 2) were also conducted. No secondary outcomes were reported in two or more studies.

#### Follow-up data

Two studies of parenting interventions, one as a separate study on 3-year follow-up data (Osman et al., 2021) and one in which follow-up data was included together with the post-intervention evaluation (Shaw et al., 2021), a time point of measurement after the post-intervention measurement. However, because both time point and variables differed between the two studies, no meta-analysis was possible. These data are described qualitatively.

### Participant characteristics

The characteristics of the participants are presented in Table 2.

**Table 2a.** Participant characteristics in the included studies.

Author, year	Study population	Ethnicity/country of origin	Current location and time in host country (*M*)	Housing situation	Parent mean age (*SD*) years	Gender (% women)	Level of education: number of years (*M*, *SD*), number of participants (%)	Mean number of children (*SD*)	Age span children (*M*)	Gender (% girls)
Ballard et al. (2018)	Refugees	Karen from Burma	USA *M* = 2.66 (*SD* = 2.21) years	NI	33.50	100	*M* = 5.91 (*SD* = 4.44)	3.91 (2.59)	5–13 (10.45)	33/67 direction unclear
Bjorknes and Manger (2013) Bjorknes et al. (2015)	Refugees or family reunification	Somali	Norway *M* = 8.89 years	‘Living in community’	33.71	100	College/university 7%, High school graduate 38%, Elementary school 55%	4.05	3–9 (5.9)	37
Dybdahl (2001)	Internally displaced	Bosnian	Bosnia and Herzegovina < 4 years displaced	Refugee settlement, private housings	20–44 (30.7)	100	Illiterate 14%, Read a little 14%, Literate 72%	2.4	5–6 (5.5)	55
Eltanamly et al. (2022)	Refugees	73% Syrian	Netherlands *M* = 41 months	NI	NI	69.9	Primary school 15.4%, Vocational school 9.7%, Secondary school 34.0%, University 40.8%	NI	10–15 (12.6)	50.8
Husby et al. (2020)	Refugees	41% Syrian, 2% Palestinian, rest unknown	Denmark < 5 years	NI	NI	64.1	NI	NI	NI	NI
Kaptan et al. (2022)	Refugees or asylum seekers	Multiple nationalities	United Kingdom *M* = 3.8 years	NI	24–49 (33.2)	100	*M* = 10.35 years	NI	< 3	NI
Lakkis et al. (2020)	Refugees	Syria	Lebanon and Jordan	Refugee camps	NI	60.80	NI	NI	In utero to 6 (4)	NI
Morris et al. (2012)	Internally displaced	Acholi	Uganda *M* = 4.2 years in camp	Refugee camps	26.6	100	No schooling 39%, Lower primary 20.2%, Upper primary 40.5%	3.4	6–30 months (14.5)	NI
Miller et al. (2020)	Refugees	Syrian, Palestinian, Lebanese	Lebanon	Apartment 38.4%, House 54%, Tented settlement < 1%, Other 6.5%	NI	50.3% (grandmother/ grandfather: 1.4%)	No schooling 3.3%, Primary 34.3%, Secondary 31.1%, High school 17.2%, Vocational 6%, University 9%	NI	3–12 years (indexed child)	42
Osman, Salari et al. (2017) Osman, Flacking et al. (2017)	Refugees (war or family reunification)	Somalia	Sweden 1–5 years 60.8%, 6–9 years 24.2%, > 10 years 15%	NI	44.5	66.7	< Upper secondary 57.5%, Upper secondary 36.7%, Tertiary 5%	5	11–16 (13.5)	42.5
Osman et al. (2021)			Sweden 1–5 years 39.2%, 6 years or more 60.8%	NI	43.8	66.7	< Upper secondary school 56.9%	5.74	11–16	62.7
Renzaho and Vignjevic(2011)	Primarily refugees	Liberia, Sierra Leone, Congo, and Burundi	Australia < 4 years 82%	‘Homes,	33.4 (10.9)	53.8	Primary or less 49%, Secondary 41%, Tertiary 10%	51% > 3	NI	NI
Shaw (2021)	Refugees or Asylum seekers	Afghani & Rohingya	Malaysia *M* = 3.5 years	‘Homes’	32 (7.95)	92.7 randomized, 100 analysed	No education 45.6%, Primary school 30.4%, High school 20.2%, College 3.7%	3 (1.26)	<18 (NI)	NI
Sim (2020)	Refugees	Syria	Lebanon 2–3 years 43%	Over half in informal tended settlements	31.8 (8.2)	99.60	Some secondary education 52.9%	3.4 (1.50)	2.0–12 (9.8)	51.1

Note: *M* = mean; NI = No information.

**Table 2b.** Participant characteristics in combined interventions.

Author and year	Study population	Ethnicity/country of origin	Current location and time in host country	Housing situation	Parent mean age (*SD*)	Gender parents (% women)	Level of education (%)	Mean number of children (SD)	Age span children (*M*)	Gender (% female)
Akhtar (2021)	Refugees	Syria	Jordan. 4 years 16.95%, 5 years 64.41%, 6 years 13.56%, 7 years 1.69%, >8 years 3.39%	Living in urban districts of Amman	37.31 (6.96)	95	Mothers/Fathers No schooling 3.4/4.2 Primary school 20.3/29.2 Middle school 61/52 High school or higher 15.3/14.6.	4	10–14 (11.73)	44
Betancourt (2020)	Refugees	Bhutan, Nepal	USA. *M* = 4 years	NI	40.97	52	NI	1.98 (Range 1–5)	8–18 (14.35)	53.1
El-Khani (2021)	Refugees	Afghanistan	Serbia. 1–36 months (*M* = 17.15 months)	Reception Centres	33.4	80	NI	3.3 (1.7)	8–15 (10.5)	36
Puffer, 2017	Forcibly displaced families	Burma	Thailand. NI,	In communities	41	83	No schooling 32 Primary school 50 Middle school 12 High school 4 University 1	NI	7–15 (10.4)	51

Note: NI = No information

#### Parenting interventions

Mainly trials included refugees (*n* = 7), refugees and reunification with a family member with refugee status (*n* = 2), refugees and asylum seekers (n = 2) and internally displaced people (*n* = 2). The different interventions included participants from countries around the globe but most common were studies with participants from Syria (*n* = 5) or Somalia (*n* = 4). There was great variation in where the studies were conducted and in all but one trial (Bjorknes & Manger, 2013; Bjorknes et al., 2015) the participants had been in their current location for 5 years or less. Most of the studies did not explicitly describe the housing situation of the participants. The mean age of the parents in the parenting interventions ranged from 26.6 to 44.5 years (*M* = 33.3), four studies did not report the parents’ age. The educational level of the included parents varied considerably between studies (Table 3). However, the sample size was insufficient to allow for a systematic evaluation of the role of parental educational level, and consequently, no specific hypotheses related to this variable were formulated in this study.

**Table 3.** Between-group effect sizes (Hedge’s *g*) for the comparison of the parent intervention and control condition for primary and secondary outcomes.

			95% CI				
Time point/outcome	*k*	*g*-value	LL	UL	*z*-value	*Q*-value	*I*^2^ (%)
Primary outcomes							
Child externalizing behaviour	2	0.43	0.13	0.72	2.82^b^	1.15	13.19
Child internalizing behaviour	2	0.11	−0.17	0.40	0.77	0.05	0.00
Positive parenting	2	0.89	0.17	1.61	2.42^a^	5.21	80.82
Negative parenting	2	0.21	−0.09	0.51	1.39	0.16	0.00
Self-efficacy	2	1.94	1.57	2.31	10.28^c^	1.12	11.06
Secondary outcomes							
Psychological distress (parent)	4	0.67	0.10	1.24	2.32^a^	24.70	87.86

*Note*: k = number of studies; CI = confidence interval; LL = lower limit; UL= upper limit; *z* = test of significance for *g*; *Q* = statistical test of heterogeneity; *I*^2^ = level of heterogeneity; a = *p* < .05, b = *p* < .01, c = *p* < .001.

#### Combined interventions

Three of the combined studies included refugees and one study included forcibly displaced people (Puffer et al., 2017). Families of different nationalities were included in each study. The mean age of the parents ranged from 33.4 to 37.3 years (*M* = 38.17). A more extensive summary of participant characteristics is described in Appendix 3.

### Assessment of risk of bias in studies

#### Parenting interventions

The bias of all RCTs of parenting interventions were assessed as ‘moderate’ in line with the Cochrane Risk of Bias Tool (RoB) (Sterne et al., 2019). The most common reason for this estimate was that the self-rating scales did not allow blind assessment. Further, none of the studies included a data analysis plan in their pre-registered study protocol.

The pre–post studies of parenting interventions were assessed with ROBINS-I (Sterne et al., 2016) and coded similarly to the RCTs. Six were assessed as moderate, and four as high risk. The higher risk of biases was for similar reasons to the RCTs: unblinded outcome assessors and no pre-specified analysis plan. In addition, the studies coded with high risk described no appropriate method to control for confounders.

#### Combined interventions

Of the combined RCTs, three studies were coded as moderate risk of bias, and the reasons mirrored those for parenting interventions. The combined pre–post study was assessed to have high risk of bias because of the inability to control for confounders.

### Effects of parenting interventions on primary outcomes

Statistics for between-group analyses of parenting interventions are presented in Table 3 and forest plots in Appendix 4. Statistics for within-group analyses of parenting interventions are presented in Table 4 and forest plots are found in Appendix 5. For an overview of certainty of evidence assessment see Appendix 7.

**Table 4.** Within-group effect sizes (Hedge’s *g*) for primary and secondary outcomes.

			95% CI				
Time point/outcome	*k*	*g-*value	LL	UL	*z*-value	*Q*-value	*I*^2^ (%)
Parent ratings							
Primary outcomes							
Child externalizing behaviours	4	0.51	0.35	0.68	6.06^c^	2.07	0.00
Child internalizing behaviours	3	0.50	0.27	0.72	4.23^c^	3.50	42.88
Negative parenting	6	0.33	0.06	0.60	2.36^b^	24.85	79.88
Positive parenting	4	0.25	0.08	0.42	2.81^b^	2.95	0.00
Self-efficacy	3	1.26	0.04	2.47	2.03^b^	54.06	96.30
Secondary outcomes (parental)							
Wellbeing	2	1.91	−1.14	4.97	1.23	40.22	97.51
Psychological distress	5	0.62	0.28	0.96	3.55^c^	35.69	88.79
Anxiety	2	0.45	−1.25	2.16	0.52	13.85	92.78
Depression	2	0.53	−1.63	2.7	0.48	18.76	94.67
PTSD	4	0.41	−0.05	0.87	1.76	18.10	83.43
Child ratings							
Primary outcomes							
Child internalizing behaviours	3	0.42	−0.01	0.86	1.90	9.54	79.03
Negative parenting	2	0.13	−0.54	0.79	0.37	4.53	77.92

*Note*: *k* = number of studies; CI = confidence interval; LL = lower limit; UL = upper limit; *z* = test of significance for *d*; *Q* = statistical test of heterogeneity; *I*^2^ = level of heterogeneity; a = *p* < .05, b = *p* < .01, c = *p* < .001.

#### Between-group results

The included studies displayed a considerable degree of variation regarding interventions, age of children, length or intervention and intervention setting. All comparators were wait list control groups, except Dybdahl (2001) in which the control group received treatment as usual.

We found significant reductions in parent-rated child externalizing behaviour (⨁⨁⨁◯), but no significant reductions in internalizing behaviours (⨁⨁◯◯). Furthermore, we found significant increases in self-reported use of positive parenting strategies (⨁◯◯◯) in intervention groups compared with control conditions, but no significant reduction in negative parenting (harsh and inconsistent parenting, or poor supervision) (⨁⨁◯◯). Study variations were observed in the psychometric measures used, implementation contexts, intervention components and dose, participant ethnicities and child characteristics. These variations may help explain heterogeneity. In analyses of parental self-efficacy, a significant increase was observed (⨁⨁⨁◯). No outliers and no publication bias were detected, but results must be interpreted with caution owing to the small number of studies.

#### Within-group results

All within-group analyses are presented in Table 4. Within-group comparisons of parent-rated child internalizing and externalizing symptoms were possible for four studies each. Heterogeneity was low, and both analyses indicated symptom reduction from pre to post assessments in intervention groups: externalizing (*g* = 0.51, 95% CI [0.35, 0.68, *z* = 6.06, *p* = .01, *Q* = 2.07, *I*^2^ = 0.00), internalizing (*g* = 0.50, 95% CI [0.27, 0.72, *z* = 4.23, *p* = .001, *Q* = 3.50, *I*^2^ = 42.88). Analysis of self-reported child internalizing behaviours indicated no significant changes. No outliers and no publication bias were detected in the within-group analyses.

A significant increase in positive parenting strategies was observed at post measurements in intervention groups (*g* = 0.25, 95% CI [0.08, 0.42], *z* = 2.81, *p* = .01, *Q* = 2.95, *I*^2^ = 0.00).

Negative parenting was reduced from pre to post measurements for parents taking part in intervention groups (*g* = 0.33, 95% CI [0.06, 0.60], *z* = 2.36, *p* = .02, *Q* = 24.85, *I*^2^ = 79.88). Analysis of child-reported negative parenting indicated no significant change. In addition, significant increases of parental self-efficacy were found (*g* = 1.43, 95% CI [0.51, 2.34], *z* = 3.053, *p* = .01, *Q* = 28.812, *I*^2^ = 93.06). However, heterogeneity was very high. As in previous analyses, this may be explained by the considerable between study variation in psychometric measures used, implementation contexts, intervention components and dose, participant ethnicities and child characteristics.

### Effects of parenting interventions on secondary outcomes

#### Between-group results

Parent-rated psychological distress was reported in four RCTs, all with a moderate risk of bias (Table 3). In the study by Miller and colleagues (2020), the intervention was offered to all refugees (universal prevention), whereas in the remaining studies, the interventions were offered to refugees and asylum seekers and at the selected level of prevention. The analysis indicated that parental distress reduced significantly more for parents in intervention groups than control groups (⨁◯◯◯). The high levels of heterogeneity could be explained by variation in psychometric measures used, participant ethnicities, implementation settings (Scandinavia, Malaysia and Lebanon) and intervention content and dose.

#### Within-group results

In addition to the four RCT studies, self-rated psychological distress among the parents was also measured in two pre–post studies (Morris et al., 2012; Sim et al., 2020). However, one study (Morris et al., 2012) could not be included in the analysis because it did not use a validated measure. The analysis indicated significant reductions in psychological distress post intervention (*g* = 0.62, 95% CI [0.28, 0.96], *z* = 3.55, *p* = .01, *Q* = 35.69, *I*^2^ = 88.79). One large residual (> ±2) was observed (Bjorknes & Manger, 2013). Because this may have explained some heterogeneity, the study was removed, and a sensitivity analysis performed. Subsequently, another large residual was detected (Shaw et al., 2021). This study was also removed from the analysis, and no further problems were detected. The results of the sensitivity analysis remained positive and significant with low heterogeneity (*g* = 0.69, 95% CI [0.59, 0.8], *z* = 12.83, *p* = .01, *Q* = 21.13, *I*^2^ = 0.00) and no publication bias was detected. In the analyses of parental anxiety and parental depression (Ballard et al., 2018; Kaptan et al., 2022) no significant changes between pre and post measurements were observed: anxiety (*g* = 0.45, 95% CI [−1.25, 2.16], *z* = 0.52, *p* = .602, *Q* = 13.85, *I*^2^ = 92.78); depression (*g* = 0.53, 95% CI [−0.16, 2.7], *z* = 0.48, *p* = .63, *Q* = 18.76, *I*^2^ = 94.67). Heterogeneity was high in both analyses, which may be explained by variation in the psychometric measures used, intervention components and modalities, and participant ethnicities. Similarly, no significant changes after intervention were observed in analyses of well-being (Husby et al., 2020; Miller et al., 2020) or PTSD (Ballard et al., 2018; Dybdahl, 2001; Kaptan et al., 2022; Sim et al., 2020). One large residual was detected in the analysis of PTSD, whereby the study was removed. A sensitivity analysis indicated a significant reduction in symptoms of parental PTSD post interventions (*g* = 0.62, 95% CI [0.32, 0.91], *z* = 4.14, *p* = .01, *Q* = 4.955, *I*^2^ = 59.64) and no publication bias was detected. Child well-being was measured in two studies (Lakkis et al., 2020; Miller et al., 2020) but rated by parents in the first study and by children in the second. All within-group analyses are presented in Table 4.

### Effects of combined studies on primary outcomes

Statistics for between-group analyses of combined interventions are presented in Table 5 and forest plots are presented in Appendix 6. For an overview of certainty of evidence assessment see Appendix 8.

**Table 5.** Between-group effect sizes (Hedge’s g) for the comparison of Combined intervention and control condition for primary outcomes.

			95% CI				
Time point/outcome	*k*	*g*-value	LL	UL	*z*-value	*Q*-value	*I*^2^ (%)
Post							
Parent rated							
Child internalizing behaviour	2	0.14	−0.73	1.00	0.31	5.14	80.53
Positive parenting	2	0.17	0.00	.36	1.99^a^	0.14	0.00
Negative parenting	2	0.19	−0.16	0.54	1.08	1.95	48.69
Child rated							
Depression	2	0.06	−0.32	0.43	0.29	0.05	0.00
Externalizing behaviours	2	−0.08	−0.87	0.71	−0.20	4.25	76.45

*Note*: k = number of studies; CI = confidence interval; LL = lower limit; UL = upper limit; *z* = test of significance for *g*; *Q* = statistical test of heterogeneity; *I*^2^ = level of heterogeneity; a = *p* < .05, b = *p* < .01, c = *p* < .001.

#### Between-group results

Four studies of combined family interventions (including both parent and child sessions, or family sessions) were included. Three studies were RCTs but no more than two studies reported on each outcome, meaning robust meta-analyses were not possible. Exploratory analyses indicated significant effect of positive parenting but on no other primary outcome (Table 6). Please see Appendix 6 for forest plots. Puffer et al. (2017) was the only study to include child reports on parenting behaviours. Child well-being and parental distress were measured in one combined study (Ahktar et al., 2021). Too few studies reported secondary outcomes to conduct an analysis. No publication bias or outliers were detected.

**Table 6a.** Parent intervention characteristics.

Author and year	Level of prevention	Intervention	Classification	Content	Cultural tailoring	Group size	Length	Language	Leader	Setting
Ballard (2018)	Universal/selected. Offered to all Karen refugees	Enhancing Family Connection Generation Parent Management Training – Oregon Model	Social learning theory	Starting point from parent's own values and descriptions. Use of genograms, role-play and case examples, discussions and homework, psycho-education, societal information and parent skills training (PMTO)	Extensive cultural adaptation based on focus group interviews with the local community. Karen metaphors and symbols, adaptation of implementation modality and specific material regarding the Karen culture	5–6	9 weeks, 9 sessions 15 h	English and translator (Karen)	Trained providers (author, translator, family therapist)	School
Bjorknes and Manger (2013) Bjorknes et al. (2015)	Selected. A child with or at risk of developing conduct problems	Parent Management Training – Oregon Model	Social learning theory	Training of positive parenting skills: positive involvement, effective discipline, problem-solving, monitoring, reinforcement. Through therapeutic activities, dyadic teaching, group process suggestions, role-play, modelling and home practice assignments	Culturally adapted vignettes, gender segregated groups, a manual designed to implement PMTO among ethnic minorities, ethnically homogenous groups, and use of link-workers during sessions	8–12	18 weeks, 18 sessions, 36 h	Norwegian, Somali-speaking bilingual worker	2 psychologists/ social workers trained in PMTO Bilingual link worker	Community care centres
Dybdahl (2001)	Selected	Early childhood care and education programmes	Developmental psychology, trauma-informed therapy	Semi-structured meetings, psycho-education about trauma, reinforcement of positive coping strategies, strengthening parental self-efficacy and developmental education, group discussions with own experiences and suggestions posed by leaders + 1 h home visit	Therapeutic discussion groups for traumatized women held during the Bosnian war. Intervention was chosen because it was easily culturally adapted	5	Weekly 5 months	NI	Trained preschool teachers	Refugee settlement
Eltanamly (2022)	Universal/selected. Offered to all Arabic-speaking refugee parents	A single session parenting intervention	Social learning theory	An individual feedback intervention to reinforce positive parenting practices and thereby strengthen parental self-efficacy. A standardized structure for personal feedback. Different components of self-efficacy targeted: mastery experience based on a true story, verbal persuasion, positive social comparison	A person-centred intervention	1	One day	NI	Specialist in child development and education or trained research assistant	Home visit
Husby (2020)	Universal/Selected. Offered to all refugees	MindSpring programme	Psycho-education, social learning theory	Psycho-education and psychosocial themes, group discussions, group tasks/exercises, role-play, drawing and relaxation exercises. Self-reflection and awareness training, communication skills, reinforcement, peer-to-peer, inclusion of participants own experiences, self-agency, empowerment, parenting and acculturation, concrete parenting strategies. Parent specific social and cultural information about Denmark	Developed for the targeted group, contains information about being and parenting in a new culture, and cultural differences with focus on the Danish context	8–10	9 weeks, 9 sessions, 18 h	Danish, Arabic translation	Social worker, MindSpring trainer (volunteer refugee/immigrant with same background as group) translator	Local areas, community locations, social housing projects
Kaptan (2022)	Universal/Selected. Offered to refugees or asylum seekers in the UK who are registered with a general practitioner	Learning Through Play (LPT), Eye Movement Desensitization Reprocessing, Group Traumatic Episode Protocol (EMDR G-TEP)	CBT, EMDR psycho-education	Online psycho-education and CBT. LTP aims to stimulate parent–child attachment by increasing parental participation and teaching parents how to use play activities to enhance the development of their children with a pictorial calendar. G-TEP is a group intervention that aims to reduce the impact of recent traumatic experiences and process its ongoing consequences. The protocol works on the principle of EMDR therapy and uses a structured worksheet with a focus on present safety and positive future templates	LTP was culturally adapted. EMDR G-TEP is culture-friendly because of the possibilities for confidential expression through drawings as well as writing	6–8	8 weeks, 8 sessions, 8–12 h	English	2 facilitators (one active, one observer to check for signs of distress in participants)	Zoom
Lakkis (2020)	Selected	New programme	Psycho-education developmentally informed education	6 early development sessions, 5 mental health sessions (part of the project ‘SANAD’). Brainstorming activities, group work, role-play, case study, short presentations and open focus groups discussions. Focus on early child development and psycho-education, communication skills and in general reinforcement of positive behaviour	NI but a new intervention developed in Arabic	NI	21 weeks, 21 sessions, 42–63 h	Arabic	Psychologists experienced in parent training and coaching (one man and one woman in each group)	Refugee encampments
Morris (2012)	Selected. Malnourished parents	Infant stimulation with emergency feeding	Developmentally informed psycho-education	Psycho-education about key areas of child development, maintenance skills, skills training, individual counselling (home sessions). Psycho-education (group sessions) adapted from the ‘Learning through play program’. Picture-based material on the importance of play, and the five key areas of child development (physical, intellectual, language and communication, relationships, and sense of self)	Adapted using findings from a focus ethnographic survey and drawings by a local artist to reflect details of the Acholi internally displaced populations culture and context. Primarily picture-based materials that can be used to educate mothers with no or little reading skills	*M* = 16 (range 7–25)	6 weeks, 6 sessions and 0–3 home visits (*M* = 1.32), 12 + 1–2 h	Acholi	Trained psychosocial facilitators	Home visits and established feeding centres
Miller (2020)	Universal/Selected. Offered to all refugees	Caregiver support intervention (CSI)	Social learning theory and third-wave CBT	Psycho-education about stress, positive parenting practices, emotion regulation skills, mindfulness (relaxation skills). Discussions, problem-solving, homework	Culturally sensitive newly developed programme. Developed together with stakeholders and then piloted with participants in Gaza. Focus groups with pilot participants. Use of locally salient idioms in the intervention	12	9 weeks, 9 sessions, 13.5 h	NI	2 trained non-mental health specialists	Three community-based organizations
Osman, Salari et al. (2017) Osman, Flacking et al. (2017) Osman et al. (2021)	Selected. Parents have self-perceived stress	Connect and culturally tailored societal information	Attachment based, CBT, mindfulness-based therapy	Role-plays, reflections, structured discussions, psycho-education (attachment and trauma) and handouts. Therapeutic techniques used as needed, refection exercises, emotional regulation skills, conflict management, mutuality with child, increased parental sensitivity (interpret behaviour though and attachment lens)	2 sessions of societal information developed from focus group interviews with the target group and a culturally adapted Connect (changes to role-plays, examples and metaphones to make them understandable without changing core components)	12–17	12 weeks, 12 sessions, 12–14 h	Somali	Leaders with Somali background (1 male/1 female per group)	In a local community setting
Renzaho (2011)	Selected. Parents already used a counselling program for parenting issues	The African Migrant Parenting Program	Education and skills training	Sessions with the aim to understand child development, help the child to develop self-confidence (e.g., realistic and developmentally appropriate expectations), improve communication, strengthening family relations through communication of feelings, addressing legal issues and how to deal with it in the family, managing family stress and addressing parenting in a new culture (e.g., personal power/negative control, managing anger, making choices). This through presentations, small-group discussions, scenarios, and mini-case studies. Home visits to monitor barriers and to practice skills learnt from the sessions	Nothing specific. Modelled on parent training skills from ‘Parenting in a new culture developed for Australian–Samoan parents’ developed by Spectrum Migrant Resource Centre	8–10	8 weeks, 8 sessions, 16 h + 3 × 45 min home visits	NI	Qualified African parenting educators and external experts with background in psychology, family or relation counselling	A migrant resource centre
Shaw et al. (2021)	Selected	No name 8-week parenting programme	Social learning theory and social ecological framework	Strengthening parental self-efficacy, increase positive parenting practices, supporting family functioning (content on communication skills, conflict and meeting) and enhance parental well-being. Contents on child development along with strategies to manage child behaviour to enhance parental self-efficacy, reduce child intensity and increase positive parenting strategies. Components: check-in, psycho-education, discussion, role-play	Content developed from existing programmes used in a Malaysian context and focus groups with representatives from the Afghan and Rohingya refugee communities. Together with existing knowledge on culturally tailored CBT	*M* = 9	8 weeks, 8 sessions, 8 h	NI	Para-professional researcher from respective refugee communities with experience of service provision and interpretation	In the community
Sim et al. (2020)	Selected	Families make the difference intervention	Based on biopsycho-social theory, aims to strengthen protective factors and components from trauma-focused CBT	Discussion, psycho-education, and skills training groups based on Parents Make the Difference and Teaching Recovery Techniques	Adapted from a previous programme (IRC and TRT). Adaptations not described in detail but were made in response to previously collected data regarding high levels of parental stress and child abuse and neglect, and child psychosocial problem in a neighbouring population	15–20	10 sessions, NI	NI	Trained local IRC staff and refugee volunteers	Community centres or individual lends in tented settlements

*Note.* NI = no Information, CBT = cognitive behavioural therapy. Classification = not described in all studies and based on the authors interpretation of the content.

**Table 6b.** Combined intervention characteristics.

Author and year	Level of prevention	Intervention	Classification	Content	Cultural tailoring	Group size	Length	Language	Leader	Setting
Akhtar(2021)	Selected	Early Adolescent Skills for Emotions (EASE)	CBT with a focus on emotion regulation	Psycho-education and skills training. Child sessions: psycho-education, problem-solving, stress management (diaphragmatic breathing), behavioural activation and relapse prevention. Caregiver sessions: psycho-education, active listening, quality time, praise, self-care and relapse prevention	EASE was initially adapted for Syrians residing in Lebanon and further adapted for use in Jordan. Following adaptation, focus groups were conducted with stakeholders and EASE facilitators to ensure the manual was contextually appropriate for Syrian refugees	5–8 children and their parents	7 weeks. Children: 7 sessions (10.5 h) Parents: 3 sessions (6 h)	Arabic	2 trained facilitators with a variety of relevant backgrounds. 8 days training and weekly supervision	NI. Control group at homes
Betancourt (2020)	Selected	Family Strengthening Intervention for refugees	Narrative therapy	Family visits with discussion, societal information and building a family narrative. A strengths-based intervention with core components including a family narrative that draws out family challenges, strengths and collective future hopes that can be achieved through improved communication	Extensive qualitative work, including free list interviews, focus groups and key informant interviews who assessed the needs, strengths and challenges of each community to adapt the intervention accordingly	Whole families	10 weeks, 10 sessions, 15 h	Arabic	Trained members of the refugee community who were provided supervision from a social worker	Home visits
El-Khani (2021)	Selected	Strong Families	Family skills programme for challenged settings	Child sessions: Focus on stress, activities regarding rules and responsibilities, think about future goals and the roles the caregivers play in their lives. Through discussions, mapping existing skills and competencies, identify challenges, skills and communication training. Parent sessions: Deal with stress; develop positive parenting strategies; improve communication skills, decrease coercive parenting. Family sessions: Practice positive communication, stress relief techniques, learn about family values and practice sharing appreciation to each other	The programme has previously been culturally tailored and tested in Afghanistan	Max. 7 families. Child and primary caregiver	3 weeks, 2 child sessions (4 h), 3 parent sessions (5 h) and 2 combined	Interpreters taking part in training	Trained facilitators with mixed background, who had access to caregivers and their children in the 3 study settings	Reception centres
Puffer (2017)	Selected	Happy Families /Strengthening Families	Based on biopsycho-social theory, aims to strengthen protective factors	Psycho-education (child development, drugs and alcohol), skills training (manage stress, goal setting, reinforcement + shaping, communication skills, problem-solving, setting limits, behaviour change) maintaining change. Parallel sessions (caregiver + children) activities and a meal together at each session. Goal to achieve acquisition of both knowledge and skills	Qualitative research to supplement the material with culturally and religiously relevant concepts. Inclusion of traditional Burmese stories and relevant examples of displacement. Concepts were simplified and handouts eliminated due to low literacy rates among participants	NI (8–12 families described as ideal)	12 weeks, 12 sessions, 30 h	NI	IRC trained staff member and one trained leader from local Burmese community	In the community

*Note. NI* *=* No Information, *CBT* = Cognitive Behavioural Therapy. *Classification* = not described in all studies and based on the authors interpretation of the content. IRC = International Rescue Committee.

#### Within-group results

Only one pre–post study of a combined intervention was included in this meta-analysis (El-Khani et al., 2021), which reported on child externalizing behaviours. Although this outcome was included in two RCT studies of combined programmes (Ahktar et al., 2021; Betancourt, 2020), within-group pre–post data was not presented by Betancourt and colleagues (2020). Attempts to retrieve additional data were not successful. Thus, within-group analysis of parent-reported child externalizing behaviours were conducted for Ahktar et al. (2021) and El-Khani et al. (2021). No significant changes in parent-rated externalizing behaviours were observed (*g* = 0.13, 95% CI [−0.16, 0.43], *z* = 0.88, *p* = .38, *Q* = 1.33, *I*^2^ = 24.51). No significant changes between pre and post measurements were observed regarding parenting practices; positive parenting (*g* = 0.15, 95% CI [−0.34, 0.65], *z* = 0.61, *p* = .543, *Q* = 6.94, *I*^2^ = 85.58) and negative parenting (*g* = 0.32, 95% CI [−0.18, 0.82], *z* = 1.24, *p* = .214, *Q* = 7.556, *I*^2^ = 86.77).

### Follow-up data for parent and combined interventions

Two RCT parent intervention studies (Osman et al., 2021; Shaw et al., 2021) and one pre–post combined intervention study (El-Khani et al., 2021) reported follow-up data. Osman and colleagues (2021) published a 3-year follow-up (*n* = 51), indicating sustained positive effects on child internalizing and externalizing behaviours and parental distress. Shaw et al. (2021) reported 3-month follow-up data on parental distress, self-efficacy and positive and negative parenting. Post-intervention improvements were maintained for all outcomes except parental distress at the 3-month follow-up. El-Khani et al. (2021) reported a 6-week follow-up of parent-reported child externalizing symptoms. Conduct problems were improved at post intervention and further improvements observed at follow-up (*p* = .01).

### Intervention characteristics

A summary of intervention characteristics is provided in Table 6. Despite the range of interventions included, several similar features in programme components, format and delivery were identified. However, it was not possible to examine possible moderators and predictors (e.g. treatment components, cultural tailoring) owing to the small number of studies included in the analyses. Some of the programmes included in our study have to varying extent also been evaluated in other populations than among forced migrants.

The only interventions to reoccur in the included studies were Connect for Somali Parents in Sweden (Osman, Flacking et al., 2017; Osman, Salari et al., 2017; Osman et al., 2021) and Parent Management Training – Oregon Model in Norway (Bjorknes & Manger, 2013; Bjorknes et al., 2015). Six of the parenting interventions were assessed (by the review authors) to have been delivered at a universal/selected level and seven at a selected level, whereas all the combined interventions were at a selected level. All but two interventions were implemented in groups; Eltanamly (2022) investigated the effects of an individually tailored parenting feedback intervention, and Betancourt (2020) the effects of family visits. Common components among all interventions were: psycho-education, group discussions and skills training.

Most parenting interventions were based on social learning theory, but several other conceptual underpinnings were described, including developmental psychology, cognitive behavioural therapy (CBT), third-wave CBT, eye movement desensitization therapy, positive feedback and attachment theory. The combined interventions were based on CBT, narrative therapy, general skills training and skills training with a focus on strengthening protective factors.

A strategy for cultural adaptation was described for most interventions. These ranged from offering the programme in mother tongue and using culturally appropriate expressions and metaphors, to extensive and thorough adaptations, or newly developed programmes specifically targeting the cultural context. Most interventions were implemented in the participants’ mother tongue (either in their entirety or through interpreters or bilingual leaders).

## Discussion

The aim of this meta-analysis was to systematically evaluate available empirical knowledge regarding the efficacy of preventive interventions targeting forced migrant parents. A range of outcomes was evaluated, including as primary outcomes: child internalizing and externalizing behaviours, parental competence (positive/negative parenting), and parental self-efficacy; and as secondary outcomes: parental well-being and mental health/psychological distress. Twenty publications were included, of which there were 13 parent trials (16 publications) and 4 combined trials (4 publications). The between-group analyses indicated improvements in parent-rated child externalizing behaviours, positive parenting strategies and parental self-efficacy. Further, within-group analyses also indicated significant improvements. Analyses of the combined interventions indicated a small significant increase in positive parenting strategies.

In accordance with evaluations of general parenting programmes on universal (Stattin & Enebrink, 2020) and selected levels (Leijten et al., 2019), our analyses suggest that parenting interventions could be effective in promoting psychological health and positive parenting practices among forced migrants. However, more RCT studies would be necessary to perform robust evaluations of effectiveness and to explore possible moderators and predictors of effect. Although it may be assumed that factors such as intervention modality, refugee status, intervention setting, participants’ level of education and cultural tailoring could influence effects, there is currently too little data available to conduct such analyses. There is also a need for studies to include more follow-up data because it is currently unclear whether intervention effects are sustained. The lack of long-term follow-up is understandable in studies of forced migrants, because their living conditions can change quickly. However, the retention of positive intervention effects indicated in one 3-year follow-up study (Osman et al., 2021) is promising, and suggests that follow-up studies may be viable with forced migrant populations, despite the challenges.

Despite heterogeneity in the included studies (e.g. differing interventions), there were also similarities, especially in the intervention components (e.g. psycho-education and skills training promoting positive parenting strategies). Today, we have limited knowledge about the processes of change that occur in parent support programmes in general. This knowledge is based on only a few studies that investigated the relationship between change and intervention components in a parenting programme for disruptive child behaviour (Leijten et al., 2019, 2021). These studies found that the components most strongly associated with effectiveness in preventive programmes were behaviour management techniques, and behaviour management together with parental self-management (Leijten et al., 2019, 2021). These components are present to various extents in the programmes included in this meta-analysis. It has also previously been seen that up to 45% of the effects of the parenting programmes on child and adolescent externalizing problems were related to changes in parenting practices (Forehand et al., 2014). Increased evidence regarding the effective components of programmes could aid development of globally effective parenting programmes (Leijten et al., 2021). Moreover, focus on mechanisms of change may decrease focus on branding and marketing of individual programmes (it was noted that there were no intervention replications in the samples included in this review) and instead promote rigorous programme testing.

Interestingly, some of the studies in this meta-analysis had a larger proportion of male parent participants than is usually observed in studies of parenting interventions (Panter-Brick et al., 2014; Sarkadi et al., 2008; Wells et al., 2015). Although the reason for this is unclear, it may be understood in the context of changing roles experienced during displacement and resettlement, where fathers may be less likely to work (Eurostat, 2019) and/or become more involved in their families (Wali & Renzaho, 2018). In future studies of parenting interventions for forced migrant families, it would be of interest to evaluate the predictive effects of whether only one or both parents participate.

Cultural tailoring has previously been identified as an important element of promotive interventions for diverse populations. For example, cultural sensitivity and use of one's own language has increased levels of participation among minority and migrant populations (Hamari et al., 2022). Almost all interventions included in this study reported cultural adaptations to various extents, or programme tailoring to better engage the target group. Future research may be improved by systematic reporting of cultural adaptations and tailoring to enable analyses of the extent of cultural tailoring and its relation to programme effectiveness.

Development of procedures to reduce risk of bias in research with underserved populations would considerably increase the quality of the evidence in the field. Only three studies included in this analysis had blinding procedures (Ahktar et al., 2021; Betancourt et al., 2020; Eltanamly et al., 2022). Eltanamly and colleagues (2022) evaluated a one-session individual feedback intervention in which participants to some extent were blinded. Similarly, Ahktar and colleagues (2021) blinded the assessors in their study evaluating a CBT intervention targeting emotion regulation among youth and parents. The other studies were assessed to have higher risk of bias because of aspects such as unblinded assessors, no pre-specified analysis plan and no appropriate method to control for confounders. There are admittedly considerable challenges in conducting research with underserved populations (Schenker et al., 2014); some procedures can thus be more difficult to implement (e.g. blinded assessors). However, procedures that are more easily implemented could still be used (e.g. study protocol and a pre-specified analysis plan). An increase in high-quality studies would allow more robust comparisons of the results of these kinds of interventions, allowing a greater understanding of effects and their predictors.

### Strengths, limitations and future research

To our knowledge, this is the first meta-analysis including universal and selective parenting interventions for forced migrant families. A methodological strength in this study is that we chose to retain strict inclusion criteria regarding the population (it was necessary for the study sample to have been specified as forced migrant study) to reduce heterogeneity and maintain stringency. However, this may also be regarded as a limitation, because our own definition of forced migrant may be considered arbitrary. In line with Mattelin and colleagues (2022), a major difficulty in our inclusion process was vague definition of the target group and description of the included sample. An overwhelming number of intervention studies were excluded because they included migrants with a variety of backgrounds (e.g. forced, labour, arranged marriage) and second-generation immigrants. For example we excluded many studies conducted in the United States with Latin American migrants. Although it could be argued that many members of this population fulfil criteria to be recognized as refugees, no separate group analyses were performed, meaning it was impossible to ascertain results for forced migrants alone. Overall, antecedents to migration were very rarely specified, which could reflect the reality where there are seldom contexts in which people have the same refugee status. Although a pragmatic approach when conducting interventions and research with forced migrant populations needs to be prevailing, describing the sample in intervention studies of forced migrant populations is needed to be able to draw firm conclusions on how best to support different migrant populations.

Moreover, we expected heterogeneity to persist even within the narrowly defined ‘forced migrant’ population, because experiences within this population may vary greatly. For example, regarding being a quota refugee or a non-quota refugee (Duggal et al., 2020), migration route and violence (Arsenijević et al., 2017), family separation (Liddell et al., 2021), experiences of potentially traumatic events (Sigvardsdotter et al., 2016), the characteristics of the receiving context and associated post-migration stressors (Hou et al., 2020), such as legal status (Delilovic et al., 2023), daily hassles (Elsayad et al., 2019), cultural distance and other factors influencing their overall experience. Thus, forced migrants are a heterogeneous group, and should be addressed as such in research and societies. Preferably, the migration stressors relevant in each specific case should be addressed to improve the mental health of forced migrants.

Although it is a strength that our study included only validated measures, it is a limitation that we used a broad inclusion criterion for validated measures, rather than exclusively including measures validated for forced migrant/culturally diverse populations. Thus, the measures may not fully capture the constructs they are intended to. As such, our ability to evaluate the effectiveness of the interventions appropriately is diminished. We agree that rating scales and their cut-off points should be adapted and validated for the specific target group, and the use of appropriately validated measures should be an unyielding standard for intervention studies. However, the purpose of this meta-analysis was to take a pragmatic approach and examine the efficacy of current parenting programmes being implemented for refugee families globally based on the existing published data*.*

Another limitation of this study is that the certainty of evidence for all outcomes assessed using the GRADE system were concluded to be low or very low (except moderate on two outcomes), which may be considered rather disappointing. However, the very small number of studies and small sample sizes may partially explain these results. Moreover, several of the included studies were assessed to have moderate risk of bias, and when this is considered together with heterogeneity in results between studies and statistical heterogeneity in the meta-analyses, low or very low certainty of evidence is somewhat expected. Thus, although low to very low certainty of evidence may be problematic in the establishment of prevention recommendations, these results must be viewed in the context of a challenging research environment. Although certainty is low, there are promising effects for several outcomes that warrant further investigation. Moreover, within-group stability rather than change might also be interpreted as a strength. In several RCT studies in which we conducted within-group analysis, the control groups showed larger increases of negative outcomes, suggesting that the intervention may have buffered against the effects of a hostile environment.

Future research on prevention interventions for forced migrant families, should also to a larger extent, use outcomes such as well-being, resilience and quality of life. This would allow assessment of the presence of mental health, and not only the absence of symptoms, as is often the case today. For example, only 4 of the 20 studies in this review included well-being as an outcome, none included resilience or quality of life. This is in apparent opposition to SDG 3, regarding health equity, in which mental health is considered an integral component of health. Indeed, it is accepted that there is no health without mental health, emphasising the continuing need to invest more in promoting mental health and preventing ill-health (World Health Organization [WHO], 2022). Despite this, mental health expenditures represent only a small portion of total health spending in many countries today (Rajkumar et al., 2022), and only a fraction of these funds have been invested in mental health promotion or preventive activities (WHO, 2005). We know that forcibly displaced families are in vulnerable situations, balancing parenthood in parallel, and increased worldwide implementation of parenting programmes could be a step towards reaching the SDGs.

Owing to the small number of studies included, the meta-analyses presented in this study are not robust. Further, we were not able to perform subgroup analyses or analyses of follow-up data and none of the included interventions were replicated within the sample. Although increased research attention is an important advancement, there remain few interventions tailored for, or evaluated in populations of refugees, asylum seekers and internally displaced people (Uphoff et al., 2020). Moreover, there is a desperate need for further testing of existing interventions. There was also considerable variation in intervention content, theoretical underpinnings, cultural tailoring and implementation modalities. This contributes to heterogeneity and limits conclusions that can be drawn. Subsequently, this meta-analysis may be considered exploratory, mapping current interventions and effects.

Despite disappointingly few studies, the interventions that were identified provide a hopeful picture of how preventive parenting interventions can contribute to improved health and well-being among forced migrant families around the world. Most of the interventions had been implemented and evaluated the past 2 to 3 years, indicating increased engagement within this field of research.

## Conclusions

In the past decade, the number of published studies of parenting programmes targeting refugees, asylum seekers and internally displaced families have increased, despite the challenges of conducting interventions in these contexts. This is a promising development. However, more studies, and especially RCTs, are needed to allow for robust inferences about efficacy, as well as potential moderators and predictors of outcome. Such knowledge could reveal the effective components of parenting programmes, and in turn aid the development and implementation of more targeted, brief and effective interventions. The existing parenting interventions for forced migrant parents included in this study appear promising in in their ability to reduce children's externalizing behaviours and general distress among parents, as well as in enhancing positive parenting strategies and parenting self-efficacy. Preventive parenting programmes for forced migrant parents are probably an underutilized resource. Although more high-quality studies are needed, our review suggests that these programmes can be used to promote mental health and positive development for families in vulnerable situations

## Supplemental Material

sj-docx-1-tps-10.1177_13634615251372854 - Supplemental material for The efficacy of parenting interventions for forced migrant families on child internalizing and externalizing symptoms, parental self-efficacy, and parental competence: A systematic review and meta-analysis Supplemental material, sj-docx-1-tps-10.1177_13634615251372854 for The efficacy of parenting interventions for forced migrant families on child internalizing and externalizing symptoms, parental self-efficacy, and parental competence: A systematic review and meta-analysis by Maja Västhagen, Clover Jack Giles, Anna-Clara Hollander, Ata Ghaderi, Livia Van Leuven, Anna Edenius and Pia Enebrink in Transcultural Psychiatry
