# Supplementary material for: The efficacy of parenting interventions for forced migrant families on child internalizing and externalizing symptoms, parental self-efficacy, and parental competence: A systematic review and meta-analysis
Source: Transcult Psychiatry. 2026 Mar 25;63(2):143–68. doi: 10.1177/13634615251372854 (PMC13191069; doi:10.1177/13634615251372854)
Supplement: sj-docx-1-tps-10.1177_13634615251372854 - Supplemental material for The efficacy of parenting interventions for forced migrant families on child internalizing and externalizing symptoms, parental self-efficacy, and parental competence: A systematic review and meta-analysis [file sj-docx-1-tps-10.1177_13634615251372854.docx]

Appendices

[Appendix 1: PRISMA 2020 Checklist 2](#_Toc136340031)

[Appendix 2: Documentation of search strategy 5](#_Toc136340032)

[Appendix 3: Participant characteristics 18](#_Toc136340041)

[Appendix 4: Reason for exclusion 20](#_Toc136340042)

[Appendix 5: Forest plots of between group analysis of primary and secondary outcomes…………….....](#_Toc136340042)..45

[Appendix 6: Forest plots of within-group analysis of primary and secondary outcomes of parent interventions 46](#_Toc136340043)

[Appendix 7: Forest plots of combined interventions 49](#_Toc136340068)

[Appendix 8: Certainty in evidence using GRADE – parenting interventions 50](#_Toc136340069)

[Appendix 9: Certainty in evidence using GRADE – combined interventions 51](#_Toc136340070)

**Appendix 1: PRISMA 2020 Checklist**

| **Section and Topic** | **Item #** | **Checklist item** | **Location where item is reported** |
| --- | --- | --- | --- |
| **TITLE** | | |  |
| Title | 1 | Identify the report as a systematic review. | p. 1 |
| **ABSTRACT** | | |  |
| Abstract | 2 | See the PRISMA 2020 for Abstracts checklist. | p. 2 |
| **INTRODUCTION** | | |  |
| Rationale | 3 | Describe the rationale for the review in the context of existing knowledge. | p. 4 |
| Objectives | 4 | Provide an explicit statement of the objective(s) or question(s) the review addresses. | p. 5 |
| **METHODS** | | |  |
| Eligibility criteria | 5 | Specify the inclusion and exclusion criteria for the review and how studies were grouped for the syntheses. | p. 5-6 |
| Information sources | 6 | Specify all databases, registers, websites, organisations, reference lists and other sources searched or consulted to identify studies. Specify the date when each source was last searched or consulted. | p. 6-7 Appendix 2 |
| Search strategy | 7 | Present the full search strategies for all databases, registers and websites, including any filters and limits used. | Apendix 2 |
| Selection process | 8 | Specify the methods used to decide whether a study met the inclusion criteria of the review, including how many reviewers screened each record and each report retrieved, whether they worked independently, and if applicable, details of automation tools used in the process. | p. 7 |
| Data collection process | 9 | Specify the methods used to collect data from reports, including how many reviewers collected data from each report, whether they worked independently, any processes for obtaining or confirming data from study investigators, and if applicable, details of automation tools used in the process. | p. 7 |
| Data items | 10a | List and define all outcomes for which data were sought. Specify whether all results that were compatible with each outcome domain in each study were sought (e.g. for all measures, time points, analyses), and if not, the methods used to decide which results to collect. | p. 7 |
|  | 10b | List and define all other variables for which data were sought (e.g. participant and intervention characteristics, funding sources). Describe any assumptions made about any missing or unclear information. | p. 7 |
| Study risk of bias assessment | 11 | Specify the methods used to assess risk of bias in the included studies, including details of the tool(s) used, how many reviewers assessed each study and whether they worked independently, and if applicable, details of automation tools used in the process. | p. 8 |
| Effect measures | 12 | Specify for each outcome the effect measure(s) (e.g. risk ratio, mean difference) used in the synthesis or presentation of results. | p. 8 |
| Synthesis methods | 13a | Describe the processes used to decide which studies were eligible for each synthesis (e.g. tabulating the study intervention characteristics and comparing against the planned groups for each synthesis (item #5)). | p. 5-7 |
|  | 13b | Describe any methods required to prepare the data for presentation or synthesis, such as handling of missing summary statistics, or data conversions. | p.8 |
|  | 13c | Describe any methods used to tabulate or visually display results of individual studies and syntheses. | p. 8  Appendix 5-7 |
|  | 13d | Describe any methods used to synthesize results and provide a rationale for the choice(s). If meta-analysis was performed, describe the model(s), method(s) to identify the presence and extent of statistical heterogeneity, and software package(s) used. | p. 8 |
|  | 13e | Describe any methods used to explore possible causes of heterogeneity among study results (e.g. subgroup analysis, meta-regression). | p. 8 |
|  | 13f | Describe any sensitivity analyses conducted to assess robustness of the synthesized results. | p. 8 |
| Reporting bias assessment | 14 | Describe any methods used to assess risk of bias due to missing results in a synthesis (arising from reporting biases). | p. 9 |
| Certainty assessment | 15 | Describe any methods used to assess certainty (or confidence) in the body of evidence for an outcome. | p.8 |
| **RESULTS** | | |  |
| Study selection | 16a | Describe the results of the search and selection process, from the number of records identified in the search to the number of studies included in the review, ideally using a flow diagram. | p.9 |
|  | 16b | Cite studies that might appear to meet the inclusion criteria, but which were excluded, and explain why they were excluded. | Appendix 4 |
| Study characteristics | 17 | Cite each included study and present its characteristics. | Refernce list p. 35 + table 1 |
| Risk of bias in studies | 18 | Present assessments of risk of bias for each included study. | p. 18 an referece list |
| Results of individual studies | 19 | For all outcomes, present, for each study: (a) summary statistics for each group (where appropriate) and (b) an effect estimate and its precision (e.g. confidence/credible interval), ideally using structured tables or plots. | Appendix 5-7 |
| Results of syntheses | 20a | For each synthesis, briefly summarise the characteristics and risk of bias among contributing studies. | p. 18-24 and Table 8 |
|  | 20b | Present results of all statistical syntheses conducted. If meta-analysis was done, present for each the summary estimate and its precision (e.g. confidence/credible interval) and measures of statistical heterogeneity. If comparing groups, describe the direction of the effect. | p.18-22 |
|  | 20c | Present results of all investigations of possible causes of heterogeneity among study results. | p.18-22 |
|  | 20d | Present results of all sensitivity analyses conducted to assess the robustness of the synthesized results. | p.18-22 |
| Reporting biases | 21 | Present assessments of risk of bias due to missing results (arising from reporting biases) for each synthesis assessed. | p.18-22 |
| Certainty of evidence | 22 | Present assessments of certainty (or confidence) in the body of evidence for each outcome assessed. | Appendix 8 and 9 |
| **DISCUSSION** | | |  |
| Discussion | 23a | Provide a general interpretation of the results in the context of other evidence. | 30-31 |
|  | 23b | Discuss any limitations of the evidence included in the review. | 32-33 |
|  | 23c | Discuss any limitations of the review processes used. | 32-33 |
|  | 23d | Discuss implications of the results for practice, policy, and future research. | 33-34 |
| **OTHER INFORMATION** | | |  |
| Registration and protocol | 24a | Provide registration information for the review, including register name and registration number, or state that the review was not registered. | p.5 |
|  | 24b | Indicate where the review protocol can be accessed, or state that a protocol was not prepared. | p.5 |
|  | 24c | Describe and explain any amendments to information provided at registration or in the protocol. | p. 5 |
| Support | 25 | Describe sources of financial or non-financial support for the review, and the role of the funders or sponsors in the review. | p. 34 |
| Competing interests | 26 | Declare any competing interests of review authors. | p. 34 |
| Availability of data, code and other materials | 27 | Report which of the following are publicly available and where they can be found: template data collection forms; data extracted from included studies; data used for all analyses; analytic code; any other materials used in the review. | Contact corresponding author |

*From:*  Page MJ, McKenzie JE, Bossuyt PM, Boutron I, Hoffmann TC, Mulrow CD, et al. The PRISMA 2020 statement: an updated guideline for reporting systematic reviews. BMJ 2021;372:n71. doi: 10.1136/bmj.n71

For more information, visit: <http://www.prisma-statement.org/>

**Appendix 2: Documentation of search strategy**

Date: Search 1: May 2021 + Search 2: April 2022

Topic/research question: **The efficacy of promotion and prevention interventions targeting psychological well-being and symptoms of Depression, Anxiety, Stress and Trauma among involuntary migrants: a systematic review and meta-analysis**

Name of researchers: Maja Andersson & Pia Enebrink, Inst. for klinisk neuro-vetenskap

Databases:

1. Medline(OVID)
2. PsycInfo(OVID)
3. Web of Science(Clarivate)

Total number of hits:

- Before deduplication: search 1: 4,316 search 2: 9,355 **total: 13,671**
- After within search deduplication: search 1: 2,400 search 2: 6,029 **total: 8,429**
- After between search deduplication: search 1: 2,400 search 2: 3, 226 **total: 5,626**

**Search 1**

1. Medline

| Interface: Ovid MEDLINE(R) and Epub Ahead of Print, In-Process & Other Non-Indexed Citations and Daily  Date of Search: 7^th^ May 2021  Number of hits: 2,089  Comment: In Ovid, two or more words are automatically searched as phrases; i.e. no quotation marks are needed | Field labels   - exp/ = exploded MeSH term - / = non exploded MeSH term - .ti,ab,kf. = title, abstract and author keywords - adjx = within x words, regardless of order - * = truncation of word for alternate endings |
| --- | --- |
| 1. exp "Emigrants and Immigrants"/  2. exp Human Migration/ 3. Refugees/  4. "Transients and Migrants"/  5. (asylum seek* or diaspora or emigrat* or emigrant* or floating population or immigrat* or immigrant* or migrat* or migrant* or newcomer* or newly arrived or refugee*).ti,ab,kf.  6. (displac* adj2 (adolescen* or boy? or child* or famil* or father* or forced or girl? or internal* or mass or minor* or mother* or parent* or person* or people* or population* or youth*)).ti,ab,kf.  7. or/1-6  8. Adjustment Disorders/  9. Antisocial Personality Disorder/  10. Anxiety/  11. exp Anxiety Disorders/  12. Child Behavior Disorders/  13. Depression/  14. Depressive Disorder/  15. Emotional Adjustment/  16. Mental Health/  17. Psychological Trauma/  18. Quality of Life/  19. exp Stress Disorders, Traumatic/  20. Stress, Psychological/  21. (anxiety or agoraphobia or antisocial behavio* or anxious* or depression or PTSD or quality of life or social phobia or war neuros* or well-being).ti,ab,kf.  22. ((disorder* or symptom*) adj2 (adjustment or affective or antisocial or anxiety or child behavio* or depressive or dysthymic or mood* or panic or phobi* or stress)).ti,ab,kf.  23. (grief or grieving).ti,ab,kf.  24. (mental* adj (disease* or disorder* or health or ill* or well-being)).ti,ab,kf.  25. (psycho* adj (distress* or stress or trauma)).ti,ab,kf.  26. or/8-25  27. 7 and 26  28. Cognitive Behavioral Therapy/  29. exp Desensitization, Psychologic/  30. Health Promotion/  31. Psychotherapy, Brief/  32. Psychotherapy, Psychodynamic/  33. (CBT or TF-CBT or EMDR or ISTDP or PDT).ti,ab,kf.  34. (cognitive adj2 therapy).ti,ab,kf.  35. (desensitization adj2 (eye movement* or psychologic*)).ti,ab,kf.  36. ((exposure or flooding or implosive or narrative or prolonged exposure) adj therapy).ti,ab,kf.  37. (psychotherapy adj (brief or dynamic or psychodynamic or short-term)).ti,ab,kf.  38. (intervention* or prevent* or program* or rehabilitation or therap* or treat*).ti,ab,kf.  39. (prevention & control or psychology or rehabilitation or therapy).fs.  40. or/28-39  41. Randomized Controlled Trial.pt.  42. Controlled Clinical Trial.pt.  43. Clinical Trials as Topic.sh.  44. randomi?ed.ab.  45. randomly.ab.  46. rct.ti,ab.  47. trial.ti.  48. Observational Study.pt.  49. (observ* adj3 (study or studies)).ab,ti.  50. exp Cohort Studies/  51. (cohort adj (study or studies)).ab,ti.  52. (cohort analy* or longitudinal or prospective* or retrospective*).ab,ti.  53. (follow up adj (study or studies)).ab,ti.  54. or/41-53  55. 7 and 26 and 40 and 54  56. limit 55 to (danish or english or french or german or norwegian or swedish) | |

2. Web of Science Core Collection

| Interface: Clarivate Analytics  Date of Search: 7^th^ 2021  Number of hits: 1,625 | Field labels   - TS/Topic = title, abstract, author keywords and Keywords Plus - NEAR/x = within x words, regardless of order - * = truncation of word for alternate endings   Note: sometimes “quotation marks” are needed for single search terms to avoid automatic term mapping (lemmatization). |
| --- | --- |
| #1 (”asylum seek*” or diaspora or emigrat* or emigrant* or ”floating population” or immigrat* or immigrant* or migrat* or migrant* or newcomer* or ”newly arrived” or refugee*) OR (displac* NEAR/1 (adolescen* or boy? or child* or famil* or father* or forced or girl? or internal* or mass or minor* or mother* or parent* or person* or people* or population* or youth*))  #2 (anxiety or agoraphobia or ”antisocial behavio*” or anxious* or depression or PTSD or ”quality of life” or ”social phobia” or ”war neuros*” or ”well-being”) OR ((disorder* or symptom*) NEAR/1 (adjustment or affective or antisocial or anxiety or ”child behavio*” or depressive or dysthymic or mood* or panic or phobi* or stress)) OR (grief or grieving) OR (mental* NEAR (disease* or disorder* or health or ill* or ”well-being”)) OR (psycho* NEAR (distress* or stress or trauma))  #3 (cognitive NEAR/1 therapy) OR (desensitization NEAR/1 (”eye movement*” or psychologic*)) OR  ((exposure or flooding or implosive or narrative or ”prolonged exposure”) NEAR therapy) OR  (psychotherapy NEAR (brief or dynamic or psychodynamic or ”short-term”)) OR (intervention* or prevent* or program* or rehabilitation or therap* or treat*) OR (CBT or ”TF-CBT” or EMDR or ISTDP or PDT)  #4 (”clinical trial” or randomi?ed or randomly or RCT) OR (observ* NEAR/2 (study or studies)) OR  (cohort NEAR (study or studies)) OR (”cohort analy*” or longitudinal or prospective* or retrospective*) OR (”follow up” NEAR (study or studies))  #5 #1 AND #2 AND #3 AND #4 **Refined by:** **LANGUAGES:** ( ENGLISH OR FRENCH OR DANISH OR GERMAN ) AND **DOCUMENT TYPES:** ( ARTICLE OR EARLY ACCESS OR REVIEW OR RETRACTED PUBLICATION ) | |

3. Psycinfo

| Interface: Ovid  Date of Search: 7^th^ May  Number of hits: 602  Comment: In Ovid, two or more words are automatically searched as phrases; i.e. no quotation marks are needed | Field labels   - exp/ = exploded controlled term - / = non exploded controlled term - .ti,ab,id. = title, abstract and author keywords - adjx = within x words, regardless of order - * = truncation of word for alternate endings |
| --- | --- |
| 1. immigration/  2. exp human migration/  3. (asylum seek* or diaspora or emigrat* or emigrant* or floating population or immigrat* or immigrant* or migrat* or migrant* or newcomer* or newly arrived or refugee*).ti,ab,id.  4. (displac* adj2 (adolescen* or boy? or child* or famil* or father* or forced or girl? or internal* or mass or minor* or mother* or parent* or person* or people* or population* or youth*)).ti,ab,id.  5. or/1-4  6. antisocial personality disorder/  7. anxiety/  8. exp anxiety disorders/  9. child behavior/  10. "depression (emotion)"/  11. exp major depression/  12. exp emotional adjustment/  13. exp mental health/  14. emotional trauma/  15. "quality of life"/  16. exp "stress and trauma related disorders"/  17. psychological stress/  18. (anxiety or agoraphobia or antisocial behavio* or anxious* or depression or PTSD or quality of life or social phobia or war neuros* or well-being).ti,ab,id.  19. ((disorder* or symptom*) adj2 (adjustment or affective or antisocial or anxiety or child behavio* or depressive or dysthymic or mood* or panic or phobi* or stress)).ti,ab,id.  20. (grief or grieving).ti,ab,id.  21. (mental* adj (disease* or disorder* or health or ill* or well-being)).ti,ab,id.  22. (psycho* adj (distress* or stress or trauma)).ti,ab,id.  23. or/6-22  24. exp cognitive behavior therapy/  25. eye movement desensitization therapy/  26. health promotion/  27. brief psychotherapy/  28. psychodynamic psychotherapy/  29. (CBT or TF-CBT or EMDR or ISTDP or PDT).ti,ab,id.  30. (cognitive adj2 therapy).ti,ab,id.  31. (desensitization adj2 (eye movement* or psychologic*)).ti,ab,id.  32. ((exposure or flooding or implosive or narrative or prolonged exposure) adj therapy).ti,ab,id.  33. (psychotherapy adj (brief or dynamic or psychodynamic or short-term)).ti,ab,id.  34. (intervention* or prevent* or program* or rehabilitation or therap* or treat*).ti,ab,id.  35. or/24-34  36. 5 and 23 and 35  37. (clinical trial or randomi?ed or randomly or RCT).ti,ab.  38. (observ* adj3 (study or studies)).ab,ti.  39. (cohort adj (study or studies)).ab,ti.  40. (cohort analy* or longitudinal or prospective* or retrospective*).ab,ti.  41. (follow up adj (study or studies)).ab,ti.  42. or/37-41  43. 36 and 42  44. limit 43 to (danish or english or french or german or norwegian or swedish) | |

**Search 2:**

1. Medline

| Interface: Ovid MEDLINE(R) and Epub Ahead of Print, In-Process & Other Non-Indexed Citations and Daily  Date of Search:11 April 2022  Number of hits: 4,295  Comment: In Ovid, two or more words are automatically searched as phrases; i.e. no quotation marks are needed | Field labels   - exp/ = exploded MeSH term - / = non exploded MeSH term - .ti,ab,kf. = title, abstract and author keywords - adjx = within x words, regardless of order - * = truncation of word for alternate endings |
| --- | --- |
| Database(s): **Ovid MEDLINE(R) and Epub Ahead of Print, In-Process, In-Data-Review & Other Non-Indexed Citations and Daily**1946 to April 08, 2022 Search Strategy:   \| **#** \| **Searches** \| **Results** \| \| --- \| --- \| --- \| \| 1 \| exp "Emigrants and Immigrants"/ \| 14627 \| \| 2 \| exp Human Migration/ \| 27391 \| \| 3 \| Refugees/ \| 12032 \| \| 4 \| "Transients and Migrants"/ \| 13287 \| \| 5 \| (asylum seek* or diaspora or emigrat* or emigrant* or floating population or immigrat* or immigrant* or migrat* or migrant* or newcomer* or newly arrived or refugee*).ti,ab,kf. \| 425918 \| \| 6 \| (displac* adj4 (adolescen* or boy? or child* or famil* or father* or forced or girl? or internal* or mass or minor* or mother* or parent* or person* or people* or population* or youth*)).ti,ab,kf. \| 6109 \| \| 7 \| or/1-6 \| 445502 \| \| 8 \| Adjustment Disorders/ \| 4285 \| \| 9 \| Antisocial Personality Disorder/ \| 10087 \| \| 10 \| Anxiety/ \| 97008 \| \| 11 \| exp Anxiety Disorders/ \| 86109 \| \| 12 \| Child Behavior Disorders/ \| 20723 \| \| 13 \| Depression/ \| 139418 \| \| 14 \| Depressive Disorder/ \| 74639 \| \| 15 \| Emotional Adjustment/ \| 949 \| \| 16 \| Mental Health/ \| 51894 \| \| 17 \| Psychological Trauma/ \| 1781 \| \| 18 \| Quality of Life/ \| 237751 \| \| 19 \| Resilience, Psychological/ \| 7765 \| \| 20 \| exp Stress Disorders, Traumatic/ \| 42315 \| \| 21 \| Stress, Psychological/ \| 130519 \| \| 22 \| (anxiety or agoraphobia or antisocial behavio* or anxious* or depression or PTSD or quality of life or social phobia or war neuros* or well-being).ti,ab,kf. \| 905500 \| \| 23 \| ((disorder* or symptom*) adj2 (adjustment or affective or antisocial or anxiety or child behavio* or depressive or dysthymic or mood* or panic or phobi* or stress)).ti,ab,kf. \| 223991 \| \| 24 \| (grief or grieving).ti,ab,kf. \| 9132 \| \| 25 \| (mental* adj (disease* or disorder* or health or ill* or well-being)).ti,ab,kf. \| 263928 \| \| 26 \| (psycho* adj (distress* or stress or trauma)).ti,ab,kf. \| 43064 \| \| 27 \| Parenting/ \| 19545 \| \| 28 \| exp Parent-Child Relations/ \| 59622 \| \| 29 \| Self Efficacy/ \| 23030 \| \| 30 \| ((adolescen* or boy? or child* or famil* or father* or girl? or minor* or mother* or parent* or youth*) adj3 (conflict* or interact* or relation*)).ti,ab,kf. \| 80921 \| \| 31 \| (competence or confidence or parenting or resilienc* or self-efficacy or skill*).ti,ab,kf. \| 940492 \| \| 32 \| or/8-31 \| 2294159 \| \| 33 \| Cognitive Behavioral Therapy/ \| 28675 \| \| 34 \| exp Desensitization, Psychologic/ \| 4154 \| \| 35 \| Health Promotion/ \| 79081 \| \| 36 \| Psychotherapy, Brief/ \| 3647 \| \| 37 \| Psychotherapy, Psychodynamic/ \| 708 \| \| 38 \| (CBT or TF-CBT or EMDR or ISTDP or PDT).ti,ab,kf. \| 30040 \| \| 39 \| (cognitive adj2 therap*).ti,ab,kf. \| 25912 \| \| 40 \| (desensitization adj2 (eye movement* or psychologic*)).ti,ab,kf. \| 635 \| \| 41 \| ((exposure or flooding or implosive or narrative or prolonged exposure) adj therapy).ti,ab,kf. \| 2336 \| \| 42 \| (psychotherapy adj (brief or dynamic or psychodynamic or short-term)).ti,ab,kf. \| 53 \| \| 43 \| (intervention* or prevent* or program* or rehabilitation or therap* or treat*).ti,ab,kf. \| 9822781 \| \| 44 \| (prevention & control or psychology or rehabilitation or therapy).fs. \| 4371370 \| \| 45 \| or/33-44 \| 11780422 \| \| 46 \| Randomized Controlled Trial.pt. \| 564125 \| \| 47 \| Controlled Clinical Trial.pt. \| 94806 \| \| 48 \| Clinical Trials as Topic.sh. \| 199674 \| \| 49 \| randomi?ed.ab. \| 665337 \| \| 50 \| randomly.ab. \| 379579 \| \| 51 \| rct.ti,ab. \| 27089 \| \| 52 \| trial.ti. \| 259833 \| \| 53 \| Observational Study.pt. \| 124812 \| \| 54 \| (observ* adj3 (study or studies)).ab,ti. \| 242202 \| \| 55 \| exp Cohort Studies/ \| 2326635 \| \| 56 \| (cohort adj (study or studies)).ab,ti. \| 267823 \| \| 57 \| (cohort analy* or longitudinal or prospective* or retrospective*).ab,ti. \| 1865937 \| \| 58 \| (follow up adj (study or studies)).ab,ti. \| 53295 \| \| 59 \| (before* adj2 after*).ti,ab. \| 308685 \| \| 60 \| (pre* adj3 post*).ti,ab. \| 368404 \| \| 61 \| or/46-60 \| 4653532 \| \| 62 \| 7 and 32 and 45 and 61 \| 4383 \| \| 63 \| limit 62 to (danish or english or french or german or norwegian or swedish) \| 4295 \| | |

2. Web of Science Core Collection

| Interface: Clarivate Analytics  Date of Search: 11 April 2022  Number of hits: 3,605 | Field labels   - TS/Topic = title, abstract, author keywords and Keywords Plus - NEAR/x = within x words, regardless of order - * = truncation of word for alternate endings   Note: sometimes “quotation marks” are needed for single search terms to avoid automatic term mapping (lemmatization). |
| --- | --- |
| #1 TS=(("asylum seek*" OR diaspora OR emigrat* OR emigrant* OR "floating population" OR immigrat* OR immigrant* OR migrat* OR migrant* OR newcomer* OR "newly arrived" OR refugee*) OR (displac* NEAR/3 (adolescen* OR boy$ OR child* OR famil* OR father* OR forced OR girl$ OR internal* OR mass OR minor* OR mother* OR parent* OR person* OR people* OR population* OR youth* )) )  #2 TS=(((((((anxiety OR agoraphobia OR "antisocial behavio*" OR anxious* OR depression OR PTSD OR "quality of life" OR "social phobia" OR "war neuros*" OR "well-being" ) OR ((disorder* OR symptom* ) NEAR/1 (adjustment OR affective OR antisocial OR anxiety OR "child behavio*" OR depressive OR dysthymic OR mood* OR panic OR phobi* OR stress )) OR (grief OR grieving ) OR (mental* NEAR/0 (disease* OR disorder* OR health OR ill* OR "well-being" )) OR (psycho* NEAR/0 (distress* OR stress OR trauma )) OR ((adolescen* OR boy$ OR child* OR famil* OR father* OR girl$ OR minor* OR mother* OR parent* OR youth* ) NEAR/2 (conflict* OR interact* OR relation* )) OR (competence OR confidence OR parenting OR resilienc* OR "self-efficacy" OR skill* ))))) ))  #3 TS=(((((((cognitive NEAR/1 therapy) OR (desensitization NEAR/1 ("eye movement*" OR psychologic*) ) OR ( (exposure OR flooding OR implosive OR narrative OR "prolonged exposure") NEAR/0 therapy)  OR (psychotherapy NEAR/0 (brief OR dynamic OR psychodynamic OR "short-term") ) OR (intervention* OR prevent* OR program* OR rehabilitation OR therap* or treat*) OR (CBT OR "TF-CBT" OR EMDR OR ISTDP OR PDT) ))))))  #4  TS=((((("clinical trial" or randomi?ed or randomly or RCT) OR (observ* NEAR/2 (study or studies) ) OR  (cohort NEAR/0 (study or studies) ) OR ("cohort analy*" or longitudinal or prospective* or retrospective*) OR ("follow up" NEAR/0 (study or studies) ))))) OR TI=((before* NEAR/1 after*)) OR AB=((before* NEAR/1 after*)) OR TI=(pre* NEAR/2 post*) OR AB=(pre* NEAR/2 post*)  #5 #1 AND #2 AND #3 AND #4 **Refined by:** **LANGUAGES:** ( ENGLISH OR FRENCH OR DANISH OR GERMAN ) | |

3. APA Psycinfo (EBSCO)

| Interface: EBSCO  Date of Search: 11 April 2022  Number of hits: 1,445 | Field labels   - DE = controlled term - / = non exploded controlled term - TI AB KW = title, abstract and author keywords - Nx = within x words, regardless of order - Wx= within x words, fixed word order - * = truncation of word for alternate endings |
| --- | --- |
| \| # \| Query \| Results \| \| --- \| --- \| --- \| \| S1 \| DE "Immigration" \| 24,947 \| \| S2 \| DE "Human Migration" OR DE "Geographical Mobility" OR DE "Refugees" \| 16,858 \| \| S3 \| TI ( ("asylum seek*" OR diaspora OR emigrat* OR emigrant* OR "floating population" OR immigrat* OR immigrant* OR migrat* OR migrant* OR newcomer* OR "newly arrived" OR refugee*) ) OR AB ( ("asylum seek*" OR diaspora OR emigrat* OR emigrant* OR "floating population" OR immigrat* OR immigrant* OR migrat* OR migrant* OR newcomer* OR "newly arrived" OR refugee*) ) OR KW ( ("asylum seek*" OR diaspora OR emigrat* OR emigrant* OR "floating population" OR immigrat* OR immigrant* OR migrat* OR migrant* OR newcomer* OR "newly arrived" OR refugee*) ) \| 71,606 \| \| S4 \| TI (displac* N3 (adolescen* OR boy# OR child* OR famil* OR father* OR forced OR girl# OR internal* OR mass OR minor* OR mother* OR parent* OR person* OR people* OR population* OR youth* )) OR AB (displac* N3 (adolescen* OR boy# OR child* OR famil* OR father* OR forced OR girl# OR internal* OR mass OR minor* OR mother* OR parent* OR person* OR people* OR population* OR youth* )) OR KW (displac* N3 (adolescen* OR boy# OR child* OR famil* OR father* OR forced OR girl# OR internal* OR mass OR minor* OR mother* OR parent* OR person* OR people* OR population* OR youth* )) \| 2,172 \| \| S5 \| S1 OR S2 OR S3 OR S4 \| 75,548 \| \| S6 \| DE "Antisocial Personality Disorder" \| 9,591 \| \| S7 \| DE "Anxiety" \| 89,938 \| \| S8 \| (DE "Anxiety Disorders" OR DE "Phobias" OR DE "Acrophobia" OR DE "Agoraphobia" OR DE "Claustrophobia" OR DE "Ophidiophobia" OR DE "School Phobia" OR DE "Social Phobia" OR DE "Obsessive Compulsive Disorder" OR DE "Hoarding Disorder" OR DE "Koro" OR DE "Hoarding Behavior" OR DE "Mental Disorders" OR DE "Castration Anxiety" OR DE "Generalized Anxiety Disorder" OR DE "Panic Attack" OR DE "Panic Disorder" OR DE "Separation Anxiety Disorder" OR DE "Trichotillomania") \| 200,654 \| \| S9 \| DE "Child Behavior" \| 8,570 \| \| S10 \| DE "Depression (Emotion)" \| 26,513 \| \| S11 \| DE "Major Depression" OR DE "Anaclitic Depression" OR DE "Dysthymic Disorder" OR DE "Endogenous Depression" OR DE "Late Life Depression" OR DE "Postpartum Depression" OR DE "Reactive Depression" OR DE "Recurrent Depression" OR DE "Treatment Resistant Depression" \| 147,162 \| \| S12 \| (DE "Emotional Adjustment" OR DE "Identity Crisis" OR DE "Emotional Control" OR DE "Anger Control") \| 23,028 \| \| S13 \| DE "Mental Health" OR DE "Athlete Mental Health" OR DE "Mental Health Disparities" OR DE "Mental Status" \| 86,962 \| \| S14 \| DE "Emotional Trauma" \| 15,950 \| \| S15 \| DE "Quality of Life" \| 62,001 \| \| S16 \| (DE "Stress and Trauma Related Disorders" OR DE "Acute Stress Disorder" OR DE "Adjustment Disorders" OR DE "Attachment Disorders" OR DE "Posttraumatic Stress Disorder" OR DE "Disinhibited Social Engagement Disorder" OR DE "Complex PTSD" OR DE "DESNOS") \| 40,955 \| \| S17 \| DE "Psychological Stress" \| 9,362 \| \| S18 \| TI ( (anxiety OR agoraphobia OR "antisocial behavio*" OR anxious* OR depression OR PTSD OR "quality of life" OR "social phobia" OR "war neuros*" OR "well-being" ) ) OR AB ( (anxiety OR agoraphobia OR "antisocial behavio*" OR anxious* OR depression OR PTSD OR "quality of life" OR "social phobia" OR "war neuros*" OR "well-being" ) ) OR KW ( (anxiety OR agoraphobia OR "antisocial behavio*" OR anxious* OR depression OR PTSD OR "quality of life" OR "social phobia" OR "war neuros*" OR "well-being" ) ) \| 595,861 \| \| S19 \| TI ( ((disorder* or symptom*) N1 (adjustment or affective or antisocial or anxiety or child behavio* or depressive or dysthymic or mood* or panic or phobi* or stress)) ) OR AB ( ((disorder* or symptom*) N1 (adjustment or affective or antisocial or anxiety or child behavio* or depressive or dysthymic or mood* or panic or phobi* or stress)) ) OR KW ( ((disorder* or symptom*) N1 (adjustment or affective or antisocial or anxiety or child behavio* or depressive or dysthymic or mood* or panic or phobi* or stress)) ) \| 224,877 \| \| S20 \| TI ( (grief OR grieving) ) OR AB ( (grief OR grieving) ) OR KW ( (grief OR grieving) ) \| 15,463 \| \| S21 \| TI ( (mental* W1 (disease* OR disorder* OR health OR ill* OR "well-being" )) ) OR AB ( (mental* W1 (disease* OR disorder* OR health OR ill* OR "well-being" )) ) OR KW ( (mental* W1 (disease* OR disorder* OR health OR ill* OR "well-being" )) ) \| 305,822 \| \| S22 \| TI ( (psycho* W1 (distress* OR stress OR trauma )) ) OR AB ( (psycho* W1 (distress* OR stress OR trauma )) ) OR KW ( (psycho* W1 (distress* OR stress OR trauma )) ) \| 36,438 \| \| S23 \| DE "Parenting" OR DE "Authoritarian Parenting" OR DE "Authoritative Parenting" OR DE "Childrearing Practices" OR DE "Coparenting" OR DE "Parent Child Communication" OR DE "Parent Child Relations" OR DE "Parental Involvement" OR DE "Parenthood Status" OR DE "Parenting Skills" OR DE "Parenting Style" OR DE "Permissive Parenting" OR DE "Child Care" OR DE "Child Care Workers" OR DE "Child Day Care" OR DE "Child Self Care" OR DE "Child Discipline" OR DE "Permissive Parenting" OR DE "Physical Discipline" OR DE "Toilet Training" OR DE "Weaning" OR DE "Father Child Communication" OR DE "Mother Child Communication" OR DE "Father Child Relations" OR DE "Mother Child Relations" OR DE "Parental Attitudes" OR DE "Parental Expectations" OR DE "Parent School Relationship") OR DE "Childlessness" OR DE "Authoritarian Parenting" OR DE "Authoritative Parenting" OR DE "Permissive Parenting" \| 123,674 \| \| S24 \| DE "Self-Efficacy" \| 26,050 \| \| S25 \| TI ( ((adolescen* OR boy# OR child* OR famil* OR father* OR girl# OR minor* OR mother* OR parent* OR youth* ) N2 (conflict* OR interact* OR relation* )) ) OR AB ( ((adolescen* OR boy# OR child* OR famil* OR father* OR girl# OR minor* OR mother* OR parent* OR youth* ) N2 (conflict* OR interact* OR relation* )) ) OR KW ( ((adolescen* OR boy# OR child* OR famil* OR father* OR girl# OR minor* OR mother* OR parent* OR youth* ) N2 (conflict* OR interact* OR relation* )) ) \| 142,265 \| \| S26 \| TI ( (competence OR confidence OR parenting OR resilienc* OR "self-efficacy" OR skill* ) ) OR AB ( (competence OR confidence OR parenting OR resilienc* OR "self-efficacy" OR skill* ) ) OR KW ( (competence OR confidence OR parenting OR resilienc* OR "self-efficacy" OR skill* ) ) \| 478,754 \| \| S27 \| DE "Resilience (Psychological)" \| 17,912 \| \| S28 \| S6 OR S7 OR S8 OR S9 OR S10 OR S11 OR S12 OR S13 OR S14 OR S15 OR S16 OR S17 OR S18 OR S19 OR S20 OR S21 OR S22 OR S23 OR S24 OR S25 OR S26 OR S27 \| 1,498,967 \| \| S29 \| DE "Cognitive Behavior Therapy" OR DE "Acceptance and Commitment Therapy" OR DE "Cognitive Processing Therapy" OR DE "Prolonged Exposure Therapy" \| 24,701 \| \| S30 \| DE "Eye Movement Desensitization Therapy" \| 1,742 \| \| S31 \| DE "Health Promotion" \| 36,222 \| \| S32 \| DE "Brief Psychotherapy" \| 5,913 \| \| S33 \| DE "Psychodynamic Psychotherapy" \| 3,820 \| \| S34 \| TI ( (CBT OR "TF-CBT" OR EMDR OR ISTDP OR PDT ) ) OR AB ( (CBT OR "TF-CBT" OR EMDR OR ISTDP OR PDT ) ) OR KW ( (CBT OR "TF-CBT" OR EMDR OR ISTDP OR PDT ) ) \| 18,197 \| \| S35 \| TI (cognitive N1 therap*) OR AB (cognitive N1 therap*) OR KW (cognitive N1 therap*) \| 36,614 \| \| S36 \| TI ( (desensitization N1 ("eye movement*" OR psychologic* )) ) OR AB ( (desensitization N1 ("eye movement*" OR psychologic* )) ) OR KW ( (desensitization N1 ("eye movement*" OR psychologic* )) ) \| 1,949 \| \| S37 \| TI ( ((exposure OR flooding OR implosive OR narrative OR "prolonged exposure" ) W1 therapy ) ) OR AB ( ((exposure OR flooding OR implosive OR narrative OR "prolonged exposure" ) W1 therapy ) ) OR KW ( ((exposure OR flooding OR implosive OR narrative OR "prolonged exposure" ) W1 therapy ) ) \| 4,872 \| \| S38 \| TI ( (psychotherapy W1 (brief OR dynamic OR psychodynamic OR "short-term" )) ) OR AB ( (psychotherapy W1 (brief OR dynamic OR psychodynamic OR "short-term" )) ) OR KW ( (psychotherapy W1 (brief OR dynamic OR psychodynamic OR "short-term" )) ) \| 467 \| \| S39 \| TI ( (intervention* OR prevent* OR program* OR rehabilitation OR therap* OR treat* ) ) OR AB ( (intervention* OR prevent* OR program* OR rehabilitation OR therap* OR treat* ) ) OR KW ( (intervention* OR prevent* OR program* OR rehabilitation OR therap* OR treat* ) ) \| 1,654,355 \| \| S40 \| S29 OR S30 OR S31 OR S32 OR S33 OR S34 OR S35 OR S36 OR S37 OR S38 OR S39 \| 1,666,525 \| \| S41 \| S5 AND S28 AND S40 \| 10,138 \| \| S42 \| TI ( ("clinical trial" OR randomi#ed OR randomly OR RCT ) ) OR AB ( ("clinical trial" OR randomi#ed OR randomly OR RCT ) ) \| 171,188 \| \| S43 \| TI ( (observ* N2 (study OR studies )) ) OR AB ( (observ* N2 (study OR studies )) ) \| 27,087 \| \| S44 \| TI ( (cohort W1 (study OR studies )) ) OR AB ( (cohort W1 (study OR studies )) ) \| 26,085 \| \| S45 \| TI ( ("cohort analy*" OR longitudinal OR prospective* OR retrospective* ) ) OR AB ( ("cohort analy*" OR longitudinal OR prospective* OR retrospective* ) ) \| 231,545 \| \| S46 \| TI ( ("follow up" W1 (study OR studies )) ) OR AB ( ("follow up" W1 (study OR studies )) ) \| 14,263 \| \| S47 \| TI (before* N1 after* ) OR AB (before* N1 after* ) \| 47,664 \| \| S48 \| TI (pre* N2 post*) OR AB (pre* N2 post*) \| 86,108 \| \| S49 \| S42 OR S43 OR S44 OR S45 OR S46 OR S47 OR S48 \| 528,137 \| \| S50 \| S41 AND S49 \| 1,475 \| \| S51 \| S50 - Limiters - Language: Danish, English, French, German, Norwegian, Swedish \| 1,445 \| | |

**Appendix 3: Participant characteristics**

*Parenting interventions.* Nine trials included refugees, of which two (Bjorknes et al., 2013; Osman et al., 2017) included both refugees and people whose residency was granted based on reunification with a family member with refugee status. Two trials included refugees and asylum seekers (Kaptan et al., 2022; Shaw et al., 2021) and two trials included internally displaced people (Dybdahl, 2001; Morris et al., 2012). Syrian refugees was the most prevalent population in interventions (*k* = 5 studies with samples entirely or primarily from Syria). Two interventions *(k =* 4) included participants from Somalia (Bjorknes et al., 2013 & 2015; Osman, Salari 2017; Osman, Flacking 2017). The remaining studies included people identified as Afghani or Rohingya (Shaw et al, 2020), Acholi from Uganda (Morris et al, 2012), Bosnia (Dybdahl, 2001), Karen from Myanmar/Burma (Ballard, 2017), Liberia, Sierra Leone, Congo and Burundi (Renzaho & Vignjevis, 2011) or had a variety of backgrounds (Kaptan et al., 2022). Three interventions were conducted in Scandinavia, three in the rest of Europe (Bosnia, the Netherlands, and the United Kingdom), 3 studies in the Middle East (Lebanon and Jordan), and one each in: Malaysia, Uganda, USA and Australia. In all but one intervention (Bjorknes et al., 2013; 2015) the participants had been in their current location for ≤ 5 years. Two studies reported that the participants lived in refugee camps (Lakkis et al, 2020; Morris et al, 2012), one reported that more than half lived in informal tented settlements (Sim et al, 2021), and one study described that the participants lived in refugee settlements or private accommodations (Dybdahl, 2001). The remaining studies did not explicitly describe the housing situation of the participants. The mean age of the parents in the parent interventions ranged from 26.6 to 44.5 (*M* = 33.3), four studies did not report the parents’ age. Six studies included only mothers, and one study 99.6 % mothers. Two additional studies included ≥ 95 % mothers. The remaining studies included between 31 to 48.9 % fathers, mostly about 40 %. The educational level of the included parents varied considerably between studies (see Table 3). Five studies did not report number of children. The average in the remaining studies ranged from 2.4 to 5.0. The included parents had children aged 0 to 18 years, and there was a large degree of overlap in age between studies. However, in four studies, all children were aged under 6 years. Ten studies reported the gender distribution of children. There was an approximately even distribution of girls and boys.

*Combined interventions.* Three of the combined studies included refugees and one study included forcibly displaced people (Puffer et al., 2017). Families of different nationalities were included in each study: Syrian, Bhutanese & Nepali, Afghani, and Burmese. The studies were conducted in Jordan, USA, Serbia, and Thailand. In one study the participants lived in reception centres (El-Khani et al., 2021), in two of the studies the families lived in the community (Ahktar, 2021; Puffer et al., 2017) and in one study housing situation was not described (Betancourt et al., 2020). The mean age of the parents ranged from 33.4 to 37.3 (*M*=38.17) and in three studies most participating parents were women (80 to 95 %). In one study the distribution of participating mothers and fathers was even (Betancourt et al., 2020). The gender distribution of children was quite even (*M* = 46%, R = 36 to 53.1% girls). The age of the child participants ranged from 7 – 8 years, to 15 - 18 years in all studies, except Akhtar et al. (2021) where the span was somewhat smaller (10 to 14 years).

**Appendix 4: Reason for exclusion**

A spreadsheet for exclusion of studies was used where reason of exclusion was recorded from left to right in the following order: wrong population (not forced migrant parents or not an psychological intervention on an universal or selective level), wrong study design (not a RCT or pre-post intervention study), wrong outcomes (none of the primary or secondary outcomes), too few participants (*n*<10), wrong publication (as a study protocol) or no access to data. The excluded studies could have been excluded for several reasons but at presented here from the first reason appearing in the prepared spreadsheet.

**Wrong population**

Abbott, A. (2016). The mental-health crisis among migrants. *Nature, 538*(7624), 158-160.

Abuelezam, N. N., El-Sayed, A. M., & Galea, S. (2018). The Health of Arab Americans in the United States: An Updated Comprehensive Literature Review. *Frontiers in public health, 6*, 262.

Abuelezam, N. N., & Fontenot, H. B. (2017). Depression Among Arab American and Arab Immigrant Women in the United States. *Nursing for women's health, 21*(5), 395-399.

Acarturk, C., Konuk, E., Cetinkaya, M., Senay, I., Sijbrandij, M., Cuijpers, P., & Aker, T. (2015). EMDR for Syrian refugees with posttraumatic stress disorder symptoms: results of a pilot randomized controlled trial. *European journal of psychotraumatology, 6*, 27414.

Acarturk, C., Konuk, E., Cetinkaya, M., Senay, I., Sijbrandij, M., Gulen, B., & Cuijpers, P. (2016). The efficacy of eye movement desensitization and reprocessing for post-traumatic stress disorder and depression among Syrian refugees: results of a randomized controlled trial. *Psychological Medicine, 46*(12), 2583-2593.

Acarturk, Z. C., Abuhamdeh, S., Jalal, B., Unaldi, N., Alyanak, B., Cetinkaya, M., . . . Hinton, D. (2019). Culturally Adapted Transdiagnostic CBT for SSRI-Resistant Turkish Adolescents: A Pilot Study. *American Journal of Orthopsychiatry, 89*(2), 222-227.

Adenauer, H., Catani, C., Gola, H., Keil, J., Ruf, M., Schauer, M., & Neuner, F. (2011). Narrative exposure therapy for PTSD increases top-down processing of aversive stimuli--evidence from a randomized controlled treatment trial. *BMC neuroscience, 12*, 127.

Alegria, M., Falgas-Bague, I., Collazos, F., Carmona Camacho, R., Lapatin Markle, S., Wang, Y., . . . Shrout, P. E. (2019). Evaluation of the Integrated Intervention for Dual Problems and Early Action Among Latino Immigrants With Co-occurring Mental Health and Substance Misuse Symptoms: A Randomized Clinical Trial. *JAMA network open, 2*(1), e186927.

Alsheikh Ali, A. S. a. S. (2020). Efficiency of Intervention Counseling Program on the Enhanced Psychological Well-being and Reduced Post-traumatic Stress Disorder Symptoms Among Syrian Women Refugee Survivors. *Clinical practice and epidemiology in mental health : CP & EMH, 16*, 134-141.

Alsmadi, A. M., Tawalbeh, L. I., Gammoh, O. S., Shawagfeh, M. Q., Zalloum, W., Ashour, A., & Attarian, H. (2018). The effect of Ginkgo biloba and psycho-education on stress, anxiety and fatigue among refugees. *Proceedings of Singapore Healthcare, 27*(1), 26-32.

Anders, M., & Christiansen, H. (2016). Unaccompanied Refugee Minors: A Systematic Review of Psychological Interventions. *Kindheit Und Entwicklung, 25*(4), 216-230.

Annan, J., Sim, A., Puffer, E. S., Salhi, C., & Betancourt, T. S. (2017). Improving Mental Health Outcomes of Burmese Migrant and Displaced Children in Thailand: a Community-Based Randomized Controlled Trial of a Parenting and Family Skills Intervention. *Prevention science : the official journal of the Society for Prevention Research, 18*(7), 793-803.

Arabacioglu, B., & Bagceli Kahraman, P. (2020). The Effect of Social Skills Education on 60-69 Months Old Syrian Children's Social Skills, Problem Solving and Adaptation. *Cukurova University Faculty of Education Journal, 49*(2), 734-768.

Asghar, K., Mayevskaya, Y., Sommer, M., Razzaque, A., Laird, B., Khan, Y., . . . Stark, L. (2018). Promoting Adolescent Girls' Well-Being in Pakistan: a Mixed-Methods Study of Change Over Time, Feasibility, and Acceptability, of the COMPASS Program. *Prevention science : the official journal of the Society for Prevention Research, 19*(8), 1030-1042.

Askovic, M., Watters, A. J., Coello, M., Aroche, J., Harris, A. W. F., & Kropotov, J. (2020). Evaluation of Neurofeedback for Posttraumatic Stress Disorder Related to Refugee Experiences Using Self-Report and Cognitive ERP Measures. *Clinical EEG and neuroscience, 51*(2), 79-86.

Ayala, G. X., Elder, J. P., Campbell, N. R., Arredondo, E., Baquero, B., Crespo, N. C., & Slymen, D. J. (2010). Longitudinal intervention effects on parenting of the Aventuras para Ninos study. *American journal of preventive medicine, 38*(2), 154-162.

Ayoughi, S., Missmahl, I., Weierstall, R., & Elbert, T. (2012). Provision of mental health services in resource-poor settings: a randomised trial comparing counselling with routine medical treatment in North Afghanistan (Mazar-e-Sharif). *BMC psychiatry, 12*.

Baggerly, J. N., & Corbin, T. (2021). Group counseling for southeast Asian refugee children with trauma symptoms: Pilot study results and practical guidelines. *Journal of Child and Adolescent Counseling, 7*(2), 87-99.

Barenbaum, J., Ruchkin, V., & Schwab-Stone, M. (2004). The psychosocial aspects of children exposed to war: practice and policy initiatives. *Journal of Child Psychology and Psychiatry, 45*(1), 41-62.

Barrett, P. M., Sonderegger, R., & Xenos, S. (2003). Using FRIENDS to combat anxiety and adjustment problems among young migrants to Australia: A national trial. *Clinical Child Psychology and Psychiatry, 8*(2), 241-260.

Barnett, M. L., Davis, E. M., Callejas, L. M., White, J. V., Acevedo-Polakovich, I. D., Niec, L. N., & Jent, J. F. (2016). The development and evaluation of a natural helpers' training program to increase the engagement of urban, Latina/o families in parent-child interaction therapy. *Children and Youth Services Review, 65*, 17-25.

Barwick, M., Urajnik, D., Sumner, L., Cohen, S., Reid, G., Engel, K., & Moore, J. E. (2013). Profiles and service utilization for children accessing a mental health walk-in clinic versus usual care. *Journal of evidence-based social work, 10*(4), 338-352.

Bauby, C., Dandres, A. M., & Lejeune, C. (2010). [Gennevilliers parents-babies unit: PMI-pedopsychiatrist partnership]. *Unite parents-bebes de Gennevilliers: partenariat PMI-pedopsychiatrie., 17*(6), 624-625.

Beck, B. D., Messel, C., Meyer, S. L., Cordtz, T. O., Sogaard, U., Simonsen, E., & Moe, T. (2018). Feasibility of trauma-focused guided imagery and music with adult refugees diagnosed with PTSD: A pilot study. *Nordic Journal of Music Therapy, 27*(1), 67-86.

Beeber, L. S., Lewis, V. S., Cooper, C., Maxwell, L., & Sandelowski, M. (2009). Meeting the "Now" Need: PMH-APRN-- Interpreter Teams Provide In-Home Mental Health Intervention for Depressed Latina Mothers With Limited English Proficiency. *Journal of the American Psychiatric Nurses Association, 15*(4), 249-259.

Bennett-Conroy, W. (2012). Engaging parents of eighth grade students in parent-teacher bidirectional communication. *The School Community Journal, 22*(2), 87-110.

Bentley, J. A., Feeny, N. C., Dolezal, M. L., Klein, A., Marks, L. H., Graham, B., & Zoellner, L. A. (2021). Islamic Trauma Healing: Integrating Faith and Empirically Supported Principles in a Community-Based Program. *Cognitive and Behavioral Practice, 28*(2), 167-192.

Bernardi, J., Dahiya, M., & Jobson, L. (2019). Culturally modified cognitive processing therapy for Karen refugees with posttraumatic stress disorder: A pilot study. *Clinical psychology & psychotherapy, 26*(5), 531-539.

Bernhardt, L. J., Lin, S., Swegman, C., Sellke, R., Vu, A., Solomon, B. S., & Cuneo, C. N. (2019). The Refugee Health Partnership: A Longitudinal Experiential Medical Student Curriculum in Refugee/Asylee Health. *Academic medicine : journal of the Association of American Medical Colleges, 94*(4), 544-549.

Bernstein, K., Park, S. Y., Hahm, S., Lee, Y. N., Seo, J. Y., & Nokes, K. M. (2016). Efficacy of a Culturally Tailored Therapeutic Intervention Program for Community Dwelling Depressed Korean American Women: A Non-Randomized Quasi-Experimental Design Study. *Archives of Psychiatric Nursing, 30*(1), 19-26.

Betancourt, T. S., Newnham, E. A., Brennan, R. T., Verdeli, H., Borisova, I., Neugebauer, R., . . . Bolton, P. (2012). Moderators of treatment effectiveness for war-affected youth with depression in northern Uganda. *The Journal of adolescent health : official publication of the Society for Adolescent Medicine, 51*(6), 544-550.

Betancourt, T. S., Yudron, M., Wheaton, W., & Smith-Fawzi, M. C. (2012). Caregiver and adolescent mental health in Ethiopian Kunama refugees participating in an emergency education program. *The Journal of adolescent health : official publication of the Society for Adolescent Medicine, 51*(4), 357-365.

Bhavsar, V., Jannesari, S., McGuire, P., MacCabe, J. H., Das-Munshi, J., Bhugra, D., . . . Hatch, S. L. (2021). The association of migration and ethnicity with use of the Improving Access to Psychological Treatment (IAPT) programme: a general population cohort study. *Social psychiatry and psychiatric epidemiology*.

Birman, D., Beehler, S., Harris, E. M., Everson, M. L., Batia, K., Liautaud, J., . . . Cappella, E. (2008). International family, adult, and child enhancement services (FACES): A community-based comprehensive services model for refugee children in resettlement. *American Journal of Orthopsychiatry, 78*(1), 121-132.

Björn, G. J., Bodén, C., Sydsjö, G., & Gustafsson, P. A. (2013). Brief family therapy for refugee children. *The Family Journal, 21*(3), 272-278.

Blanchet, K., Ramesh, A., Frison, S., Warren, E., Hossain, M., Smith, J., . . . Roberts, B. (2017). Evidence on public health interventions in humanitarian crises. *Lancet, 390*(10109), 2287-2296.

Blom, M. B. J., Hoek, H. W., Spinhoven, P., Hoencamp, E., Haffmans, P. M. J., & van Dyck, R. (2010). Treatment of Depression in Patients from Ethnic Minority Groups in the Netherlands. *Transcultural Psychiatry, 47*(3), 473-490.

Boehnlein, J. K., Kinzie, J. D., Sekiya, U., Riley, C., Pou, K., & Rosborough, B. (2004). A ten-year treatment outcome study of traumatized Cambodian refugees. *The Journal of nervous and mental disease, 192*(10), 658-663.

Boge, K., Karnouk, C., Hahn, E., Schneider, F., Habel, U., Banaschewski, T., . . . Bajbouj, M. (2020). Mental health in refugees and asylum seekers (MEHIRA): study design and methodology of a prospective multicentre randomized controlled trail investigating the effects of a stepped and collaborative care model. *European archives of psychiatry and clinical neuroscience, 270*(1), 95-106.

Bolton, P., Bass, J., Betancourt, T., Speelman, L., Onyango, G., Clougherty, K. F., . . . Verdeli, H. (2007). Interventions for depression symptoms among adolescent survivors of war and displacement in northern Uganda: a randomized controlled trial. *JAMA, 298*(5), 519-527.

Bolton, P., Bass, J. K., Zangana, G. A. S., Kamal, T., Murray, S. M., Kaysen, D., . . . Rosenblum, M. (2014). A randomized controlled trial of mental health interventions for survivors of systematic violence in Kurdistan, Northern Iraq. *BMC psychiatry, 14*.

Bolton, P., Lee, C., Haroz, E. E., Murray, L., Dorsey, S., Robinson, C., . . . Bass, J. (2014). A transdiagnostic community-based mental health treatment for comorbid disorders: development and outcomes of a randomized controlled trial among Burmese refugees in Thailand. *PLoS medicine, 11*(11), e1001757.

Boyce, L. K., Innocenti, M. S., Roggman, L. A., Jump Norman, V. K., & Ortiz, E. (2010). Telling stories and making books: Evidence for an intervention to help parents in migrant Head Start families support their children's language and literacy. *Early Education and Development, 21*(3), 343-371.

Boyd, A. T., Cookson, S. T., Anderson, M., Bilukha, O. O., Brennan, M., Handzel, T., . . . Gerber, M. (2017). Centers for Disease Control and Prevention Public Health Response to Humanitarian Emergencies, 2007-2016. *Emerging Infectious Diseases, 23*, S196-S202.

Bradley, G. M., Couchman, G. M., Perlesz, A., Nguyen, A. T., Singh, B., & Riess, C. (2006). Multiple-family group treatment for English- and Vietnamese-speaking families living with schizophrenia. *Psychiatric services (Washington, D.C.), 57*(4), 521-530.

Brown, F. L., Carswell, K., Augustinavicius, J., Adaku, A., Leku, M. R., White, R. G., . . . Tol, W. A. (2018). Self Help Plus: study protocol for a cluster-randomised controlled trial of guided self-help with South Sudanese refugee women in Uganda. *Global mental health (Cambridge, England), 5*, e27.

Bruhn, M., Rees, S., Mohsin, M., Silove, D., & Carlsson, J. (2018). The Range and Impact of Postmigration Stressors During Treatment of Trauma-Affected Refugees. *Journal of Nervous and Mental Disease, 206*(1), 61-68.

Bruno, W., Kitamura, A., Najjar, S., Seita, A., & Al-Delaimy, W. K. (2019). Assessment of mental health and psycho-social support pilot program's effect on intended stigmatizing behavior at the Saftawi Health Center, Gaza: a cross-sectional study. *Journal of mental health (Abingdon, England), 28*(4), 436-442.

Bugental, D. B., & Schwartz, A. (2009). A cognitive approach to child mistreatment prevention among medically at-risk infants. *Developmental psychology, 45*(1), 284-288.

Buhmann, C., Andersen, I., Mortensen, E. L., Ryberg, J., Nordentoft, M., & Ekstrom, M. (2015). Cognitive behavioral psychotherapeutic treatment at a psychiatric trauma clinic for refugees: description and evaluation. *Torture : quarterly journal on rehabilitation of torture victims and prevention of torture, 25*(1), 17-32.

Buhmann, C., Mortensen, E. L., Nordentoft, M., Ryberg, J., & Ekstrom, M. (2015). Follow-up study of the treatment outcomes at a psychiatric trauma clinic for refugees. *Torture : quarterly journal on rehabilitation of torture victims and prevention of torture, 25*(1), 1-16.

Buhmann, C. B. (2014). Traumatized refugees: morbidity, treatment and predictors of outcome. *Danish medical journal, 61*(8), B4871.

Buhmann, C. B., Nordentoft, M., Ekstroem, M., Carlsson, J., & Mortensen, E. L. (2016). The effect of flexible cognitive-behavioural therapy and medical treatment, including antidepressants on post-traumatic stress disorder and depression in traumatised refugees: pragmatic randomised controlled clinical trial. *The British journal of psychiatry : the journal of mental science, 208*(3), 252-259.

Buhmann, C. B., Nordentoft, M., Ekstroem, M., Carlsson, J., & Mortensen, E. L. (2018). Long-term treatment effect of trauma-affected refugees with flexible cognitive behavioural therapy and antidepressants. *Psychiatry research, 264*, 217-223.

Burruss, N. C., Shaltout, Y., Hamilton, C. T., Oberti, D., Linton, J. M., & Brown, C. L. (2021). Arts-based therapy: a pilot program for immigrant and refugee children. *Vulnerable Children and Youth Studies, 16*(3), 253-258.

Carlsson, J., Sonne, C., Vindbjerg, E., & Mortensen, E. L. (2018). Stress management versus cognitive restructuring in trauma-affected refugees-A pragmatic randomised study. *Psychiatry research, 266*, 116-123.

Carlsson, J. M., Mortensen, E. L., & Kastrup, M. (2005). A follow-up study of mental health and health-related quality of life in tortured refugees in multidisciplinary treatment. *The Journal of nervous and mental disease, 193*(10), 651-657.

Carlsson, J. M., Olsen, D. R., Kastrup, M., & Mortensen, E. L. (2010). Late mental health changes in tortured refugees in multidisciplinary treatment. *The Journal of nervous and mental disease, 198*(11), 824-828.

Cardeli, E., Phan, J., Mulder, L., Benson, M., Adhikari, R., & Ellis, B. H. (2020). Bhutanese Refugee Youth: The Importance of Assessing and Addressing Psychosocial Needs in a School Setting. *The Journal of school health, 90*(9), 731-742.

Catani, C., Kohiladevy, M., Ruf, M., Schauer, E., Elbert, T., & Neuner, F. (2009). Treating children traumatized by war and Tsunami: a comparison between exposure therapy and meditation-relaxation in North-East Sri Lanka. *BMC psychiatry, 9*, 22.

Ceballos, P. L., & Bratton, S. C. (2010). Empowering Latino families: Effects of a culturally responsive intervention for low-income immigrant Latino parents on children's behaviors and parental stress. *Psychology in the Schools, 47*(8), 761-775.

Ceballos, P. (2009). *School-based child parent relationship therapy (CPRT) with low income first generation immigrant Hispanic parents: Effects on child behavior and parent-child relationship stress.* (69). ProQuest Information & Learning,

Chiu, S. J., Lin, I. F., Chou, Y. T., & Chien, L. Y. (2020). Family quality of life among Taiwanese children with developmental delay before and after early intervention. *Journal of intellectual disability research : JIDR, 64*(8), 589-601.

Colombari Figueroa, S., Stafford, R. S., Heaney, C. A., & Rosas, L. G. (2018). The Effect of a Behavioral Weight-Loss Intervention on Depressive Symptoms Among Latino Immigrants in a Randomized Controlled Trial. *Journal of immigrant and minority health, 20*(5), 1182-1189.

Cowell, J. M., McNaughton, D., Ailey, S., Gross, D., & Fogg, L. (2009). Clinical Trail Outcomes of the Mexican American Problem Solving Program (MAPS). *Hispanic health care international : the official journal of the National Association of Hispanic Nurses, 7*(4), 179-189.

Culhane-Pera, K. A., Peterson, K. A., Crain, A. L., Center, B. A., Lee, M., Her, B., & Xiong, T. (2005). Group visits for Hmong adults with type 2 diabetes mellitus: A pre-post analysis. *Journal of Health Care for the Poor and Underserved, 16*(2), 315-327.

Cunningham, C. E., Bremner, R., & Boyle, M. (1995). Large group community-based parenting programs for families of preschoolers at risk for disruptive behaviour disorders: utilization, cost effectiveness, and outcome. *Journal of child psychology and psychiatry, and allied disciplines, 36*(7), 1141-1159.

d'Ardenne, P., Ruaro, L., Cestari, L., Fakhoury, W., & Priebe, S. (2007). Does interpreter-mediated CBT with traumatized refugee people work? A comparison of patient outcomes in East London. *Behavioural and Cognitive Psychotherapy, 35*(3), 293-301.

Dajani, R., Hadfield, K., van Uum, S., Greff, M., & Panter-Brick, C. (2018). Hair cortisol concentrations in war-affected adolescents: A prospective intervention trial. *Psychoneuroendocrinology, 89*, 138-146.

Daou, K., Daou, L., & Cousineau-Perusse, M. (2022). A family-based intervention for refugee children. *International Journal of Social Welfare, 31*(1), 56-65.

Davey, H. L., Tough, S. C., Adair, C. E., & Benzies, K. M. (2011). Risk Factors for Sub-Clinical and Major Postpartum Depression Among a Community Cohort of Canadian Women. *Maternal and Child Health Journal, 15*(7), 866-875.

de Graaff, A. M., Cuijpers, P., Acarturk, C., Bryant, R., Burchert, S., Fuhr, D. C., . . . Sijbrandij, M. (2020). Effectiveness of a peer-refugee delivered psychological intervention to reduce psychological distress among adult Syrian refugees in the Netherlands: study protocol. *European journal of psychotraumatology, 11*(1), 1694347.

de Graaff, A. M., Cuijpers, P., McDaid, D., Park, A., Woodward, A., Bryant, R. A., . . . Consortium, S. (2020). Peer-provided Problem Management Plus (PM plus ) for adult Syrian refugees: a pilot randomised controlled trial on effectiveness and cost-effectiveness. *Epidemiology and Psychiatric Sciences, 29*.

De La Rosa-Lopes, G. M. F. (2018). *A group intervention for children who have experienced immigration-related family separation: A mixed-methods investigation.* (79). ProQuest Information & Learning.

Demezier, D. (2021). *Biculturalism, familism, and parenting styles of Haitian parents with juvenile youth: Impact of a family-based intervention.* (82). ProQuest Information & Learning,

Dolan, C. T., Kim, H. Y., Brown, L., Gjicali, K., Borsani, S., El Houchaimi, S., & Aber, J. L. (2022). Supporting Syrian Refugee Children's Academic and Social-Emotional Learning in National Education Systems: A Cluster Randomized Controlled Trial of Nonformal Remedial Support and Mindfulness Programs in Lebanon. *American Educational Research Journal*, 42.

Doumit, R., Kazandjian, C., & Militello, L. K. (2020). COPE for Adolescent Syrian Refugees in Lebanon: A Brief Cognitive-Behavioral Skill-Building Intervention to Improve Quality of Life and Promote Positive Mental Health. *Clinical Nursing Research, 29*(4), 226-234.

Drozdek, B., Kamperman, A. M., Bolwerk, N., Tol, W. A., & Kleber, R. J. (2012). Group therapy with male asylum seekers and refugees with posttraumatic stress disorder: a controlled comparison cohort study of three day-treatment programs. *The Journal of nervous and mental disease, 200*(9), 758-765.

Drozdek, B., Kamperman, A. M., Tol, W. A., Knipscheer, J. W., & Kleber, R. J. (2014). Seven-year follow-up study of symptoms in asylum seekers and refugees with PTSD treated with trauma-focused groups. *Journal of clinical psychology, 70*(4), 376-387.

Dumas, J. E., Arriaga, X. B., Begle, A. M., & Longoria, Z. N. (2011). Child and parental outcomes of a group parenting intervention for Latino families: A pilot study of the CANNE program. *Cultural diversity & ethnic minority psychology, 17*(1), 107-115.

Dunne, J. E. (1993). Cambodian follow-up. *Journal of the American Academy of Child and Adolescent Psychiatry, 32*(6), 1305-1306.

Edelblute, H. B., Clark, S., Mann, L., McKenney, K. M., Bischof, J. J., & Kistler, C. (2014). Promotoras across the border: a pilot study addressing depression in Mexican women impacted by migration. *Journal of immigrant and minority health, 16*(3), 492-500.

Ekblad, S. (1994). [Importance of follow-up of refugee children. Risk factors are changing during the different phases of the crisis]. *Viktigt folja upp asylsokande barn. Riskfaktorerna andras under krisfaserna., 91*(44), 4012-4017.

Ekblad, S. (2020). To Increase Mental Health Literacy and Human Rights Among New-Coming, Low-Educated Mothers With Experience of War: A Culturally, Tailor-Made Group Health Promotion Intervention With Participatory Methodology Addressing Indirectly the Children. *Frontiers in Psychiatry, 11*, 611.

El-Khani, A., Cartwright, K., Ang, C., Henshaw, E., Tanveer, M., & Calam, R. (2018). Testing the feasibility of delivering and evaluating a child mental health recovery program enhanced with additional parenting sessions for families displaced by the Syrian conflict: A pilot study. Peace and Conflict: Journal of Peace Psychology, 24(2), 188–200. [https://doi.org/10.1037/pac0000287](https://psycnet.apa.org/doi/10.1037/pac0000287)

El-Khani, A., Maalouf, W., Baker, D. A., Zahra, N., Noubani, A., & Cartwright, K. (2020). Caregiving for children through conflict and displacement: a pilot study testing the feasibility of delivering and evaluating a light touch parenting intervention for caregivers in the West Bank. *International journal of psychology : Journal international de psychologie, 55*, 26-39.

Ellis, B. H., Miller, A. B., Abdi, S., Barrett, C., Blood, E. A., & Betancourt, T. S. (2013). Multi-tier mental health program for refugee youth. *Journal of consulting and clinical psychology, 81*(1), 129-140.

Erdemir, E. (2022). Summer Preschools for Syrian Refugee and Host Community Children in Turkey: A Model of Contextually Sensitive Early Intervention. *Early Education and Development*, 27.

Erickson, P. I. (1994). Lessons from a repeat pregnancy prevention program for Hispanic teenage mothers in east Los Angeles. *Family planning perspectives, 26*(4), 174-178.

Ertl, V., Pfeiffer, A., Schauer, E., Elbert, T., & Neuner, F. (2011). Community-implemented trauma therapy for former child soldiers in Northern Uganda: a randomized controlled trial. *JAMA, 306*(5), 503-512.

Esala, J. J., Vukovich, M. M., Hanbury, A., Kashyap, S., & Joscelyne, A. (2018). Collaborative care for refugees and torture survivors: Key findings from the literature. *Traumatology, 24*(3), 168-185.

Eylem, O., van Straten, A., de Wit, L., Rathod, S., Bhui, K., & Kerkhof, A. J. F. M. (2021). Reducing suicidal ideation among Turkish migrants in the Netherlands and in the UK: the feasibility of a randomised controlled trial of a guided online intervention. *Pilot and feasibility studies, 7*(1), 30.

Fabrizio, C. S., Stewart, S. M., Ip, A. K. Y., & Lam, T. H. (2014). Enhancing the Parent-Child Relationship: A Hong Kong Community-Based Randomized Controlled Trial. *Journal of Family Psychology, 28*(1), 42-53.

Falb, K. L., Tanner, S., Ward, L., Erksine, D., Noble, E., Assazenew, A., . . . Stark, L. (2016). Creating opportunities through mentorship, parental involvement, and safe spaces (COMPASS) program: multi-country study protocol to protect girls from violence in humanitarian settings. *BMC public health, 16*, 231.

Falgas-Bague, I., Wang, Y., Banerjee, S., Ali, N., DiMarzio, K., Palao Vidal, D., & Alegria, M. (2019). Predictors of Adherence to Treatment in Behavioral Health Therapy for Latino Immigrants: The Importance of Trust. *Frontiers in psychiatry, 10*, 817.

Falkenstrom, F. (2010). Does psychotherapy for young adults in routine practice show similar results as therapy in randomized clinical trials? *Psychotherapy research : journal of the Society for Psychotherapy Research, 20*(2), 181-192.

Fazel, M., Doll, H., & Stein, A. (2009). A school-based mental health intervention for refugee children: an exploratory study. *Clinical Child Psychology and Psychiatry, 14*(2), 297-309.

Feddes, A. R., Mann, L., & Doosje, B. (2015). Increasing self-esteem and empathy to prevent violent radicalization: a longitudinal quantitative evaluation of a resilience training focused on adolescents with a dual identity. *Journal of Applied Social Psychology, 45*(7), 400-411.

Fischmann, T., Asseburg, L. K., Green, J., Hug, F., Neubert, V., Wan, M., & Leuzinger-Bohleber, M. (2020). Can Psychodynamically Oriented Early Prevention for "Children-at-Risk" in Urban Areas With High Social Problem Density Strengthen Their Developmental Potential? A Cluster Randomized Trial of Two Kindergarten-Based Prevention Programs. *Frontiers in psychology, 11*, 599477.

Flynn, A., Gonzalez, V., Mata, M., Salinas, L. A., & Atkins, A. (2020). Integrated care improves mental health in a medically underserved U.S.-Mexico border population. *Families, systems & health : the journal of collaborative family healthcare, 38*(2), 105-115.

Foka, S., Hadfield, K., Pluess, M., & Mareschal, I. (2021). Promoting well-being in refugee children: An exploratory controlled trial of a positive psychology intervention delivered in Greek refugee camps. *Development and psychopathology, 33*(1), 87-95.

Folkes, C. E. (2002). Thought field therapy and trauma recovery. *International journal of emergency mental health, 4*(2), 99-103.

Garcia-Huidobro, D., Diaspro-Higuera, M. O., Palma, D., Palma, R., Ortega, L., Shlafer, R., . . . Allen, M. L. (2019). Adaptive Recruitment and Parenting Interventions for Immigrant Latino Families with Adolescents. *Prevention science : the official journal of the Society for Prevention Research, 20*(1), 56-67.

Getanda, E. M., & Vostanis, P. (2020). Feasibility evaluation of psychosocial intervention for internally displaced youth in Kenya. *Journal of mental health (Abingdon, England)*, 1-9.

Goodkind, J. R., Bybee, D., Hess, J. M., Amer, S., Ndayisenga, M., Greene, R. N., . . . Pannah, M. (2020). Randomized Controlled Trial of a Multilevel Intervention to Address Social Determinants of Refugee Mental Health. *American journal of community psychology, 65*(3), 272-289.

Goodkind, J. R., Hess, J. M., Isakson, B., LaNoue, M., Githinji, A., Roche, N., . . . Parker, D. P. (2014). Reducing refugee mental health disparities: a community-based intervention to address postmigration stressors with African adults. *Psychological services, 11*(3), 333-346.

Goossens, F. X., Onrust, S. A., Monshouwer, K., & de Castro, B. O. (2016). Effectiveness of an empowerment program for adolescent second generation migrants: A cluster randomized controlled trial. *Children and Youth Services Review, 64*, 128-135.

Gordon, J. S., Staples, J. K., Blyta, A., Bytyqi, M., & Wilson, A. T. (2008). Treatment of posttraumatic stress disorder in postwar kosovar adolescents using mind-body skills groups: A randomized controlled trial. *Journal of Clinical Psychiatry, 69*(9), 1469-1476.

Gormez, V., Kilic, H. N., Orengul, A. C., Demir, M. N., Mert, E. B., Makhlouta, B., . . . Semerci, B. (2017). Evaluation of a school-based, teacher-delivered psychological intervention group program for trauma-affected Syrian refugee children in Istanbul, Turkey. *Psychiatry and Clinical Psychopharmacology, 27*(2), 125-131.

Greenfield, P. M., Espinoza, G., Monterroza-Brugger, M., Ruedas-Gracia, N., & Manago, A. M. (2020). Long-term parent–child separation through serial migration: Effects of a post-reunion intervention. *The School Community Journal, 30*(1), 267-298.

Grochtdreis, T., Rohr, S., Jung, F. U., Nagl, M., Renner, A., Kersting, A., . . . Dams, J. (2021). Health Care Services Utilization and Health-Related Quality of Life of Syrian Refugees with Post-Traumatic Stress Symptoms in Germany (the Sanadak Trial). *International journal of environmental research and public health, 18*(7).

Gupta, L., & Zimmer, C. (2008). Psychosocial intervention for war-affected children in Sierra Leone. *The British journal of psychiatry : the journal of mental science, 192*(3), 212-216.

Gurung, A., Subedi, P., Zhang, M., Li, C., Kelly, T., Kim, C., & Yun, K. (2020). Culturally-Appropriate Orientation Increases the Effectiveness of Mental Health First Aid Training for Bhutanese Refugees: Results from a Multi-state Program Evaluation. *Journal of immigrant and minority health, 22*(5), 957-964.

Haagen, J. F. G., Ter Heide, F. J. J., Mooren, T. M., Knipscheer, J. W., & Kleber, R. J. (2017). Predicting post-traumatic stress disorder treatment response in refugees: Multilevel analysis. *The British journal of clinical psychology, 56*(1), 69-83.

Haar, K., El-Khani, A., Molgaard, V., Maalouf, W., & Afghanistan field implementation, t. (2020). Strong families: a new family skills training programme for challenged and humanitarian settings: a single-arm intervention tested in Afghanistan. *BMC public health, 20*(1), 634.

Halvorsen, J. O., & Stenmark, H. (2010). Narrative exposure therapy for posttraumatic stress disorder in tortured refugees: a preliminary uncontrolled trial. *Scandinavian journal of psychology, 51*(6), 495-502.

Halvorsen, J. O., Stenmark, H., Neuner, F., & Nordahl, H. M. (2014). Does dissociation moderate treatment outcomes of narrative exposure therapy for PTSD? A secondary analysis from a randomized controlled clinical trial. *Behaviour Research and Therapy, 57*, 21-28.

Hamid, S., Dashash, M., & Latifeh, Y. (2021). A short-term approach for promoting oral health of internally displaced children with PTSD: the key is improving mental health-results from a quasi-randomized trial. *BMC oral health, 21*(1), 58.

Hasanovic, M., Srabovic, S., Rasidovic, M., Sehovic, M., Hasanbasic, E., Husanovic, J., & Hodzic, R. (2009). Psychosocial assistance to students with posttraumatic stress disorder in primary and secondary schools in post-war Bosnia Herzegovina. *Psychiatria Danubina, 21*(4), 463-473.

Heim, E., Ramia, J. A., Hana, R. A., Burchert, S., Carswell, K., Cornelisz, I., . . . Van't Hof, E. (2021). Step-by-step: Feasibility randomised controlled trial of a mobile-based intervention for depression among populations affected by adversity in Lebanon. *Internet interventions, 24*, 100380.

Hendrickson, S. G. (2005). Reaching an underserved population with a randomly assigned home safety intervention. *Injury prevention : journal of the International Society for Child and Adolescent Injury Prevention, 11*(5), 313-317.

Hensel-Dittmann, D., Schauer, M., Ruf, M., Catani, C., Odenwald, M., Elbert, T., & Neuner, F. (2011). Treatment of traumatized victims of war and torture: a randomized controlled comparison of narrative exposure therapy and stress inoculation training. *Psychotherapy and psychosomatics, 80*(6), 345-352.

Hernandez, M. Y., & Organista, K. C. (2013). Entertainment-education? A fotonovela? A new strategy to improve depression literacy and help-seeking behaviors in at-risk immigrant Latinas. *American journal of community psychology, 52*(3), 224-235.

Hesselink, A. E., van Poppel, M. N., van Eijsden, M., Twisk, J. W. R., & van der Wal, M. F. (2012). The effectiveness of a perinatal education programme on smoking, infant care, and psychosocial health for ethnic Turkish women. *Midwifery, 28*(3), 306-313.

Hewage, K., Steel, Z., Mohsin, M., Tay, A. K., De Oliveira, J. C., Da Piedade, M., . . . Silove, D. (2018). A Wait-List Controlled Study of a Trauma-Focused Cognitive Behavioral Treatment for Intermittent Explosive Disorder in Timor-Leste. *American Journal of Orthopsychiatry, 88*(3), 282-294.

Hijazi, A. M., Lumley, M. A., Ziadni, M. S., Haddad, L., Rapport, L. J., & Arnetz, B. B. (2014). Brief narrative exposure therapy for posttraumatic stress in Iraqi refugees: a preliminary randomized clinical trial. *Journal of traumatic stress, 27*(3), 314-322.

Hinton, D. E., Chhean, D., Pich, V., Safren, S. A., Hofmann, S. G., & Pollack, M. H. (2005). A randomized controlled trial of cognitive-behavior therapy for Cambodian refugees with treatment-resistant PTSD and panic attacks: a cross-over design. *Journal of traumatic stress, 18*(6), 617-629.

Hinton, D. E., Hofmann, S. G., Pollack, M. H., & Otto, M. W. (2009). Mechanisms of efficacy of CBT for Cambodian refugees with PTSD: improvement in emotion regulation and orthostatic blood pressure response. *CNS neuroscience & therapeutics, 15*(3), 255-263.

Hinton, D. E., Hofmann, S. G., Rivera, E., Otto, M. W., & Pollack, M. H. (2011). Culturally adapted CBT (CA-CBT) for Latino women with treatment-resistant PTSD: A pilot study comparing CA-CBT to applied muscle relaxation. *Behaviour Research and Therapy, 49*(4), 275-280.

Holtrop, K., McNeil Smith, S., & Scott, J. C. (2015). Associations between positive parenting practices and child externalizing behavior in underserved Latino immigrant families. *Family process, 54*(2), 359-375.

Holzel, L. P., Ries, Z., Kriston, L., Dirmaier, J., Zill, J. M., Rummel-Kluge, C., . . . Harter, M. (2016). Effects of culture-sensitive adaptation of patient information material on usefulness in migrants: a multicentre, blinded randomised controlled trial. *BMJ open, 6*(11), e012008.

Hoskins, D., Duncan, L. G., Moskowitz, J. T., & Ordonez, A. E. (2018). Positive Adaptations for Trauma and Healing (PATH), a Pilot Study of Group Therapy With Latino Youth. *Psychological Trauma-Theory Research Practice and Policy, 10*(2), 163-172.

Hovey, J. D., Hurtado, G., & Seligman, L. D. (2014). Findings for a CBT Support Group for Latina Migrant Farmworkers in Western Colorado. *Current Psychology, 33*(3), 271-281.

Howes, C., Vu, J. A., & Hamilton, C. (2011). Mother-child attachment representation and relationships over time in Mexican-heritage families. *Journal of Research in Childhood Education, 25*(3), 228-247.

Hu, J., Wallace, D. C., McCoy, T. P., & Amirehsani, K. A. (2014). A family-based diabetes intervention for Hispanic adults and their family members. *The Diabetes educator, 40*(1), 48-59.

Huemer, J., Volkl-Kernstock, S., Yee, A., Bruckner, T., & Skala, K. (2016). "The Buoy": Utilization of a low-threshold ambulatory setting for traumatized children and adolescents in Austria. *Neuropsychiatrie : Klinik, Diagnostik, Therapie und Rehabilitation : Organ der Gesellschaft Osterreichischer Nervenarzte und Psychiater, 30*(1), 27-32.

Igreja, V., Kleijn, W. C., Schreuder, B. J. N., Van Dijk, J. A., & Verschuur, M. (2004). Testimony method to ameliorate post-traumatic stress symptoms - Community-based intervention study with Mozambican civil war survivors. *British Journal of Psychiatry, 184*, 251-257.

Im, H., Jettner, J. F., Warsame, A. H., Isse, M. M., Khoury, D., & Ross, A. I. (2018). Trauma-Informed Psychoeducation for Somali Refugee Youth in Urban Kenya: Effects on PTSD and Psychosocial Outcomes. *Journal of Child & Adolescent Trauma, 11*(4), 431-441.

Islam, N., Shapiro, E., Wyatt, L., Riley, L., Zanowiak, J., Ursua, R., & Trinh-Shevrin, C. (2017). Evaluating community health workers' attributes, roles, and pathways of action in immigrant communities. *Preventive medicine*, *103*, 1–7. https://doi.org/10.1016/j.ypmed.2017.07.020

Jalal, B., Kruger, Q., & Hinton, D. E. (2020). Culturally adapted CBT (CA-CBT) for traumatised indigenous South Africans (Sepedi): a randomised pilot trial comparing CA-CBT to applied muscle relaxation. *Intervention-International Journal of Mental Health Psychosocial Work and Counselling in Areas of Armed Conflict, 18*(1), 61-65. Retrieved from <Go to ISI>://WOS:000538043900008

Jang, Y., Chiriboga, D. A., Molinari, V., Roh, S., Park, Y., Kwon, S., & Cha, H. (2014). Telecounseling for the linguistically isolated: a pilot study with older Korean immigrants. *The Gerontologist, 54*(2), 290-296.

Javier, J. R., Reyes, A., Coffey, D. M., Schrager, S. M., Samson, A., Palinkas, L., . . . Miranda, J. (2019). Recruiting Filipino Immigrants in a Randomized Controlled Trial Promoting Enrollment in an Evidence-Based Parenting Intervention. *Journal of immigrant and minority health, 21*(2), 324-331.

Jin, Q., Mori, E., & Sakajo, A. (2020). Nursing intervention for preventing postpartum depressive symptoms among Chinese women in Japan. *Japan journal of nursing science : JJNS, 17*(4), e12336.

Jun, W. H., Hong, S. S., & Yang, S. (2014). Effects of a Psychological Adaptation Improvement Program for International Marriage Migrant Women in South Korea. *Asian Nursing Research, 8*(3), 232-238. Retrieved from <Go to ISI>://WOS:000343785800010

Kazandjian, C., Militello, L. K., & Doumit, R. (2020). Sex Differences on Quality of Life and Mental Health Outcomes When Using a Brief Cognitive-Behavioral Skill Building Intervention with Adolescent Syrian Refugees: A Secondary Analysis. *Community Mental Health Journal, 56*(1), 157-164.

Kha, J., Rapee, R. M., & Bayer, J. K. (2022). Acceptability and Outcomes of the Cool Little Kids Parenting Group Program for Culturally and Linguistically Diverse Families Within an Australian Population-Based Study. *Child Psychiatry and Human Development*.

Khawaja, N. G., Kamo, R., & Ramirez, E. (2021). Building resilience in transcultural adults: investigating the effect of a strength-based programme. *Australian Psychologist, 56*(4), 324-334.

Khawaja, N. G., & Ramirez, E. (2019). Building Resilience in Transcultural Adolescents: an Evaluation of a Group Program. *Journal of Child and Family Studies, 28*(11), 2977-2987.

Kalantari, M., Yule, W., Dyregrov, A., Neshatdoost, H., & Ahmadi, S. J. (2012). Efficacy of writing for recovery on traumatic grief symptoms of Afghani refugee bereaved adolescents: a randomized control trial. *Omega, 65*(2), 139-150.

Kaltman, S., Hurtado de Mendoza, A., Serrano, A., & Gonzales, F. A. (2016). A mental health intervention strategy for low-income, trauma-exposed Latina immigrants in primary care: A preliminary study. *The American journal of orthopsychiatry, 86*(3), 345-354.

Kaltman, S., Serrano, A., Talisman, N., Magee, M. F., Cabassa, L. J., Pulgar-Vidal, O., & Peraza, D. (2016). Type 2 Diabetes and Depression: A Pilot Trial of an Integrated Self-management Intervention for Latino Immigrants. *The Diabetes educator, 42*(1), 87-95.

Kaltman, S., Watson, M. R., Campoli, M., Serrano, A., Talisman, N., Kirkpatrick, L., . . . Green, B. L. (2019). Treatment of depression and PTSD in primary care clinics serving uninsured low-income mostly Latina/o immigrants: A naturalistic prospective evaluation. *Cultural diversity & ethnic minority psychology, 25*(4), 579-589.

Kananian, S., Soltani, Y., Hinton, D., & Stangier, U. (2020). Culturally Adapted Cognitive Behavioral Therapy Plus Problem Management (CA-CBT+) With Afghan Refugees: A Randomized Controlled Pilot Study. *Journal of traumatic stress, 33*(6), 928-938.

Kangaslampi, S., & Peltonen, K. (2020). Changes in Traumatic Memories and Posttraumatic Cognitions Associate with PTSD Symptom Improvement in Treatment of Multiply Traumatized Children and Adolescents. *Journal of child & adolescent trauma, 13*(1), 103-112.

Kelly, U. A., & Pich, K. (2014). Community-based PTSD treatment for ethnically diverse women who experienced intimate partner violence: a feasibility study. *Issues in mental health nursing, 35*(12), 906-913.

Kinzie, J. D., Kinzie, J. M., Sedighi, B., Woticha, A., Mohamed, H., & Riley, C. (2012). Prospective one-year treatment outcomes of tortured refugees: a psychiatric approach. *Torture : quarterly journal on rehabilitation of torture victims and prevention of torture, 22*(1), 1-10.

Kiropoulos, L. A., Griffiths, K. M., & Blashki, G. (2011). Effects of a multilingual information website intervention on the levels of depression literacy and depression-related stigma in Greek-born and Italian-born immigrants living in Australia: a randomized controlled trial. *Journal of medical Internet research, 13*(2), e34.

Kitchener, B. A., & Jorm, A. F. (2008). Mental Health First Aid: an international programme for early intervention. *Early intervention in psychiatry, 2*(1), 55-61.

Kneer, J., van Eldik, A. K., Jansz, J., Eischeid, S., & Usta, M. (2019). With a Little Help from My Friends: Peer Coaching for Refugee Adolescents and the Role of Social Media. *Media and Communication, 7*(2), 264-274.

Knefel, M., Kantor, V., Nicholson, A. A., Schiess-Jokanovic, J., Weindl, D., Schafer, I., & Lueger-Schuster, B. (2020). A brief transdiagnostic psychological intervention for Afghan asylum seekers and refugees in Austria: a randomized controlled trial. *Trials, 21*(1), 57.

Knox, L., Guerra, N. G., Williams, K. R., & Toro, R. (2011). Preventing children's aggression in immigrant Latino families: a mixed methods evaluation of the Families and Schools Together program. *American journal of community psychology, 48*(1), 65-76.

Kobel, S., Lammle, C., Wartha, O., Kesztyus, D., Wirt, T., & Steinacker, J. M. (2017). Effects of a Randomised Controlled School-Based Health Promotion Intervention on Obesity Related Behavioural Outcomes of Children with Migration Background. *Journal of immigrant and minority health, 19*(2), 254-262.

Kobel, S., Wirt, T., Schreiber, A., Kesztyus, D., Kettner, S., Erkelenz, N., . . . Steinacker, J. M. (2014). Intervention effects of a school-based health promotion programme on obesity related behavioural outcomes. *Journal of obesity, 2014*, 476230.

Koch, T., Ehring, T., & Liedl, A. (2020). Effectiveness of a transdiagnostic group intervention to enhance emotion regulation in young Afghan refugees: A pilot randomized controlled study. *Behaviour Research and Therapy, 132*, 103689.

Kocken, P. L., Zwanenburg, E. J.-v., & de Hoop, T. (2008). Effects of health education for migrant females with psychosomatic complaints treated by general practitioners. A randomised controlled evaluation study. *Patient education and counseling, 70*(1), 25-30.

Kodish, T., Weiss, B., Duong, J., Rodriguez, A., Anderson, G., Nguyen, H., . . . Lau, A. S. (2021). Interpersonal Psychotherapy-Adolescent Skills Training With Youth From Asian American and Immigrant Families: Cultural Considerations and Intervention Process. *Cognitive and Behavioral Practice, 28*(2), 147-166.

Kruse, J., Joksimovic, L., Cavka, M., Woller, W., & Schmitz, N. (2009). Effects of trauma-focused psychotherapy upon war refugees. *Journal of traumatic stress, 22*(6), 585-592.

Kwong, K., Chung, H., Cheal, K., Chou, J. C., & Chen, T. (2013). Depression care management for Chinese Americans in primary care: a feasibility pilot study. *Community mental health journal, 49*(2), 157-165.

Lancaster, S. L., & Gaede, C. (2020). A test of a resilience based intervention for mental health problems in Iraqi internally displaced person camps. *Anxiety, stress, and coping, 33*(6), 698-705.

Lau, A. S., Fung, J. J., Ho, L. Y., Liu, L. L., & Gudino, O. G. (2011). Parent training with high-risk immigrant chinese families: a pilot group randomized trial yielding practice-based evidence. *Behavior therapy, 42*(3), 413-426.

Le, H.-N., Perry, D. F., & Stuart, E. A. (2011). Randomized controlled trial of a preventive intervention for perinatal depression in high-risk Latinas. *Journal of consulting and clinical psychology, 79*(2), 135-141.

Le, H.-N., Perry, D. F., Villamil Grest, C., Genovez, M., Lieberman, K., Ortiz-Hernandez, S., & Serafini, C. (2020). A mixed methods evaluation of an intervention to prevent perinatal depression among Latina immigrants. *Journal of reproductive and infant psychology*, 1-13.

Lecerof, S. S., Stafstrom, M., Emmelin, M., Westerling, R., & Ostergen, P. O. (2017). Findings from a prospective cohort study evaluating the effects of International Health Advisors' work on recently settled migrants' health. *BMC public health, 17*.

Lee, P.-I., Lai, H.-R., Lin, P.-C., Kuo, S.-Y., Lin, Y.-K., Chen, S.-R., & Lee, P.-H. (2020). Effects of a parenting sexual education program for immigrant parents: A cluster randomized trial. *Patient education and counseling, 103*(2), 343-349.

Lee, E. J. (2015). The effect of positive group psychotherapy on self-esteem and state anger among adolescents at Korean immigrant churches. *Archives of Psychiatric Nursing, 29*(2), 108-113.

Lee, M.-K. (2003). *Filial therapy with immigrant Korean parents in the United States.* (63). ProQuest Information & Learning,

Leidy, M. S., Guerra, N. G., & Toro, R. I. (2010). Positive Parenting, Family Cohesion, and Child Social Competence Among Immigrant Latino Families. *Journal of Family Psychology, 24*(3), 252-260.

Leijten, P., Raaijmakers, M. A. J., Orobio de Castro, B., & Matthys, W. (2016). Ethnic differences in problem perception: Immigrant mothers in a parenting intervention to reduce disruptive child behavior. *The American journal of orthopsychiatry, 86*(3), 323-331.

Leiler, A., Wasteson, E., Holmberg, J., & Bjarta, A. (2020). A Pilot Study of a Psychoeducational Group Intervention Delivered at Asylum Accommodation Centers-A Mixed Methods Approach. *International journal of environmental research and public health, 17*(23).

Lempertz, D., Wichmann, M., Enderle, E., Stellermann-Strehlow, K., Pawils, S., & Metzner, F. (2020). Pre-post study to assess EMDR-based group therapy for traumatized refugee preschoolers. *Journal of EMDR Practice and Research, 14*(1), 31-45.

Lenglet, A., Lopes-Cardozo, B., Shanks, L., Blanton, C., Feo, C., Tsatsaeva, Z., . . . Pintaldi, G. (2018). Outcomes of an individual counselling programme in Grozny, Chechnya: a randomised controlled study. *BMJ open, 8*(8).

Leung, C., Tsang, S., & Dean, S. (2011). Outcome evaluation of the Hands-on Parent Empowerment (HOPE) program. *Research on Social Work Practice, 21*(5), 549-561.

Leung, C., Tsang, S., & Lo, C. (2017). Evaluation of Parent and Child Enhancement (PACE) program: Randomized controlled trial. *Research on Social Work Practice, 27*(1), 19-35.

Liedl, A., Muller, J., Morina, N., Karl, A., Denke, C., & Knaevelsrud, C. (2011). Physical activity within a CBT intervention improves coping with pain in traumatized refugees: results of a randomized controlled design. *Pain medicine (Malden, Mass.), 12*(2), 234-245.

Lindegaard, T., Seaton, F., Halaj, A., Berg, M., Kashoush, F., Barchini, R., . . . Andersson, G. (2021). Internet-based cognitive behavioural therapy for depression and anxiety among Arabic-speaking individuals in Sweden: *a pilot randomized controlled trial. Cogn Behav Ther, 50*(1), 47-66.

Litrownik, A.J., Elder, J.P., Campbell, N. R., Ayala, G. X., Slymen, D. J., Parra-Medina, D., . . .Lovato, C.Y. (2000). Evaluation of a tobacco and alcohol use prevention program for Hispanic migrant adolescents: promoting the protective factor of parent-child communication. Prev Med, 31(2 Pt 1), 124-133.

Lopez-Maya, E., Olmstead, R., & Irwin, M. R. (2019). Mindfulness meditation and improvement in depressive symptoms among Spanish- and English speaking adults: A randomized, controlled, comparative efficacy trial. *PloS one, 14*(7), e0219425.

Lou, N. M., & Noels, K. A. (2020). Breaking the vicious cycle of language anxiety: Growth language mindsets improve lower-competence ESL students' intercultural interactions. *Contemporary Educational Psychology, 61*. Retrieved from <Go to ISI>://WOS:000552133600005

Luelmo, P., Kasari, C., & Fiesta Educativa, I. (2021). Randomized pilot study of a special education advocacy program for Latinx/minority parents of children with autism spectrum disorder. *Autism*. Retrieved from <Go to ISI>://WOS:000644627100001

Lutenbacher, M., Elkins, T., Dietrich, M. S., & Riggs, A. (2018). The Efficacy of Using Peer Mentors to Improve Maternal and Infant Health Outcomes in Hispanic Families: Findings from a Randomized Clinical Trial. *Maternal and child health journal, 22*, 92-104.

Löfvander, M., Engström, A., Theander, H., & Furhoff, A. K. (1997). Rehabilitation of young immigrants in primary care. A comparison between two treatment models. *Scand J Prim Health Care, 15*(3), 123-128. doi:10.3109/02813439709018501

Magana, S., Hughes, M. T., Salkas, K., Gonzales, W., Nunez, G., Morales, M., . . . Moreno-Angarita, M. (2021) Implementing a Parent Education Intervention in Colombia: Assessing Parent Outcomes and Perceptions Across Delivery Modes. *Focus on Autism and Other Developmental Disabilities*. Retrieved from <Go to ISI>://WOS:000618515200001

Marksteiner, T., Janke, S., & Dickhauser, O. (2019). Effects of a brief psychological intervention on students' sense of belonging and educational outcomes: The role of students' migration and educational background. *Journal of school psychology, 75*, 41-57.

Marsiglia, F. F., Ayers, S. L., Baldwin-White, A., & Booth, J. (2016). Changing Latino Adolescents' Substance Use Norms and Behaviors: the Effects of Synchronized Youth and Parent Drug Use Prevention Interventions. *Prevention science : the official journal of the Society for Prevention Research, 17*(1), 1-12.

Marsiglia, F. F., Bermudez-Parsai, M., & Coonrod, D. (2010). Familias Sanas: an intervention designed to increase rates of postpartum visits among Latinas. *Journal of Health Care for the Poor and Underserved, 21*(3), 119-131.

Martinez, C. R., Jr., Eddy, J. M., McClure, H. H., & Cobb, C. L. (2022). Promoting Strong Latino Families Within an Emerging Immigration Context: Results of a Replication and Extension Trial of a Culturally Adapted Preventive Intervention. *Prevention science : the official journal of the Society for Prevention Research, 23*(2), 283-294.

Mateos-Fernandez, R., & Saavedra, J. (2020). Designing and assessing of an art-based intervention for undocumented migrants. *Arts & health*, 1-14.

McCabe, B. E., Mitchell, E. M., Gonzalez-Guarda, R. M., Peragallo, N., & Mitrani, V. B. (2017). Transnational Motherhood: Health of Hispanic Mothers in the United States Who Are Separated From Children. *Journal of transcultural nursing : official journal of the Transcultural Nursing Society, 28*(3), 243-250.

McDonald, L., Miller, H., & Sandler, J. (2015). A social ecological, relationship-based strategy for parent involvement: Families And Schools Together (FAST). *Journal of Childrens Services, 10*(3), 218-230. Retrieved from <Go to ISI>://WOS:000217782600004

McNaughton, D. B., Cowell, J. M., & Fogg, L. (2015). Efficacy of a Latino mother-child communication intervention in elementary schools. *The Journal of school nursing : the official publication of the National Association of School Nurses*, *31*(2), 126–134. https://doi.org/10.1177/1059840514526997

Meffert, S. M., Abdo, A. O., Alla, O. A. A., Elmakki, Y. O. M., Omer, A. A., Yousif, S., . . . Marmar, C. R. (2014). A pilot randomized controlled trial of interpersonal psychotherapy for Sudanese refugees in Cairo, Egypt. *Psychological Trauma: Theory, Research, Practice, and Policy, 6*(3), 240-249.

Mehrabi, T., Musavi, T., Ghazavi, Z., Zandieh, Z., & Zamani, A. (2011). The impact of group therapy training on social communications of Afghan immigrants. *Iranian journal of nursing and midwifery research, 16*(2), 148-152.

Meir, Y., Slone, M., & Levis, M. (2014). A randomized controlled study of a group intervention program to enhance mental health of children of illegal migrant workers. *Child & Youth Care Forum, 43*(2), 165-180.

Mendelsohn, A.L., Brockmeyer, C.A., Dreyer, B.P., Fierman, A.H., Berkule-Silberman, S.B. and Tomopoulos, S. (2010), Do verbal interactions with infants during electronic media exposure mitigate adverse impacts on their language development as toddlers?. Inf. Child Develop., 19: 577-593. <https://doi.org/10.1002/icd.711>

Metayer, N., Boulos, R., Tovar, A., Gervis, J., Abreu, J., Hval, E., . . . Economos, C. D. (2018). Recruitment of New Immigrants Into a Randomized Controlled Prevention Trial: The Live Well Experience. *The journal of primary prevention, 39*(5), 453-468.

Metzler, J., Diaconu, K., Hermosilla, S., Kaijuka, R., Ebulu, G., Savage, K., & Ager, A. (2019). Short- and longer-term impacts of Child Friendly Space Interventions in Rwamwanja Refugee Settlement, Uganda. *Journal of child psychology and psychiatry, and allied disciplines, 60*(11), 1152-1163.

Metzler, J., Jonfa, M., Savage, K., & Ager, A. (2021). Educational, psychosocial, and protection outcomes of child- and youth-focused programming with Somali refugees in Dollo Ado, Ethiopia. *Disasters, 45*(1), 67-85.

Miller, K. E., Koppenol-Gonzalez, G., Jawad, A., Steen, F., Sassine, M., & Jordans, M. (2020). A Randomised Controlled Trial of the I-Deal Life Skills Intervention with Syrian Refugee Adolescents in Northern Lebanon. *Intervention-International Journal of Mental Health Psychosocial Work and Counselling in Areas of Armed Conflict, 18*(2), 119-128. Retrieved from <Go to ISI>://WOS:000596156000004

Momartin, S., Coello, M., Pittaway, E., Downham, R., & Aroche, J. (2019). Capoeira Angola: An alternative intervention program for traumatized adolescent refugees from war-torn countries. *Torture : quarterly journal on rehabilitation of torture victims and prevention of torture, 29*(1), 85-96.

Morina, N., Ewers, S. M., Passardi, S., Schnyder, U., Knaevelsrud, C., Muller, J., . . . Schick, M. (2017). Mental health assessments in refugees and asylum seekers: evaluation of a tablet-assisted screening software. *Conflict and Health, 11*, 18.

Morville, A.-L., Erlandsson, L.-K., Danneskiold-Samsoe, B., Amris, K., & Eklund, M. (2015). Satisfaction with daily occupations amongst asylum seekers in Denmark. *Scandinavian journal of occupational therapy, 22*(3), 207-215.

Mucic, D. (2010). Transcultural telepsychiatry and its impact on patient satisfaction. *Journal of Telemedicine and Telecare, 16*(5), 237-242.

Murray, L. K., Hall, B. J., Dorsey, S., Ugueto, A. M., Puffer, E. S., Sim, A., . . . Bolton, P. A. (2018). An evaluation of a common elements treatment approach for youth in Somali refugee camps. *Global mental health (Cambridge, England), 5*, e16.

Naeem, F., Phiri, P., Munshi, T., Rathod, S., Ayub, M., Gobbi, M., & Kingdon, D. (2015). Using cognitive behaviour therapy with South Asian Muslims: Findings from the culturally sensitive CBT project. *International Review of Psychiatry, 27*(3), 233-246.

Neuner, F., Kurreck, S., Ruf, M., Odenwald, M., Elbert, T., & Schauer, M. (2010). Can asylum-seekers with posttraumatic stress disorder be successfully treated? A randomized controlled pilot study. *Cognitive behaviour therapy, 39*(2), 81-91.

Neuner, F., Onyut, P. L., Ertl, V., Odenwald, M., Schauer, E., & Elbert, T. (2008). Treatment of posttraumatic stress disorder by trained lay counselors in an African refugee settlement: a randomized controlled trial. *Journal of consulting and clinical psychology, 76*(4), 686-694.

Neuner, F., Schauer, M., Klaschik, C., Karunakara, U., & Elbert, T. (2004). A comparison of narrative exposure therapy, supportive counseling, and psychoeducation for treating posttraumatic stress disorder in an african refugee settlement. *Journal of consulting and clinical psychology, 72*(4), 579-587.

Nickerson, A., Byrow, Y., Pajak, R., McMahon, T., Bryant, R. A., Christensen, H., & Liddell, B. J. (2020). 'Tell Your Story': a randomized controlled trial of an online intervention to reduce mental health stigma and increase help-seeking in refugee men with posttraumatic stress. *Psychological Medicine, 50*(5), 781-792.

Niederer, I., Kriemler, S., Zahner, L., Burgi, F., Ebenegger, V., Hartmann, T., . . . Puder, J. J. (2009). Influence of a lifestyle intervention in preschool children on physiological and psychological parameters (Ballabeina): study design of a cluster randomized controlled trial. *BMC public health, 9*, 94.

Nordbrandt, M. S., Sonne, C., Mortensen, E. L., & Carlsson, J. (2020). Trauma-affected refugees treated with basic body awareness therapy or mixed physical activity as augmentation to treatment as usual-A pragmatic randomised controlled trial. *PloS one, 15*(3), e0230300.

Northwood, A. K., Vukovich, M. M., Beckman, A., Walter, J. P., Josiah, N., Hudak, L., . . . Danner, C. C. (2020). Intensive psychotherapy and case management for Karen refugees with major depression in primary care: a pragmatic randomized control trial. *BMC family practice, 21*(1), 17.

Nygren, T., Brohede, D., Koshnaw, K., Osman, S. S., Johansson, R., & Andersson, G. (2019). Internet-based treatment of depressive symptoms in a Kurdish population: A randomized controlled trial. *Journal of clinical psychology, 75*(6), 985-998.

Onyut, L. P., Neuner, F., Schauer, E., Ertl, V., Odenwald, M., Schauer, M., & Elbert, T. (2004). The Nakivale Camp Mental Health Project: Building local competency for psychological assistance to traumatised refugees. *Intervention: International Journal of Mental Health, Psychosocial Work & Counselling in Areas of Armed Conflict, 2*(2), 90-107.

Ooi, C. S., Rooney, R. M., Roberts, C., Kane, R. T., Wright, B., & Chatzisarantis, N. (2016). The Efficacy of a Group Cognitive Behavioral Therapy for War-Affected Young Migrants Living in Australia: A Cluster Randomized Controlled Trial. *Frontiers in psychology, 7*, 1641.

Opaas, M., & Hartmann, E. (2013). Rorschach assessment of traumatized refugees: an exploratory factor analysis. *Journal of personality assessment, 95*(5), 457-470.

Opaas, M., Wentzel-Larsen, T., & Varvin, S. (2020). The 10-year course of mental health, quality of life, and exile life functioning in traumatized refugees from treatment start. *PloS one, 15*(12), e0244730.

Oras, R., de Ezpeleta, S. C., & Ahmad, A. (2004). Treatment of traumatized refugee children with Eye Movement Desensitization and Reprocessing in a psychodynamic context. *Nordic journal of psychiatry, 58*(3), 199-203.

Otto, M. W., Hinton, D., Korbly, N. B., Chea, A., Ba, P., Gershuny, B. S., & Pollack, M. H. (2003). Treatment of pharmacotherapy-refractory posttraumatic stress disorder among Cambodian refugees: a pilot study of combination treatment with cognitive-behavior therapy vs sertraline alone. *Behaviour research and therapy, 41*(11), 1271-1276.

Page-Reeves, J., Murray-Krezan, C., Regino, L., Perez, J., Bleecker, M., Perez, D., . . . Willging, C. E. (2021). A randomized control trial to test a peer support group approach for reducing social isolation and depression among female Mexican immigrants. *BMC public health, 21*(1), 119.

Panter-Brick, C., Dajani, R., Eggerman, M., Hermosilla, S., Sancilio, A., & Ager, A. (2018). Insecurity, distress and mental health: experimental and randomized controlled trials of a psychosocial intervention for youth affected by the Syrian crisis. *Journal of child psychology and psychiatry, and allied disciplines, 59*(5), 523-541.

Pantin, H., Coatsworth, J. D., Feaster, D. J., Newman, F. L., Briones, E., Prado, G., . . . Szapocznik, J. (2003). Familias Unidas: the efficacy of an intervention to promote parental investment in Hispanic immigrant families. *Prevention science : the official journal of the Society for Prevention Research, 4*(3), 189-201.

Park, J. K., Park, J., Elbert, T., & Kim, S. J. (2020). Effects of Narrative Exposure Therapy on Posttraumatic Stress Disorder, Depression, and Insomnia in Traumatized North Korean Refugee Youth. *Journal of traumatic stress, 33*(3), 353-359.

Parra-Cardona, J. R., Bybee, D., Sullivan, C. M., Rodriguez, M. M. D., Dates, B., Tams, L., & Bernal, G. (2017). Examining the impact of differential cultural adaptation with Latina/o immigrants exposed to adapted parent training interventions. *Journal of consulting and clinical psychology, 85*(1), 58-71.

Parra-Cardona, R., Fuentes-Balderrama, J., Vanderziel, A., Lopez-Zeron, G., Domenech Rodriguez, M. M., DeGarmo, D. S., & Anthony, J. C. (2022). A Culturally Adapted Parenting Intervention for Mexican-Origin Immigrant Families with Adolescents: Integrating Science, Culture, and a Focus on Immigration-Related Adversity. *Prevention science : the official journal of the Society for Prevention Research, 23*(2), 271-282.

Paunovic, N., & Ost, L. G. (2001). Cognitive-behavior therapy vs exposure therapy in the treatment of PTSD in refugees. *Behaviour research and therapy, 39*(10), 1183-1197.

Peltonen, K., & Kangaslampi, S. (2019). Treating children and adolescents with multiple traumas: a randomized clinical trial of narrative exposure therapy. *European Journal of Psychotraumatology, 10*(1), 1558708.

Pfeiffer, E., & Goldbeck, L. (2017). Evaluation of a Trauma-Focused Group Intervention for Unaccompanied Young Refugees: A Pilot Study. *Journal of traumatic stress, 30*(5), 531-536.

Pfeiffer, E., Sachser, C., Rohlmann, F., & Goldbeck, L. (2018). Effectiveness of a trauma-focused group intervention for young refugees: a randomized controlled trial. *Journal of child psychology and psychiatry, and allied disciplines, 59*(11), 1171-1179.

Pfeiffer, E., Sachser, C., Tutus, D., Fegert, J. M., & Plener, P. L. (2019). Trauma-focused group intervention for unaccompanied young refugees: "Mein Weg"-predictors of treatment outcomes and sustainability of treatment effects. *Child and Adolescent Psychiatry and Mental Health, 13*, 18.

Piedra, L. M., & Byoun, S. J. (2012). Vida Alegre: Preliminary Findings of a Depression Intervention for Immigrant Latino Mothers. *Research on Social Work Practice, 22*(2), 138-150.

Pokhariyal, G. P., Rono, R., & Munywoki, S. (2013). Analysis of treatment methods for victims of torture in Kenya and East Africa Region. *Traumatology, 19*(2), 107-117.

Ponguta, L. A., Issa, G., Aoudeh, L., Maalouf, C., Hein, S. D., Zonderman, A. L., . . . Leckman, J. F. (2020). Effects of the Mother-Child Education Program on Parenting Stress and Disciplinary Practices Among Refugee and Other Marginalized Communities in Lebanon: A Pilot Randomized Controlled Trial. *Journal of the American Academy of Child and Adolescent Psychiatry, 59*(6), 727-738.

Poudel-Tandukar, K., Jacelon, C. S., Poudel, K. C., Bertone-Johnson, E. R., Rai, S., Ramdam, P., & Hollon, S. D. (2021). Mental health promotion among resettled Bhutanese adults in Massachusetts: Results of a peer-led family-centred Social and Emotional Well-being (SEW) intervention study. *Health & social care in the community*.

Priebe, S., Gavrilovic, J. J., Matanov, A., Franciskovic, T., Knezevic, G., Ljubotina, D., . . . Schutzwohl, M. (2010). Treatment Outcomes and Costs at Specialized Centers for the Treatment of PTSD After the War in Former Yugoslavia. *Psychiatric Services, 61*(6), 598-604.

Puccinelli, M. (2018). *Treatment and moderator effects in a randomized controlled trial of culturally informed and flexible family treatment for adolescents (CIFFTA): An investigation of the relationships between stress, acculturation, and parenting practices.* (79). ProQuest Information & Learning.

Qouta, S. R., Peltonen, K., Diab, S. Y., Anttila, S., Palosaari, E., & Punamaki, R. L. (2016). Psychosocial Intervention and Dreaming Among War-Affected Palestinian Children. *Dreaming, 26*(2), 95-118.

Quinlan, R., Schweitzer, R. D., Khawaja, N., & Griffin, J. (2016). Evaluation of a school-based creative arts therapy program for adolescents from refugee backgrounds. *The Arts in Psychotherapy*, *47*, 72-78.

Ramos, G., Blizzard, A. M., Barroso, N. E., & Bagner, D. M. (2018). Parent Training and Skill Acquisition and Utilization Among Spanish- and English-Speaking Latino Families. *Journal of Child and Family Studies, 27*(1), 268-279.

Rees, B., Travis, F., Shapiro, D., & Chant, R. (2013). Reduction in posttraumatic stress symptoms in Congolese refugees practicing transcendental meditation. *Journal of traumatic stress, 26*(2), 295-298.

Rees, B., Travis, F., Shapiro, D., & Chant, R. (2014). Significant reductions in posttraumatic stress symptoms in Congolese refugees within 10 days of Transcendental Meditation practice. *Journal of traumatic stress, 27*(1), 112-115.

Reijneveld, S. A., Westhoff, M. H., & Hopman-Rock, M. (2003). Promotion of health and physical activity improves the mental health of elderly immigrants: results of a group randomised controlled trial among Turkish immigrants in the Netherlands aged 45 and over. *Journal of epidemiology and community health, 57*(6), 405-411.

Renner, W. (2009). The effectiveness of psychotherapy with refugees and asylum seekers: preliminary results from an Austrian study. *Journal of immigrant and minority health*, *11*(1), 41–45. https://doi.org/10.1007/s10903-007-9095-1

Renner, W., Banninger-Huber, E., & Peltzer, K. (2011). Culture-Sensitive and Resource Oriented Peer (CROP)-Groups as a community based intervention for trauma survivors: A randomized controlled pilot study with refugees and asylum seekers from Chechnya. *Australasian Journal of Disaster and Trauma Studies, 2011*(1), 1-13.

Renner, W., & Berry, J. W. (2011). Group Interventions were not Effective for Female Turkish Migrants with Recurrent Depression - Recommendations from a Randomized Controlled Study. Social behavior and personality, 39 9, 1217-1234.

Renner, W., Laireiter, A.-R., & Maier, M. J. (2012). Social Support from Sponsorships as a Moderator of Acculturative Stress: Predictors of Effects on Refugees and Asylum Seekers. *Social Behavior and Personality, 40*(1), 129-146.

Riesch, S. K., Brown, R. L., Anderson, L. S., Wang, K., Canty-Mitchell, J., & Johnson, D. L. (2012). Strengthening Families Program (10-14): Effects on the Family Environment. *Western Journal of Nursing Research, 34*(3), 340-376.

Robjant, K., Roberts, J., & Katona, C. (2017). Treating Posttraumatic Stress Disorder in Female Victims of Trafficking Using Narrative Exposure Therapy: A Retrospective Audit. *Frontiers in Psychiatry, 8*.

Robl, M., de Souza, M., Schiel, R., Gellhaus, I., Zwiauer, K., Holl, R. W., & Wiegand, S. (2013). The key role of psychosocial risk on therapeutic outcome in obese children and adolescents. Results from a longitudinal multicenter study. *Obesity facts, 6*(3), 297-305.

Rohr, S., Jung, F. U., Pabst, A., Grochtdreis, T., Dams, J., Nagl, M., . . . Riedel-Heller, S. G. (2021). A Self-Help App for Syrian Refugees With Posttraumatic Stress (Sanadak): Randomized Controlled Trial. *JMIR mHealth and uHealth, 9*(1), e24807.

Rosser, R. L. (1986). Reality Therapy with the Khmer refugee resettled in the United States. *Journal of Reality Therapy, 6*(1), 21-29.

Rousseau, C., Beauregard, C., Daignault, K., Petrakos, H., Thombs, B. D., Steele, R., . . . Hechtman, L. (2014). A cluster randomized-controlled trial of a classroom-based drama workshop program to improve mental health outcomes among immigrant and refugee youth in special classes. *PloS one, 9*(8), e104704.

Rousseau, C., Benoit, M., Gauthier, M.-F., Lacroix, L., Alain, N., Rojas, M. V., . . . Bourassa, D. (2007). Classroom drama therapy program for immigrant and refugee adolescents: A pilot study. *Clinical Child Psychology and Psychiatry, 12*(3), 451-465.

Rousseau, C., Benoit, M., Lacroix, L., & Gauthier, M.-F. (2009). Evaluation of a sandplay program for preschoolers in a multiethnic neighborhood. *Journal of child psychology and psychiatry, and allied disciplines, 50*(6), 743-750.

Rousseau, C., Drapeau, A., Lacroix, L., Bagilishya, D., & Heusch, N. (2005). Evaluation of a classroom program of creative expression workshops for refugee and immigrant children. *Journal of child psychology and psychiatry, and allied disciplines, 46*(2), 180-185.

Ruf, M., Schauer, M., Neuner, F., Catani, C., Schauer, E., & Elbert, T. (2010). Narrative exposure therapy for 7- to 16-year-olds: a randomized controlled trial with traumatized refugee children. *Journal of traumatic stress, 23*(4), 437-445.

Salihu, D., Wong, E. M. L., & Kwan, R. Y. C. (2021). Effects of an African Circle Dance Programme on Internally Displaced Persons with Depressive Symptoms: A Quasi-Experimental Study. *International journal of environmental research and public health, 18*(2).

Sanchez-Aragon, A., Belzunegui-Eraso, A., & Prieto-Flores, O. (2020). Results of Mentoring in the Psychosocial Well-Being of Young Immigrants and Refugees in Spain. *Healthcare (Basel, Switzerland), 9*(1).

Sandahl, H., Carlsson, J., Sonne, C., Mortensen, E. L., Jennum, P., & Baandrup, L. (2021). Investigating the link between subjective sleep quality, symptoms of PTSD and level of functioning in a sample of trauma-affected refugees. *Sleep*.

Sandahl, H., Jennum, P., Baandrup, L., Lykke Mortensen, E., & Carlsson, J. (2021). Imagery rehearsal therapy and/or mianserin in treatment of refugees diagnosed with PTSD: Results from a randomized controlled trial. *Journal of sleep research*, e13276.

Sander, R., Laugesen, H., Skammeritz, S., Mortensen, E. L., & Carlsson, J. (2019). Interpreter-mediated psychotherapy with trauma-affected refugees - A retrospective cohort study. *Psychiatry research, 271*, 684-692.

Sarkadi, A., Adahl, K., Stenvall, E., Ssegonja, R., Batti, H., Gavra, P., . . . Salari, R. (2018). Teaching Recovery Techniques: evaluation of a group intervention for unaccompanied refugee minors with symptoms of PTSD in Sweden. *European Child & Adolescent Psychiatry, 27*(4), 467-479.

Scheiber, B., Greinz, G., Hillebrand, J. B., Wilhelm, F. H., & Blechert, J. (2019). Resilience training for unaccompanied refugee minors: A randomized controlled pilot study. *Resilienztraining fur unbegleitete minderjahrige Fluchtlinge: Eine randomisiert-kontrollierte Pilotstudie., 28*(3), 173-181.

Schick, M., Morina, N., Mistridis, P., Schnyder, U., Bryant, R. A., & Nickerson, A. (2018). Changes in Post-migration Living Difficulties Predict Treatment Outcome in Traumatized Refugees. *Frontiers in Psychiatry, 9*, 476.

Schneider, A., Pfeiffer, A., Conrad, D., Elbert, T., Kolassa, I. T., & Wilker, S. (2020). Does cumulative exposure to traumatic stressors predict treatment outcome of community-implemented exposure-based therapy for PTSD? *European Journal of Psychotraumatology, 11*(1).

Schnur, E., Koffler, R., Wimpenny, N., Giller, H., & et al. (1995). Family child care and new immigrants: Cultural bridge and support. *Special Issue: Child day care, 74*(6), 1237-1248.

Schottelkorb, A. A., Doumas, D. M., & Garcia, R. (2012). Treatment for childhood refugee trauma: A randomized, controlled trial. *International Journal of Play Therapy, 21*(2), 57-73.

Schulz, W., Bothe, T., & Hahlweg, K. (2018). Prevention of psychological disorders and behavioral problems in children and adolescents with migration background and their mothers: Results of a 10-year follow-up. *Pravention psychischer Probleme und Verhaltensauffalligkeiten von Kindern und Jugendlichen mit Migrationshintergrund und deren Muttern: Ergebnisse eines 10-Jahres-Follow-up., 28*(2), 82-92.

Schytt, E., Wahlberg, A., Eltayb, A., Small, R., Tsekhmestruk, N., & Lindgren, H. (2020). Community-based doula support for migrant women during labour and birth: study protocol for a randomised controlled trial in Stockholm, Sweden (NCT03461640). *BMJ open, 10*(2), e031290.

Shattell, M. M., Quinlan-Colwell, A., Villalba, J., Ivers, N. N., & Mails, M. (2010). A cognitive-behavioral group therapy intervention with depressed Spanish-speaking Mexican women living in an emerging immigrant community in the United States. *ANS. Advances in nursing science, 33*(2), 158-169.

Shaw, S. A., Ward, K. P., Pillai, V., & Hinton, D. E. (2019). A group mental health randomized controlled trial for female refugees in Malaysia. *The American journal of orthopsychiatry, 89*(6), 665-674.

Sherman, D. K., Hartson, K. A., Binning, K. R., Purdie-Vaughns, V., Garcia, J., Taborsky-Barba, S., . . . Cohen, G. L. (2013). Deflecting the trajectory and changing the narrative: how self-affirmation affects academic performance and motivation under identity threat. *Journal of personality and social psychology, 104*(4), 591-618.

Siddiqui, F., Lindblad, U., Nilsson, P. M., & Bennet, L. (2019). Effects of a randomized, culturally adapted, lifestyle intervention on mental health among Middle-Eastern immigrants. *European journal of public health, 29*(5), 888-894.

Slewa-Younan, S., McKenzie, M., Thomson, R., Smith, M., Mohammad, Y., & Mond, J. (2020). Improving the mental wellbeing of Arabic speaking refugees: an evaluation of a mental health promotion program. *BMC psychiatry, 20*(1).

Small, E., Kim, Y. K., Praetorius, R. T., & Mitschke, D. B. (2016). Mental health treatment for resettled refugees: A comparison of three approaches. *Social Work in Mental Health, 14*(4), 342-359.

Smokowski, P. R., & Bacallao, M. (2009a). Entre Dos Mundos/Between Two Worlds youth violence prevention: Comparing psychodramatic and support group delivery formats. *Small Group Research, 40*(1), 3-27.

Smokowski, P. R., & Bacallao, M. (2009b). Entre dos mundos/between two worlds: Youth violence prevention for acculturating Latino families. *Special Issue: Intervention outcome research with Latinos: Social work's contributions., 19*(2), 165-178.

Somasundaram, D. (2010). Using cultural relaxation methods in post-trauma care among refugees in Australia. *International Journal of Culture and Mental Health, 3*(1), 16-24.

Sonderegger, R., Rombouts, S., Ocen, B., & McKeever, R. S. (2011). Trauma rehabilitation for war-affected persons in northern Uganda: a pilot evaluation of the EMPOWER programme. *The British journal of clinical psychology, 50*(3), 234-249.

Sonne, C., Mortensen, E. L., Silove, D., Palic, S., & Carlsson, J. (2021). Predictors of treatment outcomes for trauma-affected refugees - results from two randomised trials. *Journal of affective disorders, 282*, 194-202.

Stade, K., Skammeritz, S., Hjortkjaer, C., & Carlsson, J. (2015). "After all the traumas my body has been through, I feel good that it is still working."--Basic Body Awareness Therapy for traumatised refugees. *Torture : quarterly journal on rehabilitation of torture victims and prevention of torture, 25*(1), 33-50.

Stanford, M. S., Elverson, T. M., Padilla, J. I., & Rogers, E. B. (2014). Feasibility and efficacy of a peer-led recovery group program for war-related trauma in Libya. *South African Journal of Psychology, 44*(1), 97-105.

Steinert, C., Bumke, P. J., Hollekamp, R. L., Larisch, A., Leichsenring, F., Matthess, H., . . . Kruse, J. (2017). Resource activation for treating post-traumatic stress disorder, co-morbid symptoms and impaired functioning: a randomized controlled trial in Cambodia. *Psychological Medicine, 47*(3), 553-564.

Stenmark, H., Catani, C., Neuner, F., Elbert, T., & Holen, A. (2013). Treating PTSD in refugees and asylum seekers within the general health care system. A randomized controlled multicenter study. *Behaviour research and therapy, 51*(10), 641-647.

Sternberg, R. M., Napoles, A. M., Gregorich, S., & Stewart, A. L. (2019). Mentes Positivas en Accion: Feasibility Study of a Promotor-Delivered Cognitive Behavioral Stress Management Program for Low-Income Spanish-Speaking Latinas. *Health Equity, 3*(1), 155-161.

Sternberg, R. M., Stewart, A. L., & Napoles, A. M. (2021). Mentes Positivas en Accion: A Randomized Feasibility Study of a Promotor-Delivered Cognitive Behavioral Stress Management Program for Low-Income Spanish-Speaking Latinos. *Health Equity, 5*(1), 218-226.

Su, S.-H., & Tsai, M.-H. (2016). Group play therapy with children of new immigrants in Taiwan who are exhibiting relationship difficulties. *Special Issue: International Journal of Play Therapy's 25th Anniversary: Play Therapy in Schools, 25*(2), 91-101.

Tay, A. K., Mung, H. K., Miah, M. A. A., Balasundaram, S., Ventevogel, P., Badrudduza, M., . . . Silove, D. (2020). An Integrative Adapt Therapy for common mental health symptoms and adaptive stress amongst Rohingya, Chin, and Kachin refugees living in Malaysia: A randomized controlled trial. *PLoS medicine, 17*(3), e1003073.

Thabet, A. A., Vostanis, P., & Karim, K. (2005). Group crisis intervention for children during ongoing war conflict. *European Child and Adolescent Psychiatry, 14*(5), 262-269.

Ter Heide, F. J. J., Mooren, T. M., Kleijn, W., de Jongh, A., & Kleber, R. J. (2011). EMDR versus stabilisation in traumatised asylum seekers and refugees: results of a pilot study. *European Journal of Psychotraumatology, 2*.

Ter Heide, F. J. J., Mooren, T. M., van de Schoot, R., de Jongh, A., & Kleber, R. J. (2016). Eye movement desensitisation and reprocessing therapy v. stabilisation as usual for refugees: randomised controlled trial. *The British journal of psychiatry : the journal of mental science, 209*(4), 311-318.

Tol, W. A., Leku, M. R., Lakin, D. P., Carswell, K., Augustinavicius, J., Adaku, A., . . . van Ommeren, M. (2020). Guided self-help to reduce psychological distress in South Sudanese female refugees in Uganda: a cluster randomised trial. *The Lancet. Global health, 8*(2), e254-e263.

Tucker, C., Schieffer, K., Lenz, S., & Smith, S. (2021). Sunshine Circles: Randomized controlled trial of an attachment-based play group with preschool students who are at-risk. *Journal of Child and Adolescent Counseling, 7*(3), 161-175.

Unger, J. B., Cabassa, L. J., Molina, G. B., Contreras, S., & Baron, M. (2013). Evaluation of a fotonovela to increase depression knowledge and reduce stigma among Hispanic adults. *Journal of immigrant and minority health, 15*(2), 398-406.

Unlu Ince, B., Cuijpers, P., van 't Hof, E., van Ballegooijen, W., Christensen, H., & Riper, H. (2013). Internet-based, culturally sensitive, problem-solving therapy for Turkish migrants with depression: randomized controlled trial. *Journal of medical Internet research, 15*(10), e227.

Unterhitzenberger, J., Wintersohl, S., Lang, M., Konig, J., & Rosner, R. (2019). Providing manualized individual trauma-focused CBT to unaccompanied refugee minors with uncertain residence status: a pilot study. *Child and Adolescent Psychiatry and Mental Health, 13*.

Ugurlu, N., Akca, L., & Acarturk, C. (2016). An art therapy intervention for symptoms of post-traumatic stress, depression and anxiety among Syrian refugee children. *Vulnerable Children and Youth Studies, 11*(2), 89-102.

van Heemstra, H. E., Scholte, W. F., Haagen, J. F. G., & Boelen, P. A. (2019). 7ROSES, a transdiagnostic intervention for promoting self-efficacy in traumatized refugees: a first quantitative evaluation. *European Journal of Psychotraumatology, 10*(1), 1673062.

van Loon, A., van Schaik, D. J. F., Dekker, J. J., & Beekman, A. T. F. (2011). Effectiveness of an intercultural module added to the treatment guidelines for Moroccan and Turkish patients with depressive and anxiety disorders. *BMC psychiatry, 11*, 13.

van Wyk, S., Schweitzer, R., Brough, M., Vromans, L., & Murray, K. (2012). A longitudinal study of mental health in refugees from Burma: the impact of therapeutic interventions. *The Australian and New Zealand journal of psychiatry, 46*(10), 995-1003.

Vijayakumar, L., Mohanraj, R., Kumar, S., Jeyaseelan, V., Sriram, S., & Shanmugam, M. (2017). CASP - An intervention by community volunteers to reduce suicidal behaviour among refugees. *The International journal of social psychiatry, 63*(7), 589-597.

Wagner, J., Kong, S., Kuoch, T., Scully, M. F., Tan, H. K., & Bermudez-Millan, A. (2015). Patient reported outcomes of Eat, Walk, Sleep: A cardiometabolic lifestyle program for Cambodian Americans delivered by community health workers. *Journal of Health Care for the Poor and Underserved, 26*(2), 441-452.

Walg, M., Angern, J. S., Michalak, J., & Hapfelmeier, G. (2020). [Effectiveness of stabilization training for adolescent refugees with trauma-induced disorders: A randomized controlled trial]. *Wirksamkeit des Stabilisierungstrainings fur jugendliche Fluchtlinge mit Traumafolgestorungen: Eine randomisierte Kontrollgruppenstudie., 48*(5), 369-379.

Wang, S. J., Bytyci, A., Izeti, S., Kallaba, M., Rushiti, F., Montgomery, E., & Modvig, J. (2017). A novel bio-psycho-social approach for rehabilitation of traumatized victims of torture and war in the post-conflict context: a pilot randomized controlled trial in Kosovo. *Conflict and Health, 10*.

Weine, S., Kulauzovic, Y., Klebic, A., Besic, S., Mujagic, A., Muzurovic, J., . . . Rolland, J. (2008). Evaluating a multiple-family group access intervention for refugees with PTSD. *Journal of marital and family therapy, 34*(2), 149-164.

Weine, S. M., Kulenovic, A. D., Pavkovic, I., & Gibbons, R. (1998). Testimony psychotherapy in Bosnian refugees: a pilot study. *The American journal of psychiatry, 155*(12), 1720-1726.

Weinstein, N., Khabbaz, F., & Legate, N. (2016). Enhancing need satisfaction to reduce psychological distress in Syrian refugees. *Journal of consulting and clinical psychology, 84*(7), 645-650.

Weisleder, A., Cates, C. B., Dreyer, B. P., Berkule Johnson, S., Huberman, H. S., Seery, A. M., . . . Mendelsohn, A. L. (2016). Promotion of Positive Parenting and Prevention of Socioemotional Disparities. *Pediatrics, 137*(2), e20153239.

Weiss, W. M., Murray, L. K., Zangana, G. A. S., Mahmooth, Z., Kaysen, D., Dorsey, S., . . . Bolton, P. (2015). Community-based mental health treatments for survivors of torture and militant attacks in Southern Iraq: a randomized control trial. *BMC psychiatry, 15*.

Williamson, A. A., Knox, L., Guerra, N. G., & Williams, K. R. (2014). A pilot randomized trial of community-based parent training for immigrant Latina mothers. *American journal of community psychology, 53*(1), 47-59.

Yagmur, S., Mesman, J., Malda, M., Bakermans-Kranenburg, M. J., & Ekmekci, H. (2014). Video-feedback intervention increases sensitive parenting in ethnic minority mothers: a randomized control trial. *Attachment & human development, 16*(4), 371-386.

Yankey, T., & Biswas, U. N. (2019). Impact of life skills training on psychosocial well-being of Tibetan refugee adolescents. *International Journal of Migration Health and Social Care, 15*(4), 272-284.

Yeung, A., Martinson, M. A., Baer, L., Chen, J., Clain, A., Williams, A., . . . Fava, M. (2016). The Effectiveness of Telepsychiatry-Based Culturally Sensitive Collaborative Treatment for Depressed Chinese American Immigrants: A Randomized Controlled Trial. *The Journal of clinical psychiatry, 77*(8), e996-e1002.

Yeung, A., Shyu, I., Fisher, L., Wu, S., Yang, H., & Fava, M. (2010). Culturally sensitive collaborative treatment for depressed chinese americans in primary care. *American journal of public health*, *100*(12), 2397–2402.

Young, M., Salerno, J., Rockhill, S., Hernandez, A., & DeMaria, R. (2021). Evaluation of the Impact of a Healthy Relationship Program Among US Refugees. *Family relations, 70*(5), 1643-1656.

Yu, N. X., Lam, T. H., Liu, I. K. F., & Stewart, S. M. (2015). Mediation of Short and Longer Term Effects of an Intervention Program to Enhance Resilience in Immigrants from Mainland China to Hong Kong. *Frontiers in psychology, 6*.

Yu, X. N., Stewart, S. M., Chui, J. P. L., Ho, J. L. Y., Li, A. C. H., & Lam, T. H. (2014). A Pilot Randomized Controlled Trial to Decrease Adaptation Difficulties in Chinese New Immigrants to Hong Kong. *Behavior Therapy, 45*(1), 137-152.

Yurtsever, A., Konuk, E., Akyuz, T., Zat, Z., Tukel, F., Cetinkaya, M., . . . Shapiro, E. (2018). An Eye Movement Desensitization and Reprocessing (EMDR) Group Intervention for Syrian Refugees With Post-traumatic Stress Symptoms: Results of a Randomized Controlled Trial. *Frontiers in psychology, 9*, 493.

Zapata, G. P., & Hargreaves, D. J. (2018). The effects of musical activities on the self-esteem of displaced children in Colombia. *Psychology of Music, 46*(4), 540-550.

Zehetmair, C., Kaufmann, C., Tegeler, I., Kindermann, D., Junne, F., Zipfel, S., . . . Nikendei, C. (2018). Psychotherapeutic Group Intervention for Traumatized Male Refugees Using Imaginative Stabilization Techniques-A Pilot Study in a German Reception Center. *Frontiers in Psychiatry, 9*.

**Wrong publication type**

Durbeej, N., McDiarmid, S., Sarkadi, A., Feldman, I., Punamaki, R.-L., Kankaanpaa, R., . . . Osman, F. (2021). Evaluation of a school-based intervention to promote mental health of refugee youth in Sweden (The RefugeesWellSchool Trial): study protocol for a cluster randomized controlled trial. *Trials, 22*(1), 98.

Lachal, J., Moro, M. R., Carretier, E., Simon, A., Barry, C., Falissard, B., & Rouquette, A. (2020). Assessment of transcultural psychotherapy to treat resistant major depressive disorder in children and adolescents from migrant families: Protocol for a randomized controlled trial using mixed method and Bayesian approaches. *International journal of methods in psychiatric research, 29*(4), 1-10.

Lebiger-Vogel, J., Rickmeyer, C., Busse, A., Fritzemeyer, K., Ruger, B., & Leuzinger-Bohleber, M. (2015). FIRST STEPS - a randomized controlled trial on the evaluation of the implementation and effectiveness of two early prevention programs for promoting the social integration and a healthy development of children with an immigrant background from 0-3. *BMC psychology, 3*(1), 21.

Leckman, J. F. (2020).Effects of the Mother-Child Education Program on Parenting Stress and Disciplinary Practices among refugee and other marginalized communities in Lebanon: A Pilot Randomized Controlled Trial. *Journal of the American Academy of Child and Adolescent Psychiatry, 59*(10), S118-S119.

Nathan, S., Bunde-Birouste, A., Evers, C., Kemp, L., MacKenzie, J., & Henley, R. (2010). Social cohesion through football: a quasi-experimental mixed methods design to evaluate a complex health promotion program. *BMC public health, 10*, 587.

**Wrong study design**

Bjorknes, R., Kjobli, J., Manger, T., & Jakobsen, R. (2012). Parent training among ethnic minorities: Parenting practices as mediators of change in child conduct problems. *Family Relations: An Interdisciplinary Journal of Applied Family Studies, 61*(1), 101-114.

Brown, R. C., Witt, A., Fegert, J. M., Keller, F., Rassenhofer, M., & Plener, P. L. (2017). Psychosocial interventions for children and adolescents after man-made and natural disasters: a meta-analysis and systematic review. *Psychological Medicine, 47*(11), 1893-1905.

Edwards, B., Smart, D., De Maio, J., Silbert, M., & Jenkinson, R. (2018). Cohort Profile: Building a New Life in Australia (BNLA): the longitudinal study of humanitarian migrants. *International journal of epidemiology, 47*(1), 20-20h.

Fuchs, C., Lee, J. K., Roemer, L., & Orsillo, S. M. (2013). Using Mindfulness- and Acceptance-Based Treatments With Clients From Nondominant Cultural and/or Marginalized Backgrounds: Clinical Considerations, Meta-Analysis Findings, and Introduction to the Special Series. *Cognitive and Behavioral Practice, 20*(1), 1-12.

Hein, S., Bick, J., Issa, G., Aoude, L., Maalouf, C., Awar, A., . . . Ponguta, L. A. (2020). Maternal perceptions of father involvement among refugee and disadvantaged families in Beirut, Lebanon. *PloS one, 15*(3), e0229670.

Kayrouz, R., Dear, B. F., Kayrouz, B., Karin, E., Gandy, M., & Titov, N. (2018). Meta-analysis of the efficacy and acceptability of cognitive-behavioural therapy for Arab adult populations experiencing anxiety, depression or post-traumatic stress disorder. *Cognitive Behaviour Therapy, 47*(5), 412-430.

Wade-Bohleber, L., Hofer, A., Ottiger, M., von Wyl, A., Stulz, A., & Rumpel, S. (2022). [Can the GroupTherapy "Arriving" Support Refugee Mothers with their Young Children? Results from a Longitudinal Pilot Study]. *Aacho" - ein niederschwelliges gruppentherapeutisches Angebot fur gefluchtete Mutter mit Kleinkindern: Ergebnisse einer evaluativen Pilotstudie., 71*(2), 119-140.

**Wrong intervention**

O'Leary, P., Hutchinson, A., & Squire, J. (2015). Community-based child protection with Palestinian refugees in South Lebanon: Engendering hope and safety. *International Social Work, 58*(5), 717-731.

**Too few participants**

Rondung, E., Leiler, A., Sarkadi, A., Bjarta, A., Lampa, E., Lofving, S. G., . . . Warner, G. (2022). Feasibility of a randomised trial of Teaching Recovery Techniques (TRT) with refugee youth: results from a pilot of the Swedish UnaccomPanied yOuth Refugee Trial (SUPpORT). *Pilot and Feasibility Studies, 8*(1), 40.

**No access to data**

Staæhr, M. A. (2001). Psykoedukation med Kosovo Albanske flygtningebørn: En effektundersøgelse af et program til forebyggelse af alvorlige psykiske belastningsreaktioner = Psychoeducation with Kosovo Albanian refugee children. *Psyke & Logos, 22*(1), 127-146.

Lawrence, K. C., & Falaye, A. O. (2020). Trauma-focused counselling and social effectiveness skills training interventions on impaired psychological functioning of internally displaced adolescents in Nigeria. *Journal of Community & Applied Social Psychology, 30*(6), 616-627.

**Not retrieved**

de Kom, A. A., & Bleeker, J. A. (1991). Acute reactive psychosis among immigrants in Amsterdam. *Lancet (London, England), 337*(8734), 185-186.

Fox, P. G., Cowell, J. M., Montgomery, A. C., & Willgerodt, M. A. (1998). Southeast Asian refugee women and depression: a nursing intervention. *The international journal of psychiatric nursing research, 4*(1), 423-432.

Silove, D., Chang, R., & Manicavasagar, V. (1995). Impact of recounting trauma stories on the emotional state of Cambodian refugees. *Psychiatric services (Washington, D.C.), 46*(12), 1287-1288.

**Appendix 5: Forest plots of between group analysis of primary and secondary outcomes**

| Child externalizing behaviours | Child internalizing behaviours |
| --- | --- |
|  |  |
| Positive parenting strategies | Negative parenting strategies |
|  |  |
| Parental self-efficacy | Psychological distress |
|  |  |

**Appendix 6: Forest plots of within-group analysis of primary and secondary outcomes of parent interventions**

| Child externalizing behaviours (parent rated) |
| --- |
| **** |
| Child internalizing behaviours (parent rated) |
|  |
| Negative parenting strategies (parent rated) |
|  |
| Positive parenting strategies (parent rated) |
|  |
| Self-efficacy (parent) |
| **** |
| Well-being (parent) |
| **** |
| Psychological distress (parent) |
| **** |
| Anxiety (parent) |
| **** |
| Depression (parent) |
| **** |
| PTSD (parent) |
| **** |
| Child internalizing behaviours (child rated) |
|  |
| Negative parenting (child rated) |
| **** |

**Appendix 7: Forest plots of combined interventions**

| Child internalizing behaviours, between group (parent rated) | Negative parenting, between group (parent rated) |
| --- | --- |
|  |  |
| Positive parenting, between group (parent rated) | Depression, between group (child rated) |
|  |  |
| Externalizing behaviours, between group (child rated) | Child externalizing behaviours, within group  (parent rated) |
|  |  |
| Positive parenting, within group (parent rated) | Negative parenting, within group  (parent rated) |
|  |  |

**Appendix 8: Certainty in evidence using GRADE – parenting interventions**

**Authors:** Västhagen, M., Giles, C. J., Hollander, A-C, Van Leuven, L., Edenius, A., Ghaderi, A. & Enberink, P.

**Question:** Parenting programs compared to all comparators for improving parenting and psychological well-being

**Setting:** Non clinical

| **Outcomes Assessed with (measure) Included studies** | **Certainty assessment** | | | | | | | **Certainty** |
| --- | --- | --- | --- | --- | --- | --- | --- | --- |
|  | **№ of studies** | **Study design** | **Risk of bias** | **Inconsistency** | **Indirectness** | **Imprecision** | **Other considerations** |  |
| **Child externalizing behaviours** CBCL, Eyberg  Bjorknes et al, 2013; Osman, Flacking et al, 2017 | 2 | randomised trials | serious^a^ | not serious | not serious | not serious^b^ | none | ⨁⨁⨁◯ Moderate |
| **Child internalizing behaviours** CBCL, Child problems Dybdahl, 2001; Osman, Flacking et al,2017 | 2 | randomised trials | serious^c^ | not serious | not serious | serious^d^ | none | ⨁⨁◯◯ Low |
| **Positive parenting** PPI, APQ Bjorknes et al, 2013; Shaw et al, 2021 | 2 | randomised trials | serious^e^ | serious^f^ | not serious | serious^g^ | none | ⨁◯◯◯ Very low |
| **Negative parenting**  PPI, APQ Bjorknes et al, 2013; Shaw et al, 2021 | 2 | randomised trials | serious^e^ | not serious | not serious | serious^h^ | none | ⨁⨁◯◯ Low |
| **Self-Efficacy** PSOC, CAPES Osman, Salari et al, 2017; Shaw et al, 2021 | 2 | randomised trials | serious^i^ | not serious | not serious | not serious | none | ⨁⨁⨁◯ Moderate |
| **Psychological distress**  HCL, Kessler-10, GHQ, RHS-15 Bjorknes et al, 2015; Miller et al, 2020; Osman, Salari et al, 2017; Shaw et al, 2021 | 4 | randomised trials | serious^j^ | serious^k^ | not serious | serious^l^ | none | ⨁◯◯◯ Very low |

**CI:** confidence interval

#### Explanations

a. No pre-specified data-analysis plan. No blinded assessors (self-rating scales) which is expected within this field.

b. The CI does not include 0, suggesting a little chance of negative effects.

c. Dybdahl, 2001: lack of control and description of missing data and lack of a pre-defined analysis plan. Osman et al, 2017: No pre-specified data-analysis plan and no blinded assessors (self-rating scales) which is expected within the field.

d. Power calculation for Osman, Flacking et al. (2017) was estimated to 128 and 109 particpants were included in the study, Dybdahl et al. (2001) had fewer participants (n = 75) but did not present any power calculations. CI including 0, suggesting a chance of both negative and positive effects.

e. Neither studies had a pre-specified data-analysis plan, and there were no blinded assessors. In the study of Shaw et al, 2021 there was also a large drop-out between randomization and intervention start.

f. Point estimates do not overlap with the confidence intervals of other studies. Considerable width in confidence intervals (and the combined confidence interval (1.5)), and statistical measures of heterogeneity are high. However, still a clear positive effect, and there is overlap in the width of the confidence intervals.

g. Bjorknes et al. (2013) included 96 participants but did not present any power calculations. Shaw et al. (2021) includes a larger sample size (n = 137) but did neither present any power calculations. A wide CI but witch not include 0, so little risk of negative effect.

h. Bjorknes et al. (2013) included 96 participants but did not present any power calculations. Shaw et al. (2021) included a larger sample size (n = 137) but did not present any power calculations either. A wide CI witch includes 0, suggesting chances of both positive and negative effects.

i. None of the studies had a pre-specified data-analysis plan, and there were no blinded assessors. In the study of Shaw et al, 2021 there was also a large drop-out between randomization and intervention start.

j. None of the studies had a pre-specified data-analysis plan, and there were no blinded assessors. Also, Shaw et al. (2021) had a high level of drop-out between randomization and intervention start.

k. Point estimates differ considerably, there is little overlap in confidence intervals for some studies (and the combined confidence interval), and statistical measures of heterogeneity is high.

l. A relatively wide CI but clear positive effect.

‘’

**Appendix 9: Certainty in evidence using GRADE – combined interventions**

**Authors:** Västhagen, M., Giles, C. J., Hollander, A-C, Van Leuven, L., Edenius, A., Ghaderi, A. & Enberink, P.

**Question:** Combined interventions (parent + youth) compared to all comparators for improving parenting and psychological well-being?

**Setting:** Non clinical

| **Outcomes Assessed with (measure) Included studies** | **Certainty assessment** | | | | | | | **Certainty** |
| --- | --- | --- | --- | --- | --- | --- | --- | --- |
|  | **№ of studies** | **Study design** | **Risk of bias** | **Inconsistency** | **Indirectness** | **Imprecision** | **Other considerations** |  |
| **Depression (child rated)**  PHQ, Centre for Epidemiology studies Depression Scale for Children  Ahktar et al, 2021; Betancourt et al, 2020 | 2 | randomised trials | serious^a^ | not serious | not serious | serious^b^ | none | ⨁⨁◯◯ Low |
| **Externalizing behaviours (child rated)**  PSC-35, Achenbach Youth Self Report  Ahktar et al, 2021; Betancourt et al, 2020 | 2 | randomised trials | serious^a^ | very serious^c^ | not serious | serious^d^ | none | ⨁◯◯◯ Very low |
| **Child internalizing behaviours (parent rated)**  Center for Epidemiology Studies Depression Scale for Children, PSC-35 Ahktar et al, 2021; Betancourt et al, 2020 | 2 | randomised trials | serious^a^ | very serious^e^ | not serious | serious^f^ | none | ⨁◯◯◯ Very low |
| **Positive parenting**  Discipline interview, APQ  Ahktar et al, 2021; Puffer et al, 2017 | 2 | randomised trials | serious^g^ | serious^h^ | not serious | serious^i^ | none | ⨁◯◯◯ Very low |
| **Negative parenting**  Discipline interview, APQ  Ahktar et al, 2021; Puffer et al, 2017 | 2 | randomised trials | serious^g^ | very serious^j^ | not serious | Serious^k^ | none | ⨁◯◯◯ Very low |

**CI:** confidence interval

#### Explanations

a. Akhtar et al. (2021) had low RoB, Betancourt et al (2020) had some risk of bias due to drop-out and no pre-specified data analysis plan.

b. A relatively wide CI which includes 0, suggesting considerable chance of either positive and negative effects.Ahktar et al. (2021) included a sample of 113 caregivers and children and Betancourt et al. (2020) included 111 caregivers and childen, none of the studies presented power calculations.

c. Point estimates vary considerably (and are on opposite sides of 0), there is some overlap in confidence intervals (and the combined confidence interval), but they do not include respective mean of each study, and statistical measures of heterogeneity are high.

d. Ahktar et al. (2021) included a sample of 113 caregivers and children and Betancourt et al. (2020) included 111 caregivers and childen, none of the studies presented power calculations. A wide CI, including 0, suggesting considerable chance of either positive and negative effects.

e. Ahktar et al. (2021) included a sample of 113 caregivers and children and Betancourt et al. (2020) included 111 caregivers and childen, none of the studies presented power calculations. A quite wide CI which does not cross 0, suggesting little risk of negative effects.

e. Point estimates differ considerably, there is little overlap in confidence intervals for some studies (and the combined confidence interval), and no overlap in CI over point estimate for other study, and statistical measures of heterogeneity is high.

f. A wide CI that includes 0.

g. Akhtar et al., (2021) had low RoB, Puffer et al., (2017) has no pre-defined analysis plan or participant/assessor blinding.

h. There is complete overlap in confidence intervals (and with the combined confidence interval). Reach of Akhtar’s CI is very large and crosses 0. Statistical measures of heterogeneity is low.

i. Large sample size in Puffer et al. (2017), *n* = 479 (power = 0.8) but no power calculations were calculated for Ahktar et al. 2021 (*n* = 57), CI that includes 0.

j. Point estimates differ considerably, there is complete overlap in confidence intervals (and the combined confidence interval) but no overlap in Puffer's CI, or the combined CI and Akhtar’s point estimate. Width of Akhtar’s CI is very large and crosses 0. Statistical measures show some heterogeneity.

k. Large sample size in Puffer et al. (2017), *n* = 479 (power = 0.8). No power calculations were calculated for Ahktar et al. 2021 (*n* = 57). Quite wide CI that includes 0.
